# Aluminiumverbindungen, lösliche: MAK-Begründung

**DOI:** 10.34865/mb742990verdb10_3or

**Published:** 2025-09-29

**Authors:** Andrea Hartwig, MAK Commission

**Affiliations:** 1 Institut für Angewandte Biowissenschaften. Abteilung Lebensmittelchemie und Toxikologie. Karlsruher Institut für Technologie (KIT) Adenauerring 20a, Geb. 50.41 76131 Karlsruhe Deutschland; 2 Ständige Senatskommission zur Prüfung gesundheitsschädlicher Arbeitsstoffe. Deutsche Forschungsgemeinschaft, Kennedyallee 40, 53175 Bonn, Deutschland. Weitere Informationen: Ständige Senatskommission zur Prüfung gesundheitsschädlicher Arbeitsstoffe | DFG

**Keywords:** Aluminium, lösliche Aluminiumverbindungen, Lunge, Entzündung, Reizwirkung, MAK-Wert, maximale Arbeitsplatzkonzentration, Spitzenbegrenzung, Entwicklungstoxizität, aluminium, soluble aluminium compounds, lung, inflammation, irritation, MAK value, maximum workplace concentration, peak limitation, developmental toxicity

## Abstract

The German Senate Commission for the Investigation of Health Hazards of Chemical Compounds in the Work Area (MAK Commission) summarized and evaluated the data for soluble aluminium compounds to derive an occupational exposure limit value (maximum concentration at the workplace, MAK value) considering all toxicological end points. Relevant studies were identified from a literature search and unpublished study reports were used. Aluminium chlorohydrate, which is at most slightly irritating to the eye, shows irritant effects to the lungs of rats and guinea pigs after six months inhalational exposure to 0.08 mg Al/m^3^. Based on this study, a MAK value of 0.005 mg Al/m^3^ is set for aluminium chlorohydrate. This MAK value also applies to aluminium ammonium disulfate and aluminium potassium disulfate, which are not irritating to the eyes. In contrast, aluminium chloride, which is corrosive to the eye, is a strong lung irritant in rats after 13 weeks of inhalation of 0.02 mg Al/m^3^. From this study, a MAK value of 0.0002 mg Al/m^3^ for the inhalable fraction is derived, which also applies to basic aluminium chloride, aluminium chloride hydroxysulfate, aluminium diacetate, aluminium lactate, aluminium nitrate and aluminium sulfate, as these substances are also irritating or irreversibly damaging to the eyes. These compounds are assigned to peak limitation category I with an excursion factor of 2. For aluminium gluconate and aluminium maltolate, no data are available on effects on the eyes or the respiratory tract, therefore, as a conservative approach, the MAK value of 0.0002 mg Al/m^3^ is also set for these substances. These compounds are assigned to peak limitation category II with an excursion factor of 2. The MAK values also protect from neurotoxicity as seen for poorly soluble aluminium compounds in humans. In vitro data show no mutagenicity of soluble aluminium compounds. No micronuclei or chromosomal aberrations are induced in valid in vivo studies below cytotoxic concentrations. In vitro and in vivo clastogenic and aneugenic effects and positive indicator tests are predominantly observed at cytotoxic concentrations. The available human data do not support an association between the development of breast cancer and the use of aluminium-containing antiperspirants. There are no valid long-term carcinogenicity studies in animals. Based on the MAK values of 0.0002 and 0.005 mg Al/m^3^, only a slight increase in the urinary aluminium concentration of up to 0.2 % and 4.0 % in relation to the biological reference value (BAR) of 15 µg aluminium/g creatinine is calculated. Therefore, soluble aluminium compounds are assigned to Pregnancy Risk Group C. Skin contact is not expected to contribute significantly to systemic toxicity. A sensitizing potential is not expected from the data available.

**Table:** 

**Aluminiumammoniumdisulfat,** **Aluminiumchlorhydrat,** **Aluminiumkaliumdisulfat**	
**MAK-Wert (2024)**	**0,005 mg Al/m^3^ E**
**Spitzenbegrenzung (2024)**	**Kategorie II, Überschreitungsfaktor 2**
**Hautresorption**	**–**
**Sensibilisierende Wirkung**	**–**
**Krebserzeugende Wirkung**	**–**
**Fruchtschädigende Wirkung (2024)**	**Gruppe C**
**Keimzellmutagene Wirkung**	**–**
	
**Aluminiumchlorid,** **Aluminiumchlorid, basisch,** **Aluminiumchloridhydroxysulfat,** **Aluminiumcitrat,** **Aluminiumdiacetat,** **Aluminiumgluconat,** **Aluminiumlactat,** **Aluminiummaltolat,** **Aluminiumnitrat,** **Aluminiumsulfat**	
**MAK-Wert (2024)**	**0,0002 mg Al/m^3^ E**
**Spitzenbegrenzung (2024)**	**Kategorie I, Überschreitungsfaktor 2**
**Hautresorption**	**–**
**Sensibilisierende Wirkung**	**–**
**Krebserzeugende Wirkung**	**–**
**Fruchtschädigende Wirkung (2024)**	**Gruppe C**
**Keimzellmutagene Wirkung**	**–**
	
**BAT-Wert (2017)**	**50 µg Aluminium/g Kreatinin**
**BAR (2018)**	**15 µg Aluminium/g Kreatinin**
	
Hydrolysestabilität	Lösliche Aluminiumverbindungen sind hydrolytisch instabil. Al^3+^ bildet mit Wasser einen Komplex. Bei pH-Werten < 4,5 liegt Al^3+^ als Hexahydrat-Komplex (Al^3+^ × 6 H_2_O) vor. Bei steigendem pH-Wert erfolgt eine Deprotonierung zu Aluminiumhydroxid (Dekant 2019); **Aluminiumchlorid**: wasserfreies AlCl_3_ hydratisiert bei Kontakt mit der Feuchtigkeit in der Luft extrem schnell; Hauptreaktionsprodukt in wässriger Lösung ist Aluminiumchloridhexahydrat (AlCl_3_ × 6 H_2_O) (BASF SE 2018); **Aluminiumchlorid, basisch**: liegt bereits als wässrige Lösung vor (ECHA 2023 a); **Aluminiumchloridhydroxysulfat**: hydrolytisch instabil (ECHA 2020 b); **Aluminiumchlorhydrat**: hydrolytisch instabil, Halbwertszeit (HWZ) < 12 h (ECHA 2022 c); **Aluminiumkaliumdisulfat**: dissoziiert in Wasser zu Al^3+^, K^+^ und SO_4_^2–^. Eine konzentrations- und zeitabhängige Clusterbildung, insbesondere in der Nähe des Sättigungspunktes, ist möglich. Al^3+^ ist in Lösung von 6 Wassermolekülen umgeben. [Al(H_2_O)_6_]^3+^ unterliegt der weiteren Hydrolyse, bei der durch schrittweise Deprotonierung der koordinierten Wasserliganden gebundene Hydroxidliganden entstehen (z. B. [Al(H_2_O)_5_(OH)]^2+^, [Al(H_2_O)_4_(OH)_2_]^+^) (siehe oben) (ECHA 2019); **Aluminiumsulfat**: In Wasser entstehen bei pH < 3 vor allem [Al(OH)_n_(H_2_O)_6−n_]^(3−n)+^ (n = 0–2), [Al(H_2_O)_5_SO_4_]^+^ und [Al_2_(OH)_2_(H_2_O)_8_]^4+^, wohingegen dimeres [Al_2_(OH)_2_(H_2_O)_8_]^4+^ der vorwiegende Zustand bei pH-Werten von 3–6 ist. Bei höheren pH-Werten treten oligomere Spezies auf (Dekant 2019); **Aluminiumammoniumdisulfat, -citrat, -diacetat, -lactat, -nitrat**: k. A.; Citrat bildet mit Aluminiumionen einen Komplex (Priest 2004)
Verwendung	**Aluminiumammoniumdisulfat, Aluminiumchlorhydrat, Aluminiumchlorid, Aluminiumcitrat, Aluminiumkaliumdisulfat, Aluminiumlactat, Aluminiumsulfat**: Kosmetika (z. B. Antitranspirantien) (SCCS 2021); **Aluminiumchlorid und -sulfat**: Trink- und Abwasseraufbereitung (Entfernung von Schwebstoffen und Bakterien), Zellstoff- und Papierindustrie, Arzneimittel, Gesundheitsprodukte und Kosmetika (Health Canada 2020); **Aluminiumdiacetat, Aluminiumkaliumsulfat**: Arzneimittel (Rote Liste® Service GmbH 2024); **Aluminiumsulfat, Aluminiumkaliumdisulfat**: Lebensmittelzusatzstoff (EFSA ANS et al. 2018); **Aluminiumnitrat**: Düngemittel, chemisches Reagenz (Health Canada 2020)

Zitierte unveröffentlichte toxikologische Studien von Firmen wurden der Kommission zur Verfügung gestellt.

Lösliche Aluminiumverbindungen können mit dem von der Kommission publizierten Verfahren (Rosenberger et al. 2014) als Aluminium in der Luft bestimmt werden. Somit ist eine Abgrenzung der hier behandelten „löslichen" von den „schwerlöslichen“ Aluminiumverbindungen (Hartwig und MAK Commission 2025) möglich. Eine neue Analyse-Methode ist derzeit in Arbeit.

Die Referenzwerte des Umweltbundesamts für die Allgemeinbevölkerung liegen bei < 5 µg Al/l Blutserum und bei < 15 µg Al/l Urin (HBM-Kommission 1998). Von der Kommission wurde ein Biologischer Arbeitsstoff-Referenzwert (BAR) von 15 µg Al/g Kreatinin im Urin (Klotz et al. 2019) festgesetzt. Die geschätzte tägliche Aufnahme des Menschen über die Nahrung liegt bei 1,6 bis 13 mg (EFSA 2008).

Aluminiumphosphid ist in dieser Begründung nicht eingeschlossen, da es beim Kontakt mit Wasser selbstentzündlichen Phosphorwasserstoff bildet und dessen Toxizität führend ist (ECHA 2008). Ebenso sind Aluminium-Fluor-Verbindungen nicht Bestandteil dieser Begründung, da auch hier die Toxizität nicht primär auf Aluminium zurückzuführen ist (EU 2008).

In Tabelle 1 sind die physikalisch-chemischen Daten der in dieser Begründung behandelten löslichen Aluminiumverbindungen aufgelistet.

**Tab. 1:** Physikalisch-chemische Parameter der in dieser MAK-Begründung bewerteten löslichen Aluminiumverbindungen

**Name (Synonym)** **CAS-Nr.** **IUPAC-Name**	**Formel,** **Summenformel**	**Molmasse** **[g/mol]**	**Schmelzpunkt**	**Siedepunkt **	**Dichte** **[g/cm^3^]**	**Dampfdruck** **bei 20 °C** **[hPa]**	**Wasserlöslichkeit** **bei 20 °C [g/l]**	**pKs-Wert ** **bei 25 °C**	**Literatur**
Aluminiumammoniumdisulfat (Ammoniumalaun, Ammonium Alum, E523) [7784-25-0 (wasserfrei), 7784-26-1 (Dodecahydrat)] IUPAC: Aluminium;azanium;disulfat	AlNH_4_(SO_4_)_2_ H_4_AlNO_8_S_2_	237,15	94,5	zersetzt sich ohne Sieden ab 280 °C	1,65	k. A.	107–142	k. A.	ECHA 2020 a
Aluminiumchlorhydrat (ACH) IUPAC: Aluminium;Chlorid;Hydroxid;Hydrat	Al(OH)_x_(Cl)_3–x_^a)^ (x = 2,3–2,6; üblich: 2,5)								ECHA 2022 c
Üblich als: Dialuminiumchloridpentahydroxid [12042-91-0] IUPAC: Dialuminiumchloridpentahydroxid	Al_2_(OH)_5_Cl H_5_Al_2_ClO_5_	174,45	zersetzt sich ohne Schmelzen bis 400 °C	zersetzt sich ohne Sieden bis 400 °C		1,947	> 1000 (pH 3,3)	k. A	
Aluminiumchlorid [7446-70-0 (wasserfrei), 7784-13-6 (Hexahydrat)] IUPAC: Trichloraluman	AlCl_3_ AlCl_3_	133,34	190 °C (bei 2500 hPa)	181,2 °C	2,44	0,00003	k. A.	k. A.	ECHA 2022 a
Aluminiumchlorid, basisch [1327-41-9]	Al(OH)_x_(Cl)_3–x_^a)^(x = 0–2,3; üblich: 0,5) Al(OH)Cl_2_ HAlCl_2_O	114,89	< –90 °C	75–135 °C	1,36	k. A.	> 1000 (pH 2,4)	k. A.	ECHA 2023 a
Aluminiumchloridhydroxysulfat [39290-78-3] IUPAC: Dialuminiumdichloriddihydroxidsulfat	Al_2_Cl_2_(OH)_2_SO_4_ H_2_Al_2_Cl_2_O_6_S	175,51	–26 °C (bei 1024 hPa)	105 °C (bei 1024 hPa)	1,2	0,001 (ber.)	> 1000 (pH 3,4)	ca. 8,4 (ber.)	ECHA 2020 b
Aluminiumcitrat [31142-56-0] IUPAC: Aluminium;2-Hydroxypropan-1,2,3-tricarboxylat	Al[O_2_C-C(CO_2_)OH–CO_2_]_3_ C_6_H_5_AlO_7_	216,08	k. A.	k. A.	k. A.	k. A.	löslich	k. A.	EFSA 2008
Aluminiumdiacetat [142-03-0 (wasserfrei), 80164-67-6 (Monohydrat)] IUPAC: Aluminum;Diacetat;Hydroxid	Al(CH_3_COO)_2_(OH) C_4_H_7_AlO_5_	162,08	zersetzt sich ohne Schmelzen bis 440 °C	zersetzt sich ohne Sieden bis 440 °C	1,43	9 x 10^-8 ^(ber.)	> 0,1 (pH 4,7)	k. A.	ECHA 2020 c
Aluminiumgluconat [60007-93-4] IUPAC: Aluminium;(2*R*,3*S*,4*R*,5*R*)-2,3,4,5,6-pentahydroxyhexanoat	Al[O_2_C–(CH(OH))_4_–CH_2_OH]_3_ C_18_H_33_AlO_21_	612,4	k. A.	k. A.	k. A.	k. A.	k. A.	k. A.	NCBI 2024 a
Aluminiumkaliumdisulfat (Kaliumalaun, E522) [10043-67-1 (wasserfrei), 778-24-9 (Dodecahydrat)] IUPAC: Aluminium;Kalium;Disulfat	AlK(SO_4_)_2_ AlKO_8_S_2_	258,21	92,5 °C	zersetzt sich ohne Sieden (k. w. A.)	1,75	k. A.	59 (pH 3–3,5)	k. A.	ECHA 2019
Aluminiumlactat [18917-91-4] IUPAC: Bis(2-hydroxypropanoyloxy)alumanyl-2-hydroxypropanoat	Al[O_2_C–CH(CH_3_)–OH]_3_ C_9_H_15_AlO_9_	294,19	> 300 °C	k. A	1,388	k. A	206 (pH 2,9)	5,0	ECHA 2022 b
Aluminiummaltolat [103616-17-7] IUPAC: Aluminium;2-Methyl-4-oxopyran-3-olat	Strukturformel von Aluminiummaltolat. 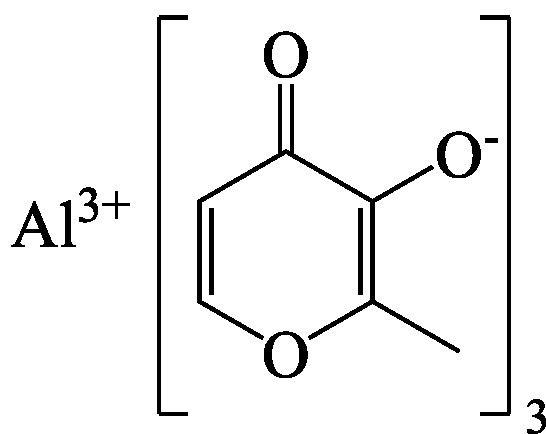 C_18_H_15_AlO_9_	402,3	k. A.	k. A.	k. A.	k. A.	k. A.	k. A.	NCBI 2024 b
Aluminiumnitrat [13473-90-0] IUPAC: Aluminium;Trinitrat	Al(NO_3_)_3_ AlN_3_O_9_	213,00	73 °C	135 °C	1,72	1 × 10^-7^	41,9 (20 °C)	k. A.	ECHA 2023 b
Aluminiumsulfat (E520) [10043-01-3 (wasserfrei), 7784-31-8 (Octadecahydrat)] IUPAC: Dialuminium;Trisulfat	Al_2_(SO_4_)_3_ Al_2_O_12_S_3_	342,13	> 300 °C	k. A	1,61–2,71	k. A	> 100	k. A.	ECHA 2023 c

^a)^ Stoffgemisch

ber.: berechnet; k. A.: keine Angabe

## 1 Allgemeiner Wirkungscharakter

Studien zur Augenreizwirkung und zur wiederholten Inhalation am Nager zeigen deutliche Unterschiede in der lokalen Wirkung der verschiedenen löslichen Aluminiumverbindungen. Das am Auge allenfalls schwach reizende Aluminiumchlorhydrat verursacht bei Ratten und Meerschweinchen nach sechs Monaten inhalativer Exposition bei 0,08 mg Al/m^3^ geringe Reizeffekte an der Lunge. Hingegen ist das am Auge als ätzend bewertete Aluminiumchlorid nach 13-wöchiger Inhalation bei 0,02 mg Al/m^3^ deutlich reizend an der Lunge von Ratten.

Eine neurotoxische Wirkung beim Menschen nach inhalativer Exposition schwerlöslicher Aluminiumverbindungen ist nachgewiesen. Mit löslichen Aluminiumverbindungen liegen hierzu keine Daten am Menschen oder am Tier nach Inhalation vor. Da aber die neurotoxische Wirkung auf gelösten Aluminiumionen beruht, ist sie auch für lösliche Aluminiumverbindungen anzunehmen.

Trotz breiter Anwendung liegen nur wenige Hinweise auf eine kontaktallergene Wirkung von löslichen Aluminiumverbindungen vor. Zur atemwegssensibilisierenden Wirkung gibt es keine eindeutigen Befunde.

Lösliche Aluminiumverbindungen sind an Bakterien und Säugetierzellen nicht mutagen. In validen In-vivo-Studien werden unterhalb zytotoxischer Konzentrationen keine Mikronuklei oder Chromosomenaberrationen induziert. Klastogene und aneugene Wirkungen sowie positive Indikatortests zeigen sich in vitro und in vivo vorwiegend bei gleichzeitiger Zytotoxizität. Aus den Daten am Menschen kann kein Zusammenhang zwischen der Entstehung von Brustkrebs und der Verwendung aluminiumhaltiger Antitranspirantien abgeleitet werden. Valide Langzeituntersuchungen zur kanzerogenen Wirkung an Tieren liegen nicht vor.

Entwicklungstoxische Effekte nach Schlundsondengabe löslicher Aluminiumverbindungen treten bei Ratten ab der niedrigsten Dosis von 13 mg Al/kg KG und Tag (als Aluminiumnitrat) und bei Mäusen ab 40 mg Al/kg KG und Tag (als Aluminiumchlorid) auf. Es kommt zu verringerten Fetengewichten und verzögerter Ossifikation bei gleichzeitiger Maternaltoxizität in Form verringerter Körpergewichtszunahme. Bei Mäusen treten zusätzlich dorsale Hyperkyphose und/oder Gaumenspalten auf. Entwicklungsneurotoxizität in Form von neuromuskulären Effekten, erniedrigter Griffstärke sowie Beeinträchtigung der Fußspreizung zeigt sich in einer Trinkwasserstudie an Ratten bei den Nachkommen ab 102 mg Al/kg KG und Tag (als Aluminiumcitrat). Es liegen keine Studien zur Entwicklungstoxizität nach inhalativer Exposition vor.

## 2 Wirkungsmechanismus

Das wirksame Agens löslicher Aluminiumverbindungen ist das Aluminiumion (Kawahara und Kato-Negishi 2011). In wässriger Lösung entstehen Komplexe, durch welche in Abhängigkeit vom pH-Wert des umgebenden Milieus H^+^-Ionen freigesetzt werden.

Aluminiumionen liegen nur in der Oxidationsstufe Al^3+^ vor, besitzen somit eine hohe Affinität für negativ geladene Sauerstoffliganden und gehen mit anorganischen und organischen Phosphaten, Carboxylaten und deprotonierten Hydroxylgruppen starke Bindungen ein. So bindet das Aluminiumion u. a. an Phosphatgruppen der DNA, RNA und Nukleosiddi- und -triphosphate. Durch die stark positive Ladung und dem im Vergleich mit anderen Metallionen wie Ca^2+^, Zn^2+^ und Na^+^ relativ kleinen Ionenradius, kann es an metallbindende Aminosäuren wie Histidin, Tyrosin, Arginin und phosphorylierte Aminosäuren binden und diese quervernetzen. Es gibt Hinweise auf eine Interaktion von Aluminium mit über 200 biologisch wichtigen Prozessen (Kawahara und Kato-Negishi 2011).

Als oxidativer Wirkungsmechanismus wird die Bildung eines Komplexes von dreiwertigem Aluminium mit dem Superoxidanion (O_2_^–^) diskutiert, welcher stärker oxidativ ist als das Superoxidanion selbst (Kong et al. 1992; Oteiza et al. 2004).

### 2.1 Lokale Reizwirkung

Die Studien zur Augenreizwirkung und zur wiederholten Inhalation am Tier zeigen deutliche Unterschiede in der lokalen Wirkung der verschiedenen löslichen Aluminiumverbindungen. Die LOAEC nach wiederholter inhalativer Exposition sowie die Einstufungen zur Augenreizung anhand der CLP (Classification, Labelling and Packaging)-Verordnung (Europäisches Parlament und Europäischer Rat 2008) sind in Tabelle 2 dargestellt.

Während Aluminiumchlorid als ätzend am Auge bewertet wurde, zeigt Aluminiumchlorhydrat allenfalls eine minimale Reizwirkung am Auge (Abschnitt 5.3.2) und wurde als nicht reizend bewertet. Dies spiegelt sich in den Untersuchungen mit wiederholter inhalativer Exposition von Ratten wider (Abschnitt 5.2.1) und fließt in die MAK-Wert-Ableitung (Abschnitt 6) ein.

**Tab. 2:** Lokale Reizwirkung der löslichen Aluminiumverbindungen

**Substanz**	**Reizwirkung am Auge, CLP-Einstufung**	**LOAEC nach wiederholter Inhalation**
Aluminiumchlorid	Kategorie 1, irreversible Wirkungen am Auge (ECHA 2022 a)	13-wöchige Inhalationsstudie (OECD TG 413), Wistar-Ratten, LOAEC 0,1 mg AlCl_3_/m^3^ (0,02 mg Al/m^3^) (BASF SE 2019 a)
Aluminiumchlorid, basisch	Kategorie 1, irreversible Wirkungen am Auge (ECHA 2023 a)	keine Daten vorhanden
Aluminiumchloridhydroxysulfat	Kategorie 2, augenreizend (ECHA 2020 b)	keine Daten vorhanden
Aluminiumcitrat	Kategorie 2, augenreizend (ECHA 2023 d)	keine Daten vorhanden
Aluminiumdiacetat	Kategorie 1, irreversible Wirkungen am Auge (ECHA 2020 c)	keine Daten vorhanden
Aluminiumlactat	Kategorie 2, augenreizend (ECHA 2022 b)	keine Daten vorhanden
Aluminiumnitrat	Kategorie 1, irreversible Wirkungen am Auge (ECHA 2023 b)	keine Daten vorhanden
Aluminiumsulfat	Kategorie 1, irreversible Wirkungen am Auge (ECHA 2023 c)	keine Daten vorhanden
Aluminiumammoniumdisulfat	keine Einstufung (ECHA 2020 a)	keine Daten vorhanden
Aluminiumchlorhydrat	keine Einstufung (ECHA 2022 c)	6-monatige Inhalationsstudien mit Aluminiumchlorhydrat, F344-Ratten u. Hartley-Meerschweinchen, LOAEC 0,25 mg/m^3^ (0,08 mg Al/m^3^) (Steinhagen et al. 1978)
Aluminiumkaliumdisulfat	keine Einstufung (ECHA 2020 b)	keine Daten vorhanden
Aluminiumgluconat, Aluminiummaltolat	keine Daten vorhanden	keine Daten vorhanden

In der 13-wöchigen Inhalationsstudie an Ratten führte die lokale Reizwirkung zu einer Schädigung der Atemwege. Schon ab der niedrigsten Konzentration von 0,02 mg Al/m^3^ trat eine Zunahme der alkalischen Phosphatase (ALP) in der bronchoalveolären Lavage-Flüssigkeit (BALF) (BASF SE 2019 a) auf, was als Zeichen von geschädigten Pneumozyten Typ II gilt (Schildge et al. 2000). Ein weiterer Hinweis auf Zellschädigung sind die erhöhten Lactatdehydrogenase (LDH)-Werte in der BALF ab 0,1 mg Al/m^3^ und das Auftreten von Erosionen und Ulzera in der Nase bei 0,4 mg Al/m^3^ (BASF SE 2019 a).

Hierfür könnte zum einen das Aluminiumion selbst, welches wie oben beschrieben sehr reaktiv ist und auch reaktive Sauerstoffspezies (ROS) induziert, verantwortlich sein, zum anderen kommt die Säurewirkung hinzu: Das in wässriger Lösung, wie sie auch an der Lungenoberfläche anzunehmen ist, entstehende Hexaaquaaluminiumion hat einen pKs-Wert von 4,96 (Hollemann und Wiberg 1976). In der Inhalationsstudie mit Ratten lag Aluminiumchlorid als 0,02%iges Aerosol mit einem pH-Wert von ca. 4 vor (BASF SE 2019 a). Da der Stoff sehr hygroskopisch ist und die Aerosolerzeugung in der Inhalationsstudie mit Schwierigkeiten behaftet war (BASF SE 2018), war möglicherweise auch eine inhomogene Verteilung im Aerosol für die, bei der eingesetzten niedrigen Konzentration und Verdünnung des Aerosols, verglichen mit einer starken Säure wie der Schwefelsäure sehr starken beobachteten Effekte verantwortlich.

### 2.2 Neurotoxizität

Bei Arbeitern, die gegen schwerlösliche Aluminiumverbindungen exponiert waren, treten präklinische neurotoxische Effekte auf (Hartwig und MAK Commission 2025; Klotz et al. 2018). Mit löslichen Aluminiumverbindungen liegen keine epidemiologischen Studien vor, jedoch muss aufgrund der besseren Bioverfügbarkeit ebenfalls mit einer neurotoxischen Wirkung gerechnet werden.

Es liegen zahlreiche mechanistische Untersuchungen zur neurotoxischen Wirkung vor, die im Folgenden detailliert beschrieben sind.

#### 2.2.1 Mitochondriale Dysfunktion und oxidativer Stress

Das Aluminiumion induziert mitochondriale Dysfunktionen, die zu einem veränderten Energiestoffwechsel, oxidativem Stress und Apoptose führen können (Skalny et al. 2021). Ein verminderter mitochondrialer Elektronentransport zeigte sich, nachdem Ratten acht Wochen lang 100 mg Aluminiumchlorid/kg KG und Tag mit der Schlundsonde erhielten. In Großhirnrinde, Mittelhirn und Kleinhirn waren die Aktivitäten der NADH-Dehydrogenase, Succinatdehydrogenase und Cytochromoxidase vermindert (Sood et al. 2011). Die 12-wöchige intragastrale Gabe von 10 mg Aluminiumlactat/kg KG und Tag an Wistar-Ratten ergab eine signifikante Abnahme der Aktivität der NADH-Cytochrom-c-Reduktase, Succinatdehydrogenase und Cytochromoxidase, begleitet von einer Reduktion der Gehalte an Cytochrom a, b, c, c1 sowie der ATP-Synthese und der ATP-Konzentration in verschiedenen Gehirnregionen. Es kam zu einer Schädigung der mitochondrialen Funktion mit einem signifikanten Anstieg an ROS im Hippocampus und Corpus striatum sowie einer signifikanten Abnahme an Glutathion (GSH) und der mitochondrialen Superoxiddismutase (SOD) (Kumar et al. 2008).

Neuronaler oxidativer Stress ist einer der wesentlichen Faktoren beim Fortschreiten einiger neurodegenerativer Krankheiten (Dey und Singh 2022). Eine Vielzahl von Studien deuten auf oxidativen Stress im Gehirn durch lösliche Aluminiumverbindungen hin (Tabelle 3). Hierbei führen lösliche Aluminiumverbindungen zu einer Zunahme an Markern für Lipidperoxidation wie Malondialdehyd (MDA), Thiobarbitursäure-reaktiven Substanzen und dem Carbonyl-Gehalt in Proteinen sowie 4-Hydroxydesoxyguanosin. Zudem wurde eine Modulation verschiedener antioxidativer Stoffe und Enzyme wie GSH, SOD, Glutathionperoxidase und eine Generierung freier Radikale wie O_2_^–•^ im Gehirn von Ratten und Mäusen festgestellt.

**Tab. 3:** Oxidativer Stress in zerebralen Zellen/Geweben durch lösliche Aluminiumverbindungen

**Substanz,** **Exposition**	**Effekt **	**Literatur**
**In vitro**		
Aluminiumsulfat, 500 µM, Rattengliom-(C-6)-Zellen, 48 h	ROS-Konz. ↑ (DCF-Assay), GSH-Gehalt ↓	Campbell et al. 1999
**In vivo**		
Aluminiumchlorid, 740, 1960, 3700 mg/kg KG, Mongolische Rennratte, oral, 2, 6, 24 h, 21 d, Gehirn	AChE-Aktivität ↓, SOD-Aktivität ↑, MDA-Gehalt ↑, O_2_^–^-Konz. ↑, SH-Gehalt ↓	Vučetić-Arsić et al. 2013
Aluminiumchlorid, 281,4 mg/kg KG und Tag, Wistar-Ratte, oral, 4 Wo, Gehirn	SOD-Aktivität ↓, GSH-Px-Aktivität ↓, ATPase-Aktivität ↓, MDA-Gehalt ↑	Feng et al. 2016
Aluminiumchlorid, 50 mg/kg KG und Tag, Wistar-Ratte, oral, 5 Wo, Gehirn	TBARS-Konz. ↑, Carbonyl-Gehalt in Proteinen ↑, SOD-Aktivität ↓	Jyoti und Sharma 2006
Aluminiumchlorid, 100 mg/kg KG und Tag, Wistar-Ratte, oral, 42 d, Gehirn	GSH ↓	Rather et al. 2019
Aluminiumchlorid, 10, 50, 300 mg/kg KG und Tag, ICR-Maus, oral, 100 d, Gehirn	SOD-Aktivität ↓, MDA-Gehalt ↑	Rui und Yongjian 2010
Aluminiumlactat, 10 mg/kg KG und Tag, Wistar-Ratte, oral, 12 Wo, Gehirn	8-OHdG-Gehalt ↑, p53-mRNA und Protein-Expression ↑, Cyclin D1-Protein-Expression ↑	Kumar et al. 2009

AChE: Acetylcholinesterase; ATPase: Adenosintriphosphatase; d: Tage; DCF: 2,7-Dichlorfluorescein; GSH: Glutathion; GSH-Px: Glutathionperoxidase; h: Stunden; MDA: Malondialdehyd; 8-OHdG: 8-Oxo-2′-desoxyguanosin; ROS: reaktive Sauerstoffspezies; SH: Thiolgruppen; SOD: Superoxiddismutase; TBARS: Thiobarbitursäure-reaktive Substanzen; Wo: Wochen

#### 2.2.2 Apoptose und Störung der Calciumhomöostase

An Swiss-Albino-Mäusen, die drei Wochen lang mit 2 mg Aluminiumchlorid/kg KG und Tag oral dosiert wurden, zeigte sich mittels Gelelektrophorese im Gehirn, aber auch der Leber, fragmentierte DNA, was ein Hinweis auf apoptotischen Zelltod ist. Zudem waren die Gehalte an anti-apoptotischem B-Zell-Lymphom (BCL2)- und Phosphoinositid-3-Kinase (PI3K)-Protein vermindert, die an P53 sowie an phosphorylierten C-Jun-N-terminale Kinasen (pJNK), Bcl2-associated-X-Protein (BAX), Caspase-3, P21- und Fas cell surface death receptor (FAS)-Protein im Cortex und im Hippocampus erhöht (Keshava et al. 2019).

Die Neuroblastomzelllinie Neuro-2a wurde 24 Stunden lang mit Aluminiummaltolat inkubiert. Hinweise auf eine induzierte Apoptose waren eine Aktivierung von Caspase-3, eine Externalisierung von Phosphatidylserin, gefolgt von nukleärer Kondensation und Fragmentierung. Die Genexpression von *Bcl2* war vermindert und die von *Bax* induziert (Johnson et al. 2005). Weiterhin scheint die Caspase-3-Aktivierung über den Fas/Fas-Ligand (FasL)-Signalweg sowie eine Aluminiumionen-induzierte Aktivierung von Daxx (death domain associated protein), welches über die Jun N-terminale Kinase (JNK) eine Stimulation von Bax induzieren kann, möglich (Skalny et al. 2021). Für die Apoptose-Induktion durch das Aluminiumion spricht auch eine Apoptosemessung mittels Annexin V-Färbung in humanen Lymphozyten nach Inkubation mit Aluminiumchlorid in vitro (siehe Abschnitt 5.6).

Eine Störung des Natrium-/Calcium-Austausches findet durch die sehr geringe Liganden-Austausch-Rate des Aluminiumions im Vergleich zu anderen Metallen statt. Beispielsweise ist die Liganden-Austausch-Rate für das Aluminiumion 10^8^-fach langsamer im Vergleich zu Ca^2+^ (Kawahara und Kato-Negishi 2011). Einer gestörten Calciumhomöostase folgend steigt der Calciumgehalt in den Mitochondrien, wodurch Cytochrom c freigesetzt, Bcl2 in den Mitochondrien vermindert, Caspasen aktiviert und DNA fragmentiert wurde (Ghribi et al. 2001; Savory et al. 2003).

Aluminiumionen beeinträchtigten die Mitochondrien und das Endoplasmatische Retikulum (ER) von Kaninchen, was die Einleitung von Apoptose zur Folge hatte. Es zeigte sich eine Zunahme von BAX und Abnahme von BCL2 im ER sowie eine Aktivierung der Caspase-12 sowie nachgeschaltet der Caspase-3. Zudem wurden die Transkriptionsfaktoren GADD 153 (Growth arrest and DNA damage-inducible protein 153) und NF-κB (nukleärer Faktor kappa-B) aktiviert, welche wiederum nach Translokation in den Zellkern Apoptose initiieren (Savory et al. 2003).

Aluminium führt zu einer verzögerten Schließung der spannungsabhängigen Calciumkanäle und einer Blockierung der Calmodulin (CaM)-abhängigen Ca^2+^/Mg^2+^-ATPase, wodurch der Schutz gegen überschüssiges intrazelluläres Calcium und Exzitotoxizität vermindert wird (Dey und Singh 2022). Eine vierwöchige i.p. (intraperitoneale) Gabe von 10 mg Aluminiumlactat/kg KG und Tag an Wistar-Ratten führte im Gehirn zu einer Verminderung der Enzymaktivität der Ca^2+^-ATPase, von CaM und der Proteinkinase C und einem Anstieg der Ca^2+^-Konzentration. Die Autoren diskutieren eine durch den erhöhten Calciumspiegel ausgelöste Induktion freier Radikale, da die vierwöchige Aluminiumexposition von Ratten im Gehirn ebenso eine Erhöhung von MDA, als Marker der Lipidperoxidation, sowie der Fluoreszenzpolarisation, welche invers proportional zur Membranfluidität ist, erkennen ließ (Julka und Gill 1996). Die durch intraneuronales Aluminium hervorgerufenen Veränderungen im Zusammenhang mit der Calciumhomöostase können in vier Bereiche eingeteilt werden: (1) Erhöhung der Ca^2+^-Ruhe- und -Spitzen-Gehalte im neuronalen Zytoplasma, (2) verminderter Ca^2+^-Einstrom in neuronale Zellen, (3) Inhibierung der Phosphatidylinositol-4,5-bisphosphat-Hydrolyse mit der Folge einer verringerten Verfügbarkeit an Inositoltrisphosphat für die nachgeschaltete Signaltransduktion, (4) Aktivierung der Proteinkinase C sowie eine geringeren Rate des Ca^2+^-Abtransports aus dem Zytoplasma. Diese Veränderungen sind ähnlich denen, die mit zunehmendem Alter und bei der Alzheimer-Krankheit beobachtet werden (Walton 2012).

#### 2.2.3 Entzündungsreaktionen

Die durch lösliche Aluminiumverbindungen ausgelöste Generierung von oxidativem Stress und freien Radikalen kann pro-inflammatorische Reaktionen hervorrufen. C57BL/6J-Mäuse, welchen 100 mg Aluminiumsulfat/kg Futter sowie 2 mg Aluminiumsulfat/l Trinkwasser ein, drei oder fünf Monate lang ad libitum verabreicht wurde, wiesen einen zeitabhängigen Anstieg pro-inflammatorischer Cytokine und Entzündungsmarker wie C-reaktives Protein, Interleukin (IL)-6 und Tumornekrosefaktor (TNF)α sowie pro-inflammatorischer microRNAs (miRNA-9, miRNA-125b und miRNA-126a) im Blutserum auf. Die Autoren sahen dies als Zeichen einer progressiven chronischen Entzündung (Pogue et al. 2017). Bestätigend hierzu stieg mRNA von *Tnf-α* im Cerebrum dosisabhängig an, nachdem Mäuse einen Monat lang Aluminiumammoniumsulfat mit dem Trinkwasser erhielten (Tsunoda und Sharma 1999). Eine sechstägige Inkubation der humanen Gliomzelllinie T98G mit Aluminiumsulfat ergab eine Aktivierung von NF-κB sowie des Cytokins TNFα. Eine erhöhte Expression von pro-inflammatorischen Cytokinen wird mit der Induktion des NF-κB in Verbindung gebracht. NF-κB ist der Schlüsselregulator von Entzündungsreaktionen, welcher die Genexpression verschiedenster zellulärer Prozesse wie Immunantwort, Zellproliferation, Entzündungen, Apoptose und Stress-Antwort modulieren kann (Campbell et al. 2002).

Als weiterer Hinweis einer neuroinflammatorischen Aktivität von löslichen Aluminiumverbindungen ist die Aktivierung der Cyclooxygenasen (COX)-1 und COX-2 zu nennen. Die 30-tägige Trinkwassergabe von 250 mg Aluminiumchlorid/kg KG an BALB/c-Mäuse führte zu einer signifikanten Zunahme der Proteinexpression von COX-1 und COX-2 im Cortex und Hippocampus. Weiterhin zeigte sich im 2,2-Diphenyl-1-picrylhydrazyl (DPPH)-Assay eine Zunahme an freien Radikalen im Cortex und Hippocampus. COX-1 wird vor allem in den Mikroglia exprimiert, wobei eine Mikroglia-Aktivierung als Folge einer Aluminiumexposition beschrieben ist. Eine erhöhte Aktivität von COX-1 wird als Ursache von oxidativem Stress einer β-Amyloid-vermittelten Neurotoxizität gesehen. COX-2, welches überwiegend in Neuronen präsent ist, ist an der Regulierung von Gehirnfunktionen, inklusive der synaptischen Plastizität beteiligt. Bei Alzheimer-Erkrankungen ist der neuronale Gehalt an COX-2 im Anfangsstadium erhöht, im fortgeschrittenen Stadium vermindert. Eine Balance zwischen COX-1 und COX-2 ist im Hinblick auf ein Gleichgewicht zwischen einer Entzündungsantwort und synaptischer Plastizität wichtig (Syed et al. 2015).

Eine vermehrte Aktivierung von Astrozyten ist ein Schlüsselereignis einer Aluminiumionen-vermittelten neuroinflammatorischen Reaktion (Dey und Singh 2022; Skalny et al. 2021). Wistar-Ratten zeigten nach einer oralen Aluminiumchlorid-Gabe (10, 100 ppm, k. w. A.) im Gehirn oxidativen Stress anhand einer erhöhten Lipidperoxidation und ROS-Bildung sowie eine dadurch vermittelte Aktivierung der Astrozyten und eine pro-inflammatorischen Reaktion durch erhöhte Expression von B-Zellen (Akinrinade et al. 2015).

#### 2.2.4 Tau-Protein, Neurofibrillenbündel und Amyloid β

Das axonale Protein Tau (tubulin associated unit; auch MAPT: microtubule associated protein tau) ist an der Stabilisierung von Mikrotubuli beteiligt. Phosphoryliertes Tau (p-Tau) ist ein zuverlässiger diagnostischer Biomarker für leichte kognitive Beeinträchtigungen (MCI, mild cognitive impairment) und ein prognostischer Biomarker für das Fortschreiten von MCI. Unter pathologischen Bedingungen kommt es zur Hyperphosphorylierung von Tau, zur Ablösung von Tau vom Mikrotubulus sowie einer Aggregation von p-Tau und der Entstehung von intrazellulären Neurofibrillenbündeln (neurofibrillary tangles, NFT). Ein charakteristisches Merkmal der Alzheimer-Pathologie ist das Vorhandensein von NFT sowie von unlöslichen Amyloidfibrillen, welche aus oligomerisiertem β-Amyloid-Protein entstehen. Die „Amyloid-Kaskaden-Hypothese“ diskutiert die β-Amyloid-Konformationsänderung und deren zentrale Rolle in der Alzheimer-Krankheit (Kawahara und Kato-Negishi 2011; Lu et al. 2014).

Das Aluminiumion sowie andere Metallionen wie Zn^2+^, Cu^2+^ und Fe^3+^ beeinflussen die Oligomerisierung und Konformationsänderungen des β-Amyloid-Proteins durch ihre vernetzende Eigenschaft, was zur Bildung nicht abbaubarer β-Amyloid-Plaques führen kann (Kawahara und Kato-Negishi 2011; Lu et al. 2014). In einer Studie mit männlichen C57BL/6-Mäusen, die zwei, vier, sechs oder zwölf Monate lang Trinkwasser mit 2,5 % Aluminiumsulfat erhielten, trat eine zeitabhängige Zunahme an β-Amyloid-Ablagerungen im Cortex des Hippocampus auf. Weiterhin zeigte sich eine Abnahme der Proteinexpression an Glucose-regulated-protein (GRP)-78, ein im ER lokalisiertes Chaperon-Protein, welches in die Entstehung der β-Amyloid-Plaques involviert ist und auch im Gehirn von an der Alzheimer-Krankheit erkrankten Personen vermindert ist (Rodella et al. 2008).

Bei Kaninchen wurden nach vierwöchiger s.c. (subkutaner) Injektion von 400 µmol Aluminiumlactat/kg KG und Tag oder einmaliger intracerebroventrikulärer Injektion von 2,5 bis 5 µmol Aluminiumlactat/kg KG Enzephalopathie und NFT in Gehirnregionen mit hohen Aluminiumkonzentrationen (v. a. kortikal) beobachtet (Forrester und Yokel 1985).

Ein Einfluss des Aluminiumions auf das Tau-Protein zeigte sich auch an gegen schwerlösliche Aluminiumverbindungen exponierten Beschäftigten einer Aluminiumgießerei, die einen signifikant erhöhten Gehalt an p-Tau 181 und p-Tau 231 im Blut aufwiesen (Hartwig und MAK Commission 2025).

#### 2.2.5 Verminderung der synaptischen Plastizität

Die Lern- und Gedächtnisfunktion resultiert aus der Interaktion und Koordination zwischen zahlreichen Neuronen im Gehirn, an der die synaptische Plastizität beteiligt ist (elektrophysiologisch ausgedrückt als Langzeitpotenzierung (LTP)). Synaptische Plastizität bedeutet, dass die Stärke der Verbindungen zwischen den Nervenzellen angepasst und die Morphologie und Funktion von Synapsen nutzungsabhängig verändert werden kann. Die Exposition gegen Aluminium kann die synaptische Plastizität und Signalweiterleitung beeinträchtigen. In einer Untersuchung erhielten 40 männliche Ratten drei Monate lang mit der Schlundsonde 500 mg Al/kg KG und Tag gelöst in Wasser (k. w. A.; vermutlich Aluminiumcitrat). Im Morris-Water-Maze-Test zeigte sich eine statistisch signifikante Zunahme der Zeit und Distanz, bis die Ratten die Plattform erreichten, was von den Autoren als Zeichen einer verminderten Lern- und Gedächtnisleistung gewertet wurden. Die Tiere wiesen, verglichen mit den Kontrolltieren, statistisch signifikant mehr Aluminium im Hippocampus und im frontalen Cortex auf. Die postsynaptische Dichte war statistisch signifikant reduziert, der synaptische Spalt vergrößert und es trat eine erhöhte Anzahl an flachen sowie perforierten Synapsen auf (Jing et al. 2004).

Acht Wochen alten Sprague-Dawley-Ratten wurden 10, 20 oder 30 µmol Aluminiummaltolat/kg KG und Tag drei Monate lang i.p. injiziert. Bei den Aluminium-exponierten Tieren zeigte sich anhand des Morris-Water-Maze-Tests eine dosisabhängige Abnahme der Lern- und Gedächtnisleistung. In den axonalen Dendriten der Neuronen der hippocampalen CA1-Region dieser Tiere nahmen die dendritischen Dornfortsätze ab und es bildeten sich fokale perlenartige Anschwellungen in Axon und Dendriten (Li et al. 2020).

Der PI3K/AKT (Phosphatidylinositol-3-kinase/Proteinkinase B)/mTOR (mechanistic Target of Rapamycin)-Signalweg spielt eine wichtige Rolle in der Instandhaltung der synaptischen Plastizität des Nervensystems. PI3K/AKT ist beteiligt an der Induktion und Aufrechterhaltung der LTP, beeinflusst die Glutamat-Rezeptor (GluR)1-Synthese und reguliert die synaptische Plastizität durch die Phosphorylierung von GluR1 an Serin 831 und Serin 845. Es zeigte sich eine statistisch signifikant verminderte Proteinexpression von AKT und mTOR im Hippocampus der exponierten Ratten (Li et al. 2020). Auch bei Beschäftigten in der Aluminiumproduktion waren im Vergleich zur Kontrolle die Serumgehalte an Aluminium statistisch signifikant erhöht und die Genexpression von *PI3KCA* (kodiert für PI3K), *AKT* und *mTOR1* statistisch signifikant vermindert (Hartwig und MAK Commission 2025).

#### 2.2.6 Störung von Neurotransmittern 

Zu den adversen neurologischen Effekten zählt zumindest teilweise die Interferenz von Aluminium mit dem Neurotransmitter-Stoffwechsel und deren Signaltransduktion. Obwohl ein spezifischer Mechanismus noch nicht bekannt ist, zeigen die existierenden Daten einen signifikanten Einfluss von Aluminium auf die Neurotransmission durch Glutamat, GABA, Acetylcholin, Serotonin und Dopamin (Skalny et al. 2021). Als Beispiel sei hier die cholinerge Funktion im Gehirn genannt, welche hauptsächlich von der Modulation des Neurotransmitters Acetylcholin (ACh) abhängt (Dey und Singh 2022). Bei männlichen Albino-Ratten, denen 25 Tage lang 140 mg Aluminiumacetat/kg KG und Tag mit der Schlundsonde verabreicht wurde, nahm die Aktivität der AChE signifikant ab, während ACh im cerebralen Cortex, Hippocampus, Hypothalamus, Cerebellum und der Pons-Medulla anstieg. Die Autoren vermuten eine Bindung des Aluminiumions an die SH-Gruppe im aktiven Zentrum der AChE (Yellamma et al. 2010).

#### 2.2.7 Fazit

Die neurotoxischen Effekte von löslichen Aluminiumverbindungen werden von einer Vielzahl an Interaktionen wie deren pro-oxidative, -inflammatorische und -apoptotische Wirkung sowie der Interferenz mit Neurotransmittern, dem Mikrotubuli-assoziierten Protein Tau, β-Amyloid-Proteinen und NFT sowie der Verminderung der synaptischen Plastizität verursacht.

### 2.3 Genotoxizität

#### 2.3.1 Oxidativer Stress

Oxidativer Stress durch lösliche Aluminiumverbindungen zeigt sich neben den Befunden im Nervensystem (Abschnitt 2.2.1) auch in weiteren Zelltypen (siehe Tabelle 4).

Die Lipidperoxidation kann durch die Bindung von Aluminiumionen an Membranen und eine dadurch veränderte Membranfluidität sowie Immobilisierung der Phospholipide begünstigt werden (Oteiza et al. 2004). Als weiterer Hinweis einer durch Aluminiumionen induzierten ROS-Bildung ist die In-vitro- und In-vivo-Induktion oxidativer DNA-Basenschäden zu nennen (siehe Abschnitt 5.6).

**Tab. 4:** Oxidativer Stress durch lösliche Aluminiumverbindungen

**Exposition**	**Effekt**	**Literatur**
**In vitro**		
Aluminiumcitrat, Nierenzelllinie, 100 µmol/l, 48 h	MDA-Gehalt ↑	Sargazi et al. 2006
Aluminiumsulfat, humane Lymphozyten, 20 µg/ml, 72 h	SOD-Aktivität ↓, G6PDH-Aktivität ↓, CAT-Aktivität ↓, GSH-Gehalt ↓	Türkez und Geyikoğlu 2011
**In vivo**		
Al (k. w. A.), 10 mM, Drosophila, Mitochondrien, Futter, 10 d	ROS-Konz. ↑, SOD-Aktivität ↑	Wu et al. 2012
Aluminiumchlorid, 281,4 mg/kg KG und Tag, Wistar-Ratte, Blut, oral, 4 Wo	SOD-Aktivität ↓, GSH-Px-Aktivität ↓, ATPase-Aktivität ↓, MDA-Gehalt ↑	Feng et al. 2016
Aluminiumchlorid 0,5 mg/kg KG und Tag, Albino-Ratte, Darm, oral, 4 Wo	SOD-Aktivität ↓, GSH-Px ↓, CAT-Aktivität ↓, MDA-Gehalt ↑, AOPP-Gehalt ↑, Carbonyl-Gehalt in Proteinen ↑, TNF-Konz. ↑	Eltahawy et al. 2017

AOPP: „advanced oxidation protein products“; CAT: Katalase; d: Tage; G6PDH: Glucose-6-phosphat-Dehydrogenase; GSH: Glutathion; GSH-Px: Glutathionperoxidase; h: Stunden; MDA: Malondialdehyd; ROS: reaktive Sauerstoffspezies; SOD: Superoxiddismutase; TNF: Tumornekrosefaktor; Wo: Wochen

#### 2.3.2 Störung der Eisenhomöostase

Aluminium konkurriert mit Eisen um Bindungsstellen in Proteinen wie Ferritin, Transferrin, IPR (Eisenregulationsprotein) oder Eisenchelatoren, wodurch der zytoplasmatische Eisenpool erhöht und das bioverfügbare Eisen in den Mitochondrien vermindert wird. Eine Störung der Eisenhomöostase kann zur Induktion der Lipidperoxidation führen (Kawahara und Kato-Negishi 2011; Yamanaka et al. 1999). In gegen Aluminium exponierten Leberzellen (HepG2) zeigte sich eine Verminderung an bioverfügbarem Eisen in den Mitochondrien, wodurch eisenabhängige Enzyme des Citratzyklus sowie der oxidativen Phosphorylierung in den Mitochondrien beeinträchtigt wurden, was zu einer metabolischen Umlagerung mit Folge einer verminderten β-Oxidation der Fettsäuren und erhöhter Peroxidation führte^.^(Mailloux et al. 2011).

#### 2.3.3 Beeinflussung der DNA-Konformation

In vitro wurde eine reversible Veränderung der DNA-Struktur durch eine vermutlich spezifische Bindung von Aluminiumionen am Sauerstoff der Phosphatgruppe der DNA sowie eine Bindung durch hydroxylierte Aluminiumverbindungen an DNA-Basen gezeigt. Zudem bewirkt Aluminium ab einer Konzentration von 0,01 mM in vitro einen Übergang von der vorliegenden B-Konformation der DNA in die Z-DNA-Form (Greim 2007). Die Bindung des Aluminiumions an Phosphatgruppen von DNA und RNA kann über die Beeinträchtigung der DNA-Topologie die Genexpression beeinflussen (Kawahara und Kato-Negishi 2011).

#### 2.3.4 Wirkung als Vernetzer

In Hepatom-Zellen treten bei 0,5 mM Aluminiumchlorid DNA-Protein-Cross-Links nach einer fünfstündigen Inkubation auf (Greim 2007). Die Bindung des Aluminiumions an phosphorylierte Aminosäuren fördert die Selbstaggregation und Akkumulation von stark phosphorylierten Zytoskelettproteinen wie Neurofilamenten und Mikrotubuli-assoziierten Proteinen (Kawahara und Kato-Negishi 2011). Die Inkubation von Rinder-, Ratten- und Kaninchen-Neurofilamenten mit Aluminiumlactat ergab ab 0,75 mM eine signifikante Aggregation (Troncoso et al. 1990). Auch bei Neurofilamentproteinen und den Mikrotubuli-assoziierten Proteinen (MAP)-1A, MAP-1B und MAP-2 von Rinderhirn zeigte sich eine in vitro durch Aluminiumchlorid induzierte Aggregation (Díaz-Nido und Avila 1990). Die Aggregation von Mikrotubuli-assoziierten Proteinen könnte als Hinweis auf eine aneugene Wirkung verstanden werden. Bestätigend hierzu traten in humanen Lymphozyten mittels FISH-Testung zentromerpositive als auch zentromernegative Mikronuklei als Hinweis auf eine klastogene und aneugene Wirkung auf (siehe Abschnitt 5.6). Die Inkubation von V79-Zellen mit 100 µM Aluminiumchlorid ergab zudem eine erhöhte Anzahl von Zellen mit multipolaren Spindeln (Tenan et al. 2021).

#### 2.3.5 Interaktion mit DNA-Reparaturgenen

Die Interaktion mit DNA-Reparaturgenen wurde durch die 19- bis 21-wöchige Exposition von immortalisierten, humanen Brustepithelzellen (MCF10A) mit Aluminiumchlorid und Aluminiumchlorhydrat in Konzentrationen von 10^–4^ M untersucht. Es zeigte sich ein verminderter Gehalt an mRNA der Reparaturgene *BRCA1*, *BRCA2*, *CHK1*, *CHK2*, *Rad51* und *ATR* und ein reduzierter Proteingehalt an BRCA1. Die Autoren diskutieren, dass die Langzeitexposition eine Reduktion der DNA-Reparatur verursachen könnte (Farasani und Darbre 2015).

#### 2.3.6 Fazit

Eine durch lösliche Aluminiumverbindungen induzierte Genotoxizität kann durch oxidativen Stress ausgelöst werden. Ursachen sind u. a. Beeinträchtigungen der Calcium- und Eisenhomöostase. Zudem können genotoxische Effekte durch eine Beeinträchtigung der DNA-Topologie und Induktion von Entzündungsreaktionen (siehe Abschnitt 3.1.1) ausgelöst werden. Es gibt Hinweise auf Aneugenität durch eine Aggregation von Mikrotubuli-assoziierten Proteinen. Diskutiert wird eine Interaktion mit DNA-Reparaturgenen und dem Superoxidanion.

### 2.4 Kanzerogenität

Aufgrund erhöhter Aluminiumkonzentrationen im Tumorgewebe der Brust wurde ein möglicher Zusammenhang zwischen der Verwendung aluminiumhaltiger Antitranspirantien und dem Auftreten von Brustkrebs vermutet (Exley et al. 2007; House et al. 2013; Linhart et al. 2017; Millos et al. 2009; Mulay et al. 1971; Ng et al. 1997; Pasha et al. 2008; Rodrigues-Peres et al. 2013; Romanowicz-Makowska et al. 2011).

Beim oberen äußeren Quadranten der Brust, der nah am Anwendungsort von Antitranspirantien ist, handelt es sich um einen Bereich mit vermehrtem Drüsengewebe. In diesem Drüsengewebe lässt sich im Vergleich zu den anderen Quadranten häufiger eine Tumorbildung beobachten (Klotz et al. 2017), was mit einer erhöhten Einlagerung von Metallen wie Aluminium einhergeht (Ng et al. 1997; Ogoshi et al. 1994). Eine Erklärung könnte sein, dass Aluminium an Transferrin, ein für den Eisentransport zuständiges Protein, bindet (Yokel und McNamara 2001). Im malignen Tumorgewebe ist bekannterweise die Metall-Homöostase stark verändert und die Transferrin-Rezeptor-Dichte deutlich erhöht (Elliott et al. 1993). So ist Aluminium vermutlich nicht der Auslöser von Tumorerkrankungen im Brustgewebe, sondern wird verstärkt ins Tumorgewebe eingelagert.

Dies bestätigt eine Untersuchung an sieben Wochen alten weiblichen Sprague-Dawley-Ratten, denen einmalig 5 mg 2,7-Dimethylbenz[a]anthracen in den Bauchraum injiziert wurde. Von 80 Ratten entwickelten 56 nach 20 Wochen Brustdrüsentumore, in denen der Aluminiumgehalt, verglichen mit dem Brustdrüsengewebe von Ratten, die keine Tumore entwickelten, statistisch signifikant erhöht war (Ogoshi et al. 1994).

### 2.5 Reproduktionstoxizität

Bei Mäusen, jedoch nicht bei Ratten, gibt es Hinweise auf Aluminium-induzierte Fertilitätsstörungen (siehe Abschnitt 5.5.1). Männliche Reproduktionsendpunkte sind stärker betroffen als weibliche. Neben DNA- und Spermatozoenschäden sowie reduzierten Blut-Testosteronspiegeln und Spermienzahlen wird oxidativer Stress (erhöhte MDA-Werte) in den Hoden beobachtet, was eine mögliche Ursache sein kann (Yokel 2020).

## 3 Toxikokinetik und Metabolismus

### 3.1 Aufnahme, Verteilung, Ausscheidung

#### 3.1.1 Aufnahme

##### 3.1.1.1 Inhalative Aufnahme

**Abb. 1:**
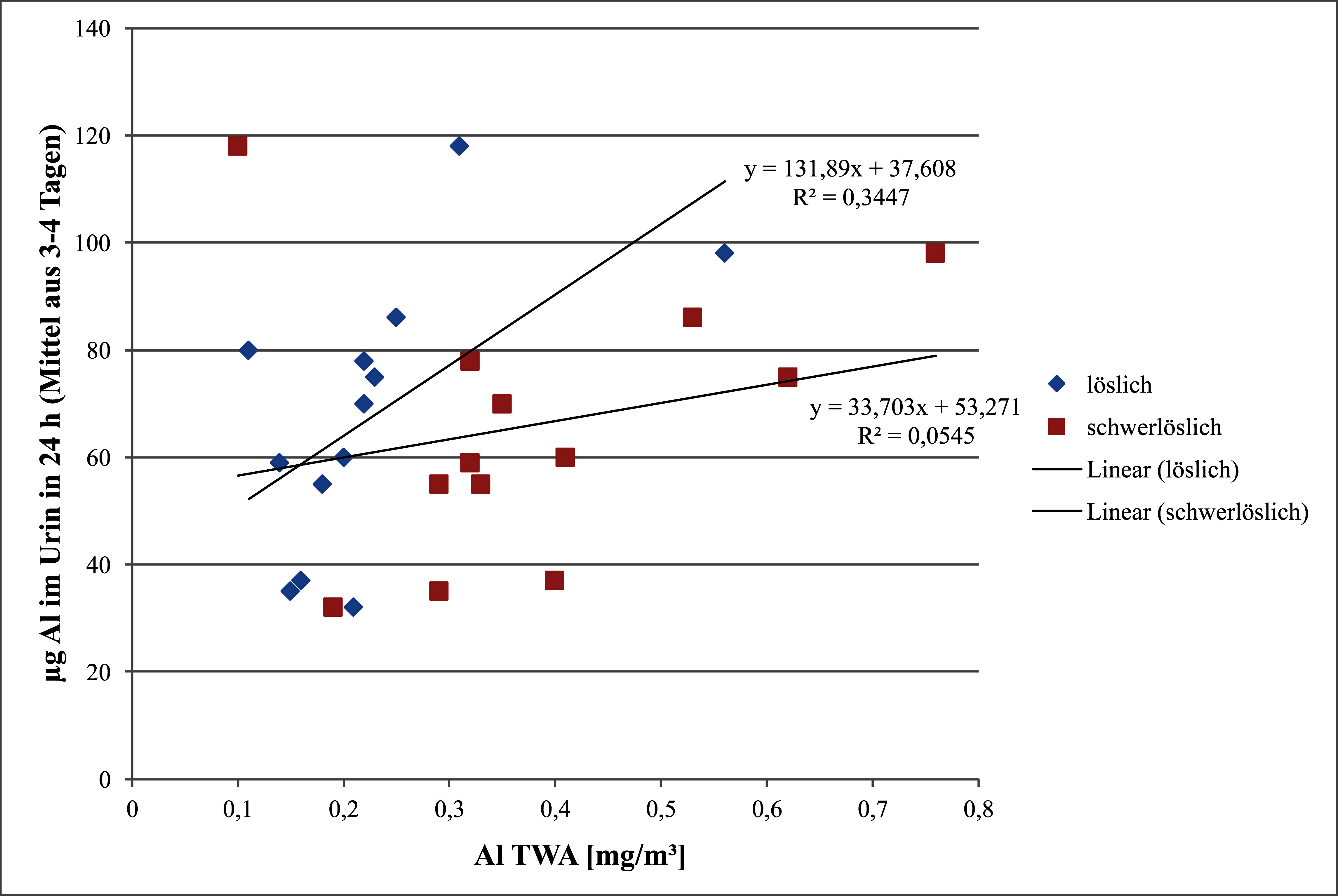
Beziehung zwischen den Konzentrationen von löslichem und schwerlöslichem Aluminium in der Luft (TWA, zeitgewichteter Mittelwert) und der Menge an Aluminium im 24-Stunden-Urin bei Arbeitern in der Studie von Pierre et al. (1995)

Bei inhalativ gegen schwer- und leichtlösliche Aluminiumverbindungen exponierten Beschäftigten wurde eine HWZ für die Elimination von Aluminium im Urin von 5–9 Stunden angegeben (Pierre et al. 1995). In Abbildung 1 sind die Daten der Arbeiter aus Betrieb B und C dargestellt. Aus der Regressionsgeradengleichung der Beziehung zwischen der Konzentration von leichtlöslichem Aluminium in der Luft und im 24-Stunden-Urin ergibt sich, dass 500 µg lösliches Al/m^3^ 102 µg Al im 24-Stunden-Urin entsprechen. Bei 10 m^3^ Atemvolumen wäre die Resorption nur 2 % und damit so hoch wie die für schwerlösliche Aluminiumverbindungen, was nicht plausibel ist. Um zu berücksichtigen, dass das im Urin ausgeschiedene Aluminium auch von schwerlöslichen Aluminiumverbindungen und von der Hintergrundbelastung mit der Nahrung stammt, aber andererseits resorbiertes Aluminium nicht vollständig mit dem Urin (nur ca. 60 %) ausgeschieden sondern auch in andere Körperkompartimente verteilt wird (Priest 2004), wird eine inhalative Resorption von 5 % angenommen.

Nach Untersuchungen an Ratten, denen verschiedene lösliche Aluminiumverbindungen in die Nasenhöhle appliziert wurden, wird eine direkte Aluminiumaufnahme über den Riechkolben zu den olfaktorischen Nerven bis hin zum Gehirn diskutiert. Es gibt jedoch keine Daten zur Bioverfügbarkeit, um zu entscheiden, ob dies ein bedeutender Aufnahmeweg ist (Yokel und McNamara 2001).

##### 3.1.1.2 Orale Aufnahme

Die orale Bioverfügbarkeit von Aluminium ist sehr gering und beträgt beim Menschen nach Aufnahme über die Nahrung etwa 0,1 %, kann aber um den Faktor 10 variieren (EFSA 2008).

Die Aluminium-Gewebe- oder -Blutkonzentrationen bei Versuchstieren nach oraler Verabreichung von Aluminiumsalzen sind uneinheitlich und unabhängig davon, welche Aluminiumsalze verabreicht wurden. Eine generelle Extrapolation von der Wasserlöslichkeit auf die Bioverfügbarkeit ist nicht möglich. So kann eine lösliche Aluminiumverbindung im Magen als Al^3+^ × 6 H_2_O vorliegen, im Fortgang der Aufnahme im Darm entsteht allerdings der weniger lösliche Hydroxykomplex. Andererseits können Aluminiumsalze mit geringer Wasserlöslichkeit im sauren Magen je nach Mageninhalt und -aktivität teilweise in besser lösliches Aluminiumchlorid umgewandelt werden (Dekant 2019).

Aus Tierversuchsdaten zeigt sich eine geringe orale Bioverfügbarkeit. In Übersichtarbeiten ist eine orale Bioverfügbarkeit von ^26^Aluminiumsalzen im Bereich von < 1 % für Ratten und Kaninchen berichtet, von Aluminiumcitrat werden um 2 % aufgenommen (ATSDR 2008; Greim 2007), was eventuell an der Komplexierung durch Citrat liegen kann (Priest 2004).

In einer Studie zur oralen Bioverfügbarkeit wurde einmalig eine hohe Dosis von je 50 mg Al/Ratte (ca. 400 mg/kg KG) als Aluminiumcitrat, Aluminiumchlorid, Aluminiumnitrat und Aluminiumsulfat, gelöst in Wasser, welches 1 % Carboxymethylcellulose enthielt, an jeweils sechs weibliche Sprague-Dawley-Ratten per Schlundsonde verabreicht. Die Verbindungen waren mit 1,24–2,44 ng ^26^Al markiert. Zusätzlichen zwölf Ratten wurde Aluminiumcitrat (0,19 ng ^26^Al) in den Blutkreislauf injiziert. Die Kontrolltiere erhielten Wasser ohne zugesetztes Aluminium. Nach sieben Tagen wurden die Tiere getötet und der Anteil von ^26^Al mittels Beschleuniger-Massenspektrometrie (AMS) bestimmt. Die Autoren geben an, dass nach dieser Zeit die Kurzzeit-Clearance beendet ist. Die orale Bioverfügbarkeit wurde durch den Vergleich des verbleibenden Anteils der ^26^Al-Menge in der Karkasse nach oraler und i.v. (intravenöser) Gabe nach sieben Tagen bestimmt und betrug 0,21 % für Aluminiumsulfat, 0,08 % für Aluminiumcitrat, 0,05 % für Aluminiumchlorid und 0,05 % für Aluminiumnitrat. Die Autoren verweisen darauf, dass die orale Bioverfügbarkeit bei der Ratte ähnlich wie beim Menschen ist (AECL 2010; Priest et al. 2021).

In einer Studie nach OECD-Prüfrichtlinie 417 (von 1984) wurde an je vier männliche und vier weibliche Wistar-Ratten 447 mg Aluminiumsulfat/kg KG (71 mg Al/kg KG), 450 mg basisches Aluminiumchlorid/kg KG (139 mg Al/kg KG) oder 833 mg Aluminiumchloridhydroxysulfat/kg KG (207 mg Al/kg KG) einmalig per Schlundsonde verabreicht. Mit Werten von < 0,5 % für alle drei Verbindungen bestätigt diese Studie die bekannte geringe orale Resorption (NOTOX B.V. 2007 b).

##### 3.1.1.3 Dermale Aufnahme

###### 3.1.1.3.1 In vitro

Drei Antitranspirant-Formulierungen (Aerosol mit 38,5 % Aluminiumchlorhydrat, Roll-on-Emulsion mit 14,5 % Aluminiumchlorhydrat, Stick mit 21,2 % Aluminiumchlorhydrat) wurden 24 Stunden lang auf exzidierte Humanvollhaut (1,76 cm^2^) in einer Franz-Diffusionszelle aufgebracht. Als Kontrolle diente physiologische Kochsalzlösung. Die applizierten Konzentrationen betrugen 248,5 µg Al/cm^2^, 164,3 µg Al/cm^2^ und 163,8 µg Al/cm^2^ für das Aerosol, die Roll-on-Emulsion bzw. den Stick. Der Stick wurde zur Simulation einer Unterarmrasur zusätzlich in einer Konzentration von 190,5 µg Al/cm^2^ auf Tape-gestrippte Haut okklusiv appliziert. Die innerhalb von 24 Stunden in Epidermis, Dermis und Rezeptorflüssigkeit penetrierte Menge betrug maximal 1,8 µg/cm^2^ für Normalhaut und 11,5 µg/cm^2^ für Tape-gestrippte Haut. In der Rezeptorflüssigkeit wurden 0,0 ± 0,01 µg/cm^2^ bis 0,10 ± 0,05 µg/cm^2^ gefunden, was sich vom Hintergrundwert (Kontrolle normale Haut: 0,008 ± 0,06 µg/cm^2^; Kontrolle Tape-gestrippte Haut: 0,09 ± 0,01 µg/cm^2^) kaum unterschied. Hauptsächlich wurde Aluminium also in der Haut gefunden (Pineau et al. 2012). Aus der Studie ergeben sich Penetrationsquoten von 1 bis 10 % bei 24-stündiger Applikation.

In einer Studie an exzidierter Schweinehaut (1 mm Stärke) wurde bei offener 24-stündiger Applikation von Aluminiumchlorhydrat-haltigen kosmetischen Formulierungen (2,5 mg Al/cm^2^) bei einer Nachweisgrenze von 0,01 µg/ml keine Penetration in das Rezeptormedium gefunden. In die Haut penetrierten 3,53 µg Al/cm^2^, was 5,7 % entsprach (ECHA 2022 c).

In einer Studie nach OECD-Prüfrichtlinie 428 wurde die dermale Penetration von Aluminiumlactat (2,1 g Aluminium/100 g) untersucht. Obwohl die mittleren Gesamtwiederfindungen unter den in den Prüfrichtlinien festgelegten Qualitätskriterien liegen, wird die Studie im Allgemeinen als valide bewertet. Der Hauptgehalt an Aluminiumlactat (gemessen als Aluminium) wurde mit 85,69 % in der Waschlösung nach acht Stunden nachgewiesen. In der Rezeptorflüssigkeit und der Waschflüssigkeit der Rezeptorkammer wurde so gut wie keine Testsubstanz gefunden. Es wurden 0,06 % des Aluminiumlactats (ausgedrückt als Aluminium) unter den verwendeten Testbedingungen über einen Behandlungszeitraum von acht Stunden durch die menschliche Haut resorbiert (ECHA 2022 b).

###### 3.1.1.3.2 In vivo

Bei 21 Freiwilligen, die 14 Tage lang ein kommerzielles Aluminiumchlorhydrat-haltiges Antitranspirant verwendeten, wurde Aluminium im Blutplasma und 24-Stunden-Urin vor und nach der Exposition gemessen. Es konnte keine statistisch signifikante Zunahme der Aluminiumkonzentrationen nachgewiesen werden. Weder Rasiergewohnheiten noch aufgetragene Menge hatten einen Einfluss. Die maximal gefundenen Konzentrationen betrugen 9,42 µg Al/g Kreatinin (n = 15) und 2,1 µg Al/l Plasma (n = 7) (Letzel et al. 2020) und die erstere liegt damit unter dem BAR (Klotz et al. 2019)

Zwei Probanden (ein Mann, eine Frau) wurde eine wässrige ^26^Al-markierte 21%ige Aluminiumchlorhydrat-Formulierung auf jeweils eine Achselhöhle okklusiv aufgetragen. Appliziert wurden ca. 13 mg Al (8 ng ^26^Al (BfR 2023)) auf 77 cm^2^ (entspricht 0,1 ng ^26^Al/cm^2^ bzw. 170 µg Al/cm^2^). In den ersten sechs Tagen wurde der okklusive Verband täglich abgenommen und die oberste Hautschicht mit einem Klebeband entfernt. Anschließend wurde die Haut gewaschen, getrocknet und erneut abgeklebt. Bei der Frau hatte das Klebeband eine leichte Reizwirkung und die abgeklebten Hautstellen um die Applikationsstelle waren ab dem zweiten Tag verletzt. Bei der Frau fanden sich 30 % der applizierten Dosis am ersten Tag in der entfernten Haut, beim Mann 45 %. Die Gesamt-Wiederfindung betrug 31 % bzw. 48 %. Urin- und Blutproben wurden 53 Tage lang genommen. Sechs Stunden bis 15 Tage nach der Applikation konnte ^26^Aluminium im Blut nachgewiesen werden. Jedoch waren die Konzentrationen im Blut zu gering, um eine verlässliche Quantifizierung zu ermöglichen. Diese erfolgte anhand der Urin-Daten. Mit dem Urin wurden vom Mann 1,1 µg Al (0,0082 % der Gesamtdosis) und von der Frau 1,9 µg Al (0,016 % der Gesamtdosis) ausgeschieden, das meiste davon innerhalb der ersten zwei Wochen. Aus einer Kinetik-Studie an Probanden mit ^26^Al, bei der 80–90 % der i.v. gegebenen Menge innerhalb von 40 Tagen mit dem Urin ausgeschieden wurde, wurde berechnet, dass 0,014 % der dermal applizierten Menge bioverfügbar waren. Hochgerechnet auf eine Applikation auf zwei Achselhöhlen entsprach dies im Durchschnitt 3,6 µg Al. Die Aluminiumaufnahme der Frau war fast doppelt so hoch wie die des Mannes (Flarend et al. 2001).

Eine mit ^26^Al markierte 25%ige Aluminiumchlorhydrat-Antitranspirant-Formulierung wurde bei zwölf Probandinnen auf jeweils beide Achselhöhlen (je ca. 100 cm^2^) aufgetragen und nicht abgedeckt. Die applizierte Gesamtmenge betrug 1,5 g (entspricht: 113 mg Al; 565 µg Al/cm^2^, 138 ng ^26^Al). Nach 24 Stunden durften sich die Probandinnen waschen. Blutproben wurden über einen Zeitraum von 28 Tagen genommen, sowie punktuell Morgenurinproben. Die ^26^Al-Gehalte lagen nur bei zwei der 504 Blutproben über der Nachweisgrenze von 0,122 fg/ml. Der Aluminiumgehalt in den Urinproben war nur an den ersten drei Tagen nach Auftragung quantifizierbar (Bestimmungsgrenze 0,061 fg/ml). Mit Hilfe von Interpolationen und Annahmen wurde eine mittlere Bioverfügbarkeit von 0,0094 % berechnet (de Ligt et al. 2018).

Die folgende Studie von TNO aus dem Jahr 2019 liegt nicht im Original vor, wurde aber vom Bundesamt für Risikobewertung (BfR 2023) ausführlich beschrieben: Sechs Probandinnen wurde 1,5 g der gleichen Formulierung wie in der Studie von de Ligt et al. (2018) appliziert, aber mit einer höheren ^26^Al-Menge von 3732 ng, einmalig auf beide Achselhöhlen (je 100 cm^2^) aufgetragen, an der Luft getrocknet und dann mit Verbandmull semi-okklusiv abgedeckt. Die Probandinnen mussten darüber ein T-Shirt tragen. Die aufgetragene Aluminium-Menge wurde über die aufgetragene Isotopenmenge mit 83 mg angegeben (entspricht 415 µg Al/cm^2^). Vor der Applikation hatten sich die Probandinnen vier Wochen lang täglich nass rasiert. Nach 24 Stunden wurde das exponierte Hautareal gewaschen und wieder semiokklusiv mit Verbandmull abgedeckt und ein T-Shirt übergezogen. Nach weiteren 24 Stunden wurde dieser Vorgang wiederholt. Die Waschlösung, verwendeten Hilfsmittel, Verband und T-Shirt wurden auf den Gehalt von ^26^Aluminium untersucht. Blutproben wurden an definierten Tagen bis zum 28. Tag nach dermaler Applikation genommen. Urin und Faeces wurden zehn Tage lang vollständig gesammelt, zudem wurde 24-Stunden-Sammelurin am 14., 21. und 28. Tag untersucht. Am 7. und 35. Tag wurde von einer Achselhöhle mit Tape-Stripping die Hornschicht gewonnen und am 35. Tag eine Stanzbiopsie von diesem von der Hornschicht befreiten Areal genommen. Den Probandinnen wurde auch einmalig eine ^26^Al-markierte Aluminiumcitrat-Lösung i.v. injiziert, um die Bioverfügbarkeit zu bestimmen. Die ^26^Al-Gehalte im Blut lagen nach dermaler Applikation lediglich bei zwölf der 84 Proben über der Nachweisgrenze (0,118 fg/ml) und waren daher für die Bioverfügbarkeitsberechnung nicht zu verwenden. Nach dermaler Gabe wurden im Mittel (n = 5) 0,00036 % und nach i.v. Gabe 70 % mit dem Urin ausgeschieden, davon 90 % innerhalb der ersten sechs Tage. Daraus wurde eine Bioverfügbarkeit von im Mittel 0,00052 % errechnet. In den Faeces wurden im Durchschnitt 0,0014 % der dermal applizierten Dosis wiedergefunden. Die Wiederfindung betrug insgesamt 70 %, davon 62 % nach 24 Stunden in der Waschlösung und 6 % im ersten getragenen T-Shirt. In einer weiteren Studie wurde daraufhin gezeigt, dass ein beträchtlicher Anteil der applizierten Formulierung auf der Hautoberfläche verblieb und mit der Zeit durch Kontakt mit dem auf dem Körper getragenen Gewebe (Verbandmull, T-Shirt) verloren ging, was die unvollständige Wiederfindung erklären kann. Diese Daten lieferten keinen Hinweis darauf, dass die Haut ein Reservoir für Aluminium bildet. Das BfR leitete aus dieser Studie eine Bioverfügbarkeit nach dermaler Applikation von 0,00192 % ab (0,00052 % Urin + 0,0014 % Faeces) (BfR 2023).

###### 3.1.1.3.3 Aufnahmeabschätzung bezogen auf beruflich verwendete Hautschutzcremes

In Hautschutzcremes beträgt die Konzentration von Aluminiumchlorhydrat 5 % (BfR 2014), was etwa 1,5 % Aluminium entspricht. Nach einer Untersuchung an Krankenschwestern werden etwa 1 mg Creme/cm^2^ als Hautschutz appliziert, im Mittel pro Tag etwa dreimal (Schliemann et al. 2012). Auf beide Hände (1000 cm^2^) appliziert, entspricht das 3000 mg, also 45 mg Aluminium (45 µg Al/cm^2^). Davon werden ca. 0,002 % resorbiert, also 0,9 µg Al/Tag.

Da der Referenzwert (BAR) der Aluminiumausscheidung 15 µg/g Kreatinin beträgt (Klotz et al. 2019) und pro Tag etwa eine Ausscheidung von im Durchschnitt 1,3 g Kreatinin zugrunde gelegt wird (Bader et al. 2020), liegt eine über die Haut aufgenommene Menge von 0,9 µg/Tag (von der aber nur ein Viertel mit dem Urin ausgeschieden wird) weit im Hintergrundbereich, und damit auch sehr weit unterhalb des BAT-Werts von 50 µg/g Kreatinin (Klotz et al. 2018). Die sehr geringe Aufnahme von Aluminium über die Haut wird auch durch die Probandenstudie mit 14-tägiger Applikation eines Aluminiumchlorhydrat-Antitranspirants (Letzel et al. 2020) bestätigt.

#### 3.1.2 Verteilung und Ausscheidung

Nach der Aufnahme verteilt sich Aluminium gleichmäßig zwischen Plasma und zellulären Blutbestandteilen. Im Plasma sind 80 bis 94 % des Aluminiums an Proteine gebunden, davon 80 % an Transferrin, 10 % an Albumin und 5 % an niedermolekulare Proteine. Bei Menschen und Tieren erfolgt die Elimination des resorbierten Aluminiums zu etwa 98 % mit dem Urin, eine Ausscheidung mit der Galle spielt eine untergeordnete Rolle. Zur biologischen HWZ der renalen Aluminiumausscheidung nach inhalativer Aufnahme gibt es stark voneinander abweichende Angaben. In Abhängigkeit von der Expositionssituation und der Expositionsdauer und der Verbindung streuen die Angaben zur HWZ von wenigen Stunden bis zu Wochen und Jahren. Neben erheblichen individuellen Unterschieden spielt bei der renalen Ausscheidungskinetik vermutlich die Aluminiumspeicherung in verschiedenen Kompartimenten des Organismus mit deren unterschiedlichen Eliminationsverhalten eine entscheidende Rolle. Die biologische HWZ der renalen Aluminiumausscheidung scheint zudem entscheidend von der kumulativen Vorexposition abzuhängen (Greim 2007).

Das Aluminiumion kann, vermutlich an Transferrin gebunden, die Blut-Hirn- sowie die Blut-Liquor-Schranke passieren und akkumuliert in der an Transferrin-Rezeptoren reichen Cortex-Region. Die Anreicherung in Gehirn und Knochen kann im Zusammenhang mit einem Mangel an Calcium und Magnesium stehen. Es ist möglich, dass die Verteilung durch Citrat oder Fluorid durch eine verminderte Gewebsanreicherung aufgrund der Sättigung der Transferrin-Bindungskapazität beeinflusst wird (Dekant 2019). Insgesamt ist etwa 1 % des gesamten Aluminiums im Gehirn (in der grauen Substanz etwa doppelt so viel wie in der weißen Substanz) zu finden, dabei ist der Aluminiumgehalt im Gehirn geringer als in allen anderen Organen (Inan-Eroglu und Ayaz 2018).

Nach sechsmonatiger inhalativer Exposition von Ratten und Meerschweinchen gegen bis zu 25 mg Aluminiumchlorhydrat/m^3^ (massenmedianer aerodynamischer Durchmesser (MMAD) ca. 1,5 µm) wurde zwar ein deutlich erhöhter Aluminiumgehalt in der Lunge (0, 0,25; 2,5; 25 mg/m^3^: 10, 20, 40, 100 µg Al/g Lunge), aber nicht in Blut, Herz, Leber, Nieren, Milz oder Gehirn nachgewiesen (Steinhagen et al. 1978). Eine Nachweisgrenze für Aluminium wurde nicht angegeben, auch wurde das wichtige Speicherorgan Knochen nicht untersucht. Der fehlende Nachweis in Blut, Herz, Leber, Nieren, Milz und Gehirn kann somit nicht zweifelsfrei auf eine fehlende Bioverfügbarkeit von Aluminiumchlorhydrat interpretiert werden.

Jeweils fünf männliche Albino-Ratten erhielten einmalig 0 oder 70 mg Aluminiumchlorid/kg KG mittels Schlundsonde. Der Aluminiumgehalt in Blut, Leber, Niere, Gehirn und Darm wurde nach 24 Stunden, 3, 7, 14 und 28 Tagen gemessen und die kinetischen Daten errechnet (Tabelle 5). In Leber, Nieren, Darm und Gehirn der dosierten Tiere war der Aluminiumgehalt verglichen mit dem der Kontrolltiere zu allen Untersuchungszeitpunkten statistisch signifikant erhöht. Im Serum war nach 28 Tagen eine statistisch signifikante Abnahme zu erkennen. Die AUC_total_ (area under concentration-time curve) war am höchsten im Gehirn, gefolgt von Nieren, Serum, Darm und Leber. Die längste Eliminationshalbwertszeit, die längste mittlere Verweildauer, die langsamste Clearance, die niedrigste Eliminationskonstante und die längste Zeit bis zum Erreichen der maximalen Konzentration zeigten sich im Gehirn. Die C_max_ von Aluminium war im Darm am höchsten, gefolgt von Nieren, Serum, Gehirn und Leber. Die Werte waren jedoch sehr ähnlich. Die berechnete Startkonzentration von Aluminium am ersten Expositionstag (C_0_) war am höchsten im Serum, gefolgt von Darm, Gehirn, Nieren und Leber (Rawy et al. 2013). Die Aufnahme in die Knochen, das Hauptkompartiment von Aluminium im Organismus, wurde nicht untersucht.

**Tab. 5:** Toxikokinetische Parameter (Mittelwerte±Standardfehler) nach einmaliger oraler Gabe von AlCl_3_ (70 mg/kg KG) an Ratten (Rawy et al. 2013)

**Organ/Matrix**	**AUC_total_**	**T_max_**	**C_max_**	**C_0_**	**Lz**	**t_1/2_**	**MRT**	**Cl**
	**[µg/g × d]**	**[d]**	**[µg/g FG]**	**[µg/g FG]**	**[d^–1^]**	**[d]**	**[d]**	**[l/d]**
Leber	10,04±0,43	3,8	1,37 ± 0,09	0,83 ± 0,05	0,03 ± 0,00	30,71 ± 7,19	45,47 ± 9,92	1,39 ± 0,18
Niere	19,04±0,78	3,8	2,48 ± 0,06	1,48 ± 0,09	0,03 ± 0,00	28,59 ± 4,08	43,33 ± 5,93	0,72 ± 0,07
Gehirn	22,48±0,42	5,4	1,88 ± 0,06	1,63 ± 0,04	0,01 ± 0,00	85,71 ± 17,40	124,22 ± 25,14	0,33 ± 0,01
Dünndarm	14,18±0,36	3,0	2,59 ± 0,09	2,07 ± 0,09	0,04 ± 0,00	16,54 ± 0,47	25,14 ± 0,74	1,15 ± 0,03
Serum	14,92±0,34	2,2	2,18 ± 0,09	2,18 ± 0,09	0,04 ± 0,00	19,08±1,38	28,52 ± 2,05	1,12 ± 0,05

AUC_total: _area under concentration-time curve; Cl: Clearance; C_max: _ maximale Konzentration; C_0:_ Konzentration am 1. Expositionstag; d: Tag; FG: Feuchtgewicht; MRT: mittlere Verweildauer (mean residence time); Lz: Eliminationsgeschwindigkeitskonstante; t_1/2_: Eliminationshalbwertszeit; T_max_: Zeit bis zum Erreichen von C_max_

Jeweils fünf Sprague-Dawley-Ratten pro Geschlecht und Gruppe erhielten 7- oder 14-tägige Schlundsondengaben von Aluminiumchlorid, -citrat, -nitrat oder -sulfat in einer Dosierung von 30 mg Al/kg KG und Tag. Die Tiere erhielten zwei Wochen vor und während der Expositionszeit aluminiumarmes Futter und Trinkwasser. Es zeigten sich keine Zeichen von klinischer Toxizität. Urin und Faeces wurden bis 120 Stunden nach der letzten Dosisgabe gesammelt und danach die Tiere getötet und untersucht. Die individuellen Daten sind nicht berichtet und die Aluminiumkonzentrationen aus den Abbildungen abgeschätzt. Es ließ sich kein statistisch signifikanter Unterschied in der Aluminiumkonzentration mit Ausnahme der Knochen (männliche Tiere, 7 d: 0,18; 0,30; 0,61; 0,29 µg Al/g in Kontrolle, Aluminiumchlorid-, -citrat-, -nitrat-Gruppe) und der Nieren (männliche Tiere, 7 d: 0,2; 0,6 µg Al/g in Kontrolle bzw. Aluminiumcitrat-Gruppe und weibliche Tiere, 14 d: 0,2; 0,9 µg Al/g in Kontrolle bzw. Aluminiumcitrat-Gruppe) feststellen. Unabhängig vom Geschlecht war in den meisten Geweben sowohl bei den Kontrolltieren als auch nach Behandlung die Aluminiumkonzentration nach 14 Tagen niedriger als nach sieben Tagen, dabei war der Unterschied in Knochen und Blut statistisch signifikant. Nur im Rückenmark war nach 14 Tagen eine höhere Aluminiumkonzentration als nach sieben Tagen zu messen. Nach Bewertung der Autoren spiegeln die beobachteten Aluminiumgehalte die Exposition mit Aluminium durch Futter und Wasser vor der zweiwöchigen aluminiumfreien Vorexpositionsphase wider. Eine höhere Dosierung als 30 mg/kg KG und Tag könnte die systemische Aluminiumverteilung besser abbilden, ist allerdings aufgrund der limitierten Löslichkeit der Aluminiumsalze kaum möglich (ToxTest Alberta Research Council Inc. 2009).

Der plazentare Übergang von Aluminium ist für Ratten und Mäuse gezeigt (ATSDR 2008; EFSA 2008). Bei Wistar-Ratten führte die einmalige s.c. Gabe von 705 pg ^26^Al (als AlCl_3_) und 0,28 mg ^27^Al/ml (als AlCl_3_; 1,17 mg Al/kg KG; Vehikel: 0,45 M Natriumacetat-HCl-Puffer) am 15. Gestationstag zu erhöhten Aluminiumkonzentrationen in Plazenta sowie in Gehirn und Leber von Muttertieren und Feten am 20. Gestationstag. Von der injizierten ^26^Al-Menge wurde 0,21 % im Fetus und 0,2% in der Plazenta nachgewiesen, sowie 0,96% in der Leber der Muttertiere (Yumoto et al. 2000). Eine nachfolgende Untersuchung derselben Arbeitsgruppe mit derselben Applikation, jedoch am 16. Gestationstag und Untersuchung am 21. Gestationstag, ergab, dass von der gesamten injizierten ^26^Aluminiummenge 0,23 % im Fetus und 0,29 % in der Plazenta gefunden wurde (Yumoto et al. 2001). Daraus lässt sich folgern, dass die Aluminiumkonzentrationen in Plazenta und Fetus etwa gleich hoch sind und nach einmaliger s.c. Gabe, die ähnlich der inhalativen Gabe ist, ein sehr geringer Anteil zum Fetus gelangt. Es liegen keine Daten darüber vor, wie viel Aluminium während der gesamten Trächtigkeit zum Fetus gelangt. Verlässliche Angaben zur Aluminiumkonzentration im Blut trächtiger Tiere gibt es nicht.

Es liegt ein PBPK-Modell vor, das für einmalige orale und i.v. Exposition bei Menschen und Ratten validiert ist. Die Daten wurden anhand von oral oder i.v. verabreichtem ^26^Aluminiumchlorid oder ^26^Aluminiumcitrat mit einer Nachbeobachtung von bis zu 150 Wochen generiert, womit auch das kinetische Langzeitprofil für Plasma, Blut, Leber, Milz, Muskeln, Knochen, Gehirn, Niere und Urin erhoben wurde. Bei der Elimination aus Plasma und Blut konnten, am deutlichsten beim Menschen, mehrere aufeinanderfolgende Phasen beobachtet werden: (1) schneller, steiler Abfall der Aluminiumkonzentration innerhalb der ersten vier Stunden nach der Verabreichung mit einer HWZ im Minutenbereich; (2) langsamere Phase bis sieben Tage, HWZ im Tagebereich; (3) weiter verlangsamte Elimination bis 50 Wochen. Etwa 60 % der Dosis werden am ersten Tag ausgeschieden, die nächsten 20 % im Zeitraum von drei Wochen und die letzten 20 % noch später. Für den Menschen lässt sich mit dem Modell eine schnelle Abnahme im Blut innerhalb von 24 Stunden auf 2 % für Aluminiumcitrat bzw. 5 % für Aluminiumchlorid abschätzen. Gleichzeitig kommt es zu einer Umverteilung von Aluminium im restlichen Körper und in die Gewebekompartimente: Während die Aluminium-Mengen in allen anderen Kompartimenten in den ersten 24 Stunden ansteigen, nehmen diese im Plasma und im übrigen Körper nach Erreichen des maximalen Wertes parallel ab. Im Verlauf von 150 Wochen werden bei Aluminiumcitrat etwa 87 % des verabreichten Aluminiums im Urin ausgeschieden, in die Knochen werden maximal ca. 16 % eingelagert, während Aluminium im Gehirn kontinuierlich bis auf 0,05 % ansteigt. Bei Aluminiumchlorid erfolgt die Ausscheidung langsamer, so dass ca. 75 % mit dem Urin ausgeschieden werden, während sich Aluminium stärker in Knochen und Gehirn anreichert: In den Knochen werden Spitzenwerte von ca. 33 % erreicht, wogegen Aluminium im Gehirn kontinuierlich bis zu 0,1 % ansteigt (Hethey et al. 2021).

Bei der Ratte ist verglichen mit dem Menschen in verschiedenen Kompartimenten eine geringere HWZ zu beobachten (Tabelle 6) (Hethey et al. 2021). Aus der Abnahme im Blut auf 2 bis 5 % in 24 Stunden (s. o.) lässt sich eine HWZ im Blut für Aluminium am ersten Tag von etwa fünf Stunden für den Menschen abschätzen.

**Tab. 6:** Gewebe-HWZ von Aluminium bei Mensch und Ratte auf der Grundlage der PBPK-Modellparameter (Hethey et al. 2021)

**Organ**	**Mensch**		**Ratte**	
	**männlich**	**weiblich**	**jung (250 g KG)**	**alt (480 g KG)**
**Muskel**	35 h	33 h	5 h	7 h
**Nieren**	6 d	7 d	4 d	4 d
**Milz**	20 d	19 d	8 d	9 d
**Leber**	28 d	22 d	16 d	18 d
**Knochen**	198 Wo	162 Wo	11 Wo	13 Wo
**Gehirn** ^a)^	n. b.	n. b.	n. b.	n. b.

^a)^ Die verfügbaren experimentellen Daten zeigen keine Anzeichen für eine Elimination aus dem Gehirn.

d: Tage; h: Stunden; n. b.: nicht bestimmbar; Wo: Wochen

#### 3.1.3 Fazit

Aus einer Studie an inhalativ gegen schwer- und leichtlösliche Aluminiumverbindungen exponierten Arbeitern kann eine inhalative Resorption von 5 % für lösliche Aluminiumverbindungen abgeschätzt werden. Ebenfalls plausibel zur Expositionsabschätzung erscheinen orale Bioverfügbarkeiten von 0,21 % für Aluminiumsulfat, 0,08 % für Aluminiumcitrat, 0,05 % für Aluminiumchlorid und 0,05 % für Aluminiumnitrat. Die dermale Aufnahme löslicher Aluminiumverbindungen ist mit 0,002 % noch geringer. Nach der Aufnahme verteilt sich Aluminium gleichmäßig zwischen Plasma und zellulären Blutbestandteilen, kann die Blut-Hirn-, die Blut-Liquor-Schranke und die Plazenta-Schranke passieren und reichert sich in Knochen und Gehirn an (HWZ aufgrund fehlender Elimination nicht bestimmbar). Bei der Ratte sind verglichen mit dem Menschen geringere HWZ in den Kompartimenten zu beobachten. Die längste bestimmte HWZ betrifft die Knochen (Mensch 162 bis 198 Wochen). PBPK-Modelle für wiederholte Applikation liegen nicht vor. Die Elimination des systemisch resorbierten Aluminiums erfolgt fast ausschließlich mit dem Urin, der größte Anteil wird innerhalb der initialen schnellen Phase von etwa vier Stunden ausgeschieden.

### 3.2 Metabolismus

Aluminium wird nicht metabolisiert und liegt nur in der Oxidationsstufe Al^3+^ vor. Angaben zur Hydrolyse der löslichen Aluminiumverbindungen siehe „Hydrolysestabilität“.

## 4 Erfahrungen beim Menschen

### 4.1 Einmalige Exposition

Es liegen keine bewertungsrelevanten Daten mit löslichen Aluminiumverbindungen vor.

### 4.2 Wiederholte Exposition

#### Neurotoxizität

Bei Arbeitern, die gegen schwerlösliche Aluminiumverbindungen exponiert waren, wurden neurotoxische Effekte beobachtet (Hartwig und MAK Commission 2025; Klotz et al. 2018). Mit löslichen Aluminiumverbindungen liegen keine epidemiologischen Studien vor, jedoch muss vor allem aufgrund der besseren Bioverfügbarkeit ebenfalls mit einer neurotoxischen Wirkung gerechnet werden.

Durch die Verwendung aluminiumhaltiger Phosphatbinder im Dialysat zeigten Dialysepatienten Verwirrtheit, Gedächtnisstörungen und im fortgeschrittenen Stadium eine spezifische Enzephalopathie mit einem demenziellen Syndrom. Die Aluminiumenzephalopathie trat ab ca. 100 µg Aluminium/l Plasma auf, ist eine eigenständige Erkrankung und nicht mit der Demenz vom Alzheimer-Typ gleichzusetzen (Klotz et al. 2017).

Im Vergleich zu einer Kontrollgruppe waren bei neurodegenerativ erkrankten Personen Aluminiumgehalte in Blut, Haar und Gehirn erhöht (Exley und Clarkson 2020; Exley und Vickers 2014; Fiore et al. 2020; King et al. 2017; McLachlan et al. 2019; Mold et al. 2020). Diese Studien können für die Bewertung nicht herangezogen werden, da unklar ist, inwiefern eine Aluminiumexposition schon vor der Erkrankung stattgefunden hat.

### 4.3 Wirkung auf Haut und Schleimhäute

Hierzu liegen keine Daten vor.

### 4.4 Allergene Wirkung

#### 4.4.1 Hautsensibilisierende Wirkung

In der DKG-Standardreihe „Tätowiermittel“ wird Aluminiumchlorid als 2%ige Testzubereitung getestet. Mehrere Studien, in denen verschiedene Testzubereitungen auf ihre Eignung zur Feststellung einer Kontaktallergie untersucht wurden, kommen zu dem Schluss, dass eine 10%ige Testzubereitung nötig ist, um klinisch relevante Allergien gegen Aluminiumionen anzuzeigen. Eine 2%ige Testzubereitung ist hierzu bei Erwachsenen nicht geeignet (Bruze et al. 2008, 2020; Johansen et al. 2015; Siemund et al. 2012, 2022). Ferner erscheint der Ablesezeitpunkt sieben Tage nach Applikation zwingend, da ca. 15 % aller Testpersonen erst bei der zweiten Ablesung eine Woche nach Applikation eine positive Reaktion auf Aluminiumchlorid zeigten (Bruze et al. 2020). Auch eine 12%ige Testzubereitung von Aluminiumlactat (äquimolar bzgl. Aluminiumionen im Vergleich zu 10%iger Aluminiumchlorid-Lösung) ist für die Testung von Erwachsenen geeignet (Siemund et al. 2017, 2022).

Gelegentlich werden ringförmige Hautreaktionen auf die für den Epikutantest verwendeten, aus Aluminium bestehenden Testkammern beobachtet (Deleuran et al. 2019; Hedberg et al. 2020; Inerot et al. 2020; King und Moffitt 2018), was teilweise zufällig Aluminiumallergien aufdeckt (Brodbaker und Pratt 2009; Kullberg et al. 2020).

Es liegen keine Fälle von beruflicher Kontaktdermatitis durch lösliche Aluminiumverbindungen vor.

Weiterhin gibt es nur wenige Studien zur Bestimmung der Sensibilisierungsquote gegenüber Aluminiumverbindungen (und elementarem Aluminium) mit Epikutantests in größeren Patientenkollektiven, die nicht im direkten Zusammenhang mit Impfreaktionen oder der Verwendung aluminiumhaltiger Antitranspirantien stehen (Novack et al. 2022). Von 5448 Erwachsenen, die zwischen 2010 und 2017 mit Verdacht auf eine Kontaktallergie auf Aluminium (k. w. A. über vermutete Ursachen oder Verbindungen) mit Aluminiumchlorid (10 % in Vaseline) und Aluminiumlactat (12 % in Vaseline) getestet wurden, reagierten 48 Personen (0,9 %) auf mindestens eins der Salze. Auf Aluminiumchlorid reagierten 34 der 5448 Personen positiv (0,6 %), auf Aluminiumlactat 30 der 5448 Personen (0,6 %), wobei 16 Personen auf beide Salze positiv reagierten. Überwiegend wurden einfach positive Reaktionen beobachtet (Aluminiumchlorid: einfach positive Reaktion bei 19 von 34; Aluminiumlactat: einfach positive Reaktion bei 16 von 30) und wenige dreifach positive Reaktionen (Aluminiumchlorid: 4 von 34; Aluminiumlactat: 1 von 30). Die Ablesung erfolgte am 3. oder 4. und am 7. Tag (Siemund et al. 2022).

Insgesamt 916 Personen, davon 811 mit Nachweis einer nicht beruflich bedingten Kontaktdermatitis, wurden mit Aluminiumchlorid getestet (k. A. zur Konzentration, vermutlich mit 2 %, k. A. über Ablesezeitpunkt). Davon reagierten insgesamt sechs Personen (0,7 %), die dem Kollektiv der Personen mit nicht beruflich bedingter Kontaktdermatitis zuzuordnen waren, positiv (Geier und Schubert 2021).

Die meisten Studien betreffen eine Aluminiumsensibilisierung in Folge der Injektion von Impfstoffen, die schwerlösliches Aluminiumoxyhydroxid (24623-77-6), Aluminiumphosphat (7784-30-7, 22784-12-9), Aluminiumkaliumdisulfat, sowie gemischte Aluminiumsalze enthalten können (z. B. Bergfors et al. 2019; Bruze et al. 2008; Danielsson und Eriksson 2021; Deleuran et al. 2019; Goiset et al. 2018; Inerot et al. 2020; Kullberg et al. 2020; Laera et al. 2023; Xará et al. 2023) oder allergenspezifischen Immuntherapien (Hyposensibilisierung) (Bruze et al. 2008; Hindsén 2005; Netterlid et al. 2013), wobei die Aluminiumverbindungen als Adjuvans eingesetzt wurden. Betroffen waren vor allem Kinder und Jugendliche, seltener auch Erwachsene. Etwa 1 % der mit aluminiumhaltigen Impfstoffen geimpften Kinder entwickelten eine Kontaktallergie gegen Aluminium, die sich in der Regel mit Granulomen und Ausschlag äußerte (Trollfors et al. 2005). In Einzelfällen wurde auch über Kontaktdermatitis durch aluminiumhaltige Sonnencreme bei Kindern berichtet, bei denen die Sensibilisierung gegen Aluminiumionen im Zusammenhang mit Impfstoffen steht (Badaoui 2023; Hoffmann et al. 2022). Diese Fälle, bei denen die Sensibilisierung im Kontext mit einer s.c. Applikation von Aluminiumverbindungen als Komponente von Impfstoffen oder Hyposensibilisierungen steht, weisen auf systemische Verfügbarkeit und mögliche Sensibilisierung hin. Sie sind nicht direkt auf die Situation am Arbeitsplatz übertragbar, untermauern jedoch das Wirkprinzip der Materialien und vervollständigen das Gesamtbild.

Des Weiteren wurde im nicht-beruflichen Kontext die Verwendung aluminiumhaltiger Kosmetika (z. B. Lidschatten, Mascara, Gesichtsmasken) als Ursache für eine Sensibilisierung berichtet (Borowska und Brzóska 2015).

Eine 28-jährige Frau mit einem Ekzem in der Achselhöhle, welches im Zusammenhang mit der Verwendung eines Aluminiumchlorid-haltigen Deodorants stand, reagierte mit einer 3+ Reaktion auf Aluminiumchlorid (2 % in Vaseline) am 2. und 4. Tag, reagierte jedoch nicht auf eine leere Finn-Kammer aus Aluminium. Die Autoren vermuten, dass die Freisetzung von Aluminiumionen aus der Finn-Kammer zu gering war, um eine Reaktion auszulösen (Garg et al. 2010). Eine Spätablesung am 7. Tag erfolgte nicht.

Ein 35-jähriger Patient, welcher aufgrund von Hautreaktionen auf Sonnencremes und Deodorants einer Epikutantestung unterzogen wurde, entwickelte an jeder Stelle, an der eine Finn-Kammer appliziert wurde, eine 2+-Hautreaktion. Bei der erneuten Testung reagierte er wieder positiv auf eine leere Finn-Kammer sowie ebenfalls positiv auf weitere Allergene (z. B. Duftstoffe, p-Phenylendiamin, Formaldehyd), welche in Kunststoffkammern getestet wurden. Die Sonnencreme-Testserie ergab mit und ohne UV-Exposition negativ Ergebnisse. Die Reaktion auf Aluminium wurde im Zusammenhang mit der Reaktion auf Deodorant und Sonnencreme als relevant bewertet (King und Moffitt 2018).

#### 4.4.2 Atemwegssensibilisierende Wirkung

Hierzu liegen keine Daten vor.

### 4.5 Reproduktionstoxizität

In einer Fall-Kontroll-Studie wurden an 111 Müttern, die im zweiten Trimester fehlgebildete Feten hatten, im Serum 44 Metalle bestimmt. Die Kontrollgruppe bestand aus 90 Müttern mit im zweiten Trimester normal entwickelten Feten. Feten mit bekannten genetischen Defekten wurden ausgeschlossen. Die Fälle wurden in eine ZNS (zentrales Nervensystem)-Gruppe (n = 17; 15,3 %; Akranie, Anencephalie, Fehlen des Corpus callosum, Hydrocephalus, Myelomeningocele, Spina bifida, Dandy-Walker-Syndrom) und eine Gruppe mit anderen Fehlbildungen (n = 94; 84,7 %) eingeteilt. Die Aluminiumkonzentrationen im Serum der Mütter mit Feten, die Fehlbildungen im ZNS hatten (6,45 ± 15,15 μg/l), waren statistisch signifikant höher als die Aluminiumkonzentrationen im Serum der Mütter mit Feten, die andere Fehlbildungen (1,44 ± 4,21 μg/l, p < 0,0006) oder keine aufwiesen (0,11 ± 0,51 μg/l, p < 0,0006). Für alle anderen Metalle (darunter auch Quecksilber und Blei) ergaben sich keine Assoziationen zu Fehlbildungen. Wie die Autoren selbst erklären, ist die geringe Fallzahl der ZNS-Fehlbildungen eine Schwäche der Studie (Troisi et al. 2019). Alkoholkonsum als bedeutender Confounder wurde in der Studie nicht erfragt.

#### Entwicklungsneurotoxizität

Es liegen keine Untersuchungen zur Entwicklungsneurotoxizität vor, sodass nicht klar ist, ob Feten empfindlicher reagieren als Erwachsene. Im Folgenden wird daher zumindest die Sensitivität von sich noch in der Entwicklung befindenden Kindern im Vergleich zu Erwachsenen betrachtet.

Von Kindern und Erwachsenen mit normaler Nierenfunktion liegen mehrere Fallberichte zu skelettalen Veränderungen (wie Osteomalazie) aufgrund längerer Nutzung von aluminiumhaltigen Antazida zur Therapie von gastrointestinalen Beschwerden vor. Die skelettalen Effekte sind Sekundäreffekte einer Hypophosphatämie und Phosphatdepletion, die durch die Aluminium-induzierte Störung der Phosphataufnahme aus der Nahrung resultiert (ATSDR 2008). Daraus einen Schluss auf die unterschiedliche Empfindlichkeit von Kindern und Erwachsenen zu ziehen, ist nicht möglich.

Auch aus den Studien zu Dialysepatienten (siehe Abschnitt 4.2) lässt sich nicht ableiten, ob Kinder empfindlicher als Erwachsene reagieren. Zudem sind Effekte nach i.v. Gabe (Dialyse) bei verminderter Aluminiumausscheidung aufgrund der reduzierten Nierenfunktion nicht zu Bewertung der Toxizität von Aluminium am Arbeitsplatz geeignet.

**Fazit:** Es liegen keine Untersuchungen vor, um zu bewerten, ob Feten genauso empfindlich oder empfindlicher als Erwachsene auf Aluminium-induzierte Neurotoxizität reagieren.

### 4.6 Genotoxizität

Hierzu liegen keine Daten vor.

### 4.7 Kanzerogenität

#### 4.7.1 Explorative Studien

In einer retrospektiven Studie wurden von Januar 1993 bis Dezember 2001 Frauen (n = 1344), die eine Brustkrebserkrankung überlebt hatten, angeschrieben und nach der Verwendung von Antitranspirantien und Deodorants in Verbindung mit einer regelmäßigen Rasur der Achselhöhle befragt. Rückmeldungen kamen von 437 Frauen im Alter von 31 bis 94 Jahren. Davon verwendeten nur 40 Patientinnen keine Antitranspirantien/Deodorants und rasierten sich auch nicht. Diese waren bei der Diagnose im Mittel 68 Jahre alt, die regelmäßigsten Nutzerinnen 53 Jahre (p < 0,0001). Frauen, die vor ihrem 16. Lebensjahr mit der Verwendung von Antitranspirantien/Deodorants und Rasur begannen, waren im Mittel 57 Jahre, die anderen 67 Jahre (p < 0,0001). Unabhängig davon waren weder die Rasur allein noch die alleinige Verwendung von Antitranspirantien/Deodorants mit einem statistisch signifikant früheren Diagnosealter für Brustkrebs verbunden (McGrath 2003). Ob und welche Aluminiumverbindungen in den Produkten enthalten waren, wurde nicht erfasst. Messungen der Aluminiumkonzentrationen in Brustgewebe, Serum oder Urin erfolgten nicht. Es wurde lediglich unterschieden, wann der Brustkrebs auftrat. Eine Kontrollgruppe fehlt. Es ist unklar, wie viele Patientinnen nur Antitranspirantien (in der Regel mit Aluminiumsalz) oder nur Deodorants (meist ohne Aluminiumsalz) verwendeten. Weiterhin wurde keine multivariate Analyse durchgeführt. In der Studie ist beschrieben, dass es statistisch signifikante Unterschiede zwischen den Expositionsgruppen bezüglich des Alkoholkonsums gab, welche in der Auswertung nicht berücksichtigt wurden. Insgesamt ist diese Studie nicht geeignet, um eine Brustkrebsentstehung auf die Verwendung von aluminiumhaltigen Antitranspirantien/Deodorants zurückzuführen.

#### 4.7.2 Fall-Kontroll-Studien

Die Fall-Kontroll-Studien von Mirick et al. (2002) und Fakri et al. (2006) werden nicht zur Bewertung der Kanzerogenität herangezogen, da keine Angaben zu Aluminiumverbindungen in den verwendeten Produkten vorliegen. Messungen zu Aluminiumkonzentrationen in Brustgewebe, Serum oder Urin erfolgten nicht.

In einer Krankenhaus-basierten Fall-Kontroll-Studie wurde der Zusammenhang zwischen der Verwendung von Antitranspirantien (in der Veröffentlichung als Achselkosmetikprodukte benannt) und dem Brustkrebsrisiko untersucht. Folgendes wurde geprüft: 1) Häufigkeit der Antitranspirantien/Deodorants-Verwendung bei Brustkrebspatientinnen verglichen mit Kontrollpatientinnen, 2) Aluminiumkonzentrationen im Brustgewebe der Krebspatientinnen verglichen mit denen der Kontrollpatientinnen, 3) Zusammenhang zwischen der Verwendung von Antitranspirantien/Deodorants und der Aluminiumkonzentration im Brustgewebe. Hierzu wurden insgesamt 209 Brustkrebs- und 209 Kontroll-Patientinnen (angepasst nach Alter) zu persönlichen Lebensgewohnheiten, Ernährung, körperlicher Aktivität, persönlicher Hygiene, Verwendung von Antitranspirantien, Hormonstatus und genetischen Faktoren befragt. Gewebeproben wurden von 100 Brustkrebspatientinnen und 52 Kontrollpatientinnen untersucht. Die Frauen wurden anhand ihres Alters bei Beginn der Verwendung von Antitranspirantien/Deodorants in drei Gruppen eingeteilt: unter 30 Jahre, zwischen 30 und 50 Jahre, über 50 Jahre und ob sie sie in den fünf Jahren vor der Diagnose (Patientinnen) bzw. vor dem Interview (Kontrollpersonen) verwendet hatten. Weiterhin wurde eine Einteilung nach der Häufigkeit der Anwendung vorgenommen: nie, 1–4-mal im Monat, 2–6-mal pro Woche, täglich und mehrmals täglich. Die mehrmals tägliche Anwendung von Antitranspirantien/Deodorants mit Beginn im Alter von unter 30 Jahren war mit einem statistisch signifikant erhöhten Brustkrebsrisiko (18 Fälle, OR: 3,88, 95-%-KI: 1,03–14,66) verbunden, adjustiert nach Alter, Brustkrebs-Familienanamnese, Familienanamnese anderer Krebsarten, Vorgeschichte einer gutartigen Brusterkrankung, Alter der ersten Menstruation, Alter bei Erstgeburt, Alter bei Menopause, Wechseljahre-Status, Hormonersatztherapie, Body-Mass-Index (BMI) und Alkoholkonsum. In den anderen Anwendungsgruppen war das adjustierte Brustkrebsrisiko nicht erhöht. Für alle 100 untersuchten Fälle betrug der Median der Aluminiumkonzentrationen im Brustgewebe 5,77 nmol/g Trockengewicht (Bereich 2,29–12,9 nmol/g) und war damit statistisch signifikant erhöht im Vergleich zu 3,77 nmol/g Trockengewicht (Bereich 2,47–5,78) bei den 52 Kontrollpatientinnen. In Tabelle 7 sind die Aluminiumkonzentrationen im Brustgewebe der Krebsfälle und der Kontrollpatientinnen in Bezug auf die Anwendung von Antitranspirantien/Deodorants angegeben. In Beziehung zur Anwendung von Antitranspirantien/Deodorants „Beginn unter 30 Jahren“ und „fünf Jahre vor Diagnose“ ergab sich keine statistisch signifikante Tumorlokalisation. Die Aluminiumkonzentration war im oberen, äußeren Quadranten bei den Brustkrebsfällen höher als in den anderen Quadranten. Bei den Kontrollpatientinnen lagen die Mediane der Aluminiumkonzentrationen bezogen auf die Quadranten und der Anwendungshäufigkeit in ähnlicher Größenordnung. Die Autoren geben als Limitierungen der Studie an, dass die selbst berichteten Informationen unvollständig, nicht korrekt und verschieden zwischen Fällen und Kontrollen sein könnten. Weiterhin diskutieren sie als möglichen Bias die Mischung aus Inzidenz und Prävalenz der Fälle, sowie die geringe Fallzahl in der Gruppe der täglichen Anwenderinnen. Es wird auch darauf verwiesen, dass die Konzentrationen anderer Metalle im Brustgewebe deutlich erhöht sein könnten (Linhart et al. 2017). Als weitere Limitierungen der Studie sind zu nennen, dass in den Fragebögen keine Unterscheidung zwischen der Verwendung von Antitranspirantien und Deodorants möglich war und auch keine Aussage getroffen werden konnte, ob eine Behandlung mit Chemotherapeutika vorlag. Die Interviewdurchführung der Kontrollen und Fälle fand zu unterschiedlichen Zeitpunkten statt. Es ist unklar, wie und wann die Auswahl der Kontrollpersonen und Patientinnen stattfand. Die Studie enthält einen möglichen Selektionsbias in der Kontrollgruppe, da diese aus Patientinnen mit einer vorgenommenen Mammareduktionsplastik bestand und diese Gruppe nicht repräsentativ für die weibliche Allgemeinbevölkerung ist. Da die Ergebnisse der Altersgruppen 30 bis 50 Jahre und über 50 Jahre fehlen, ist ein Berichtsbias zu nennen, weiterhin ist von einem Recall-Bias auszugehen. Fraglich ist, ob bei mehrmals täglicher Anwendung tatsächlich Antitranspirantien verwendet wurden, da deren Wirkdauer deutlich länger ist. Insgesamt ist die Studie nicht belastbar, um einen Zusammenhang zwischen der Verwendung von aluminiumhaltigen Antitranspirantien/Deodorants und einem erhöhten Brustkrebsrisiko festzustellen.

In einer beschreibenden Korrelationsstudie wurde die Beziehung zwischen Brustkrebs und der Verwendung von aluminiumhaltigen Antitranspirantien, sowie von Haarfärbemitteln untersucht (Mousavi und Vaghar 2021). Die Studie ist nicht aussagekräftig, da wesentliche epidemiologische Methoden (Studiendesign, Auswahl von Fällen und Kontrollen, Erhebung der Exposition) nicht verständlich berichtet oder in sich widersprüchlich sind (z. B. Geschlechterverteilung der Studienpopulation). Die Studie wird nicht zur Bewertung des Brustkrebsrisikos beim Menschen herangezogen.

**Tab. 7:** Aluminiumkonzentrationen im Brustgewebe der Krebsfälle und der Kontrollpatientinnen in Bezug auf die Anwendung von Antitranspirantien/Deodorants mit Beginn unter 30 Jahren (Linhart et al. 2017)

**Benutzung Antitranspirantien/Deodorants**	**Aluminiumkonzentration Median (IQR^a)^)** **[nmol/g Trockengewicht]**			
	**Fälle**	**Anzahl**	**Kontrollen**	**Anzahl**
	5,77 (2,29–12,90)	100	3,77 (2,47–5,78)	52
Nie	3,58 (1,72–9,25)	28	2,74 (1,90–4,21)	11
Mehrmals pro Woche	7,77 (4,74–11,40)	9	3,07 (2,75–4,52)	4
Täglich	6,07 (2,21–14,89)	53	4,34 (2,67–6,42)	34
Mehrmals pro Tag	11,29 (3,62–13,21)	9	2,51 (1,86–4,86)	3

^a)^ Independent samples t-test mit log10(x + 1)-transformierten Daten

IQR: Interquartilsabstand

#### 4.7.3 Kohortenstudien

Es liegen keine Kohortenstudien mit löslichen Aluminiumverbindungen vor.

#### 4.7.4 Metaanalysen

Um zu klären, ob a) biologische Daten einen Zusammenhang zwischen der Verwendung von Achseldeodorantien oder -antitranspirantien und Brustkrebs bei Frauen belegen, b) die Verwendung dieser Produkte das Brustkrebsrisiko erhöht und c) ein kausaler Zusammenhang zwischen der Verwendung von Achseldeodorantien/-antitranspirantien und einem erhöhten Brustkrebsrisiko besteht, wurden 59 Studien identifiziert, die bis 2007 veröffentlicht wurden. Von den 59 in Frage kommenden Studien wurden 19 als geeignet für die Bewertung der Hypothesen erachtet. Die Autoren kamen zu dem Schluss, dass es „keine wissenschaftlichen Belege zur Unterstützung der Hypothese“ gibt, dass die Verwendung von aluminiumhaltigen Antitranspirantien die Inzidenz von Brustkrebs im oberen äußeren Quadranten der menschlichen Brust erhöht (Namer et al. 2008).

In einer sehr kurz dargestellten Metaanalyse wird aus den Studien von Fakri et al. (2006) und Mirick et al. (2002) ein OR von 0,81 (95-%-KI: 0,51–1,28) für Brustkrebs bei Anwendung von Deodorants berechnet (Hardefeldt et al. 2013). Eine zweite Analyse berechnet aus denselben Studien ein OR von 0,4 (95-%-KI: 0,35–0,46) für Brustkrebs bei Anwendung von Antitranspirantien (Allam 2016). Aufgrund fehlender Angaben, welche Studien in die Metanalyse einbezogen wurden, wird eine weitere Metaanalyse von Osto et al. (2022) nicht bei der Bewertung berücksichtigt.

#### 4.7.5 Fazit

Aus den vorliegenden Daten kann kein Zusammenhang zwischen der Entstehung von Brustkrebs und der Verwendung aluminiumhaltiger Antitranspirantien abgeleitet werden. Erhöhte Mengen an Aluminium im Tumorgewebe der Brust weisen darauf hin, dass Aluminium wie andere Mineralstoffe und Metalle vermehrt im Tumorgewebe eingelagert wird (siehe Abschnitt 2.4). Ein erhöhter Aluminiumgehalt im malignen Brustgewebe ist deshalb nicht als Ursache von Brustkrebs anzusehen, sondern vielmehr eine Konsequenz veränderter Transportvorgänge in malignem Gewebe.

## 5 Tierexperimentelle Befunde und In-vitro-Untersuchungen

### 5.1 Akute Toxizität

#### 5.1.1 Inhalative Aufnahme

In einem Limit-Test aus dem Jahr 2010 nach OECD-Prüfrichtlinie 403 wurden jeweils fünf männliche und weibliche Ratten (Crl:WI(Han)) vier Stunden lang nur über die Nase gegen ein Aluminiumchloridhydroxysulfat-Aerosol exponiert. Es trat keine substanzbedingte Mortalität auf. Lethargie, Piloarrektion und gekrümmte Haltung wurden bis zwei Tage nach der Exposition beobachtet. Die makroskopische Untersuchung ergab keine substanzbedingten Befunde. Die LC_50_ der Testsubstanz war größer als 5000 mg/m^3^ (ECHA 2020 b).

In einer weiteren Studie aus dem Jahr 2010, ebenfalls nach OECD-Prüfrichtlinie 403 durchgeführt, wurden jeweils fünf männliche und weibliche Wistar-Ratten pro Gruppe vier Stunden lang nur über die Nase gegen ein Dialuminiumchloridpentahydroxid-Aerosol exponiert. Die Konzentration von 3600 mg/m^3^ wirkte innerhalb des ersten Tages für alle Tiere letal und führte während der Exposition zu erschwerter Atmung. Die makroskopische Untersuchung dieser Tiere zeigte zahlreiche grau-weiße oder rötliche Herde in der Lunge. Bei 1200 mg/m^3^ starb ein männliches Tier. Bei den überlebenden Tieren wurde nach der Exposition Lethargie, gebückte Haltung, gestörte Atmung sowie Bauch- und Seitenlage beobachtet. Eines dieser Tiere hatte ebenfalls zahlreiche grau-weiße oder rötliche Herde in der Lunge. Die LC_50_ der Testsubstanz lag zwischen 1000 und 5000 mg/m^3^ (ECHA 2022 c).

#### 5.1.2 Orale Aufnahme

In den REACH-Registrierungsdossiers finden sich für Aluminiumammoniumdisulfat, -chlorhydrat, -chlorid, -diacetat, -kaliumsulfat, -lactat, -nitrat und -sulfat LD_50_-Werte von > 2000 mg/kg KG für Ratten (ECHA 2019, 2020 a, c, 2022 a, b, c, 2023 b, c).

#### 5.1.3 Dermale Aufnahme

In den REACH-Registrierungsdossiers werden für Aluminiumammoniumdisulfat, Aluminiumchlorid, basisch, Aluminiumchlorhydrat und Aluminiumchloridhydroxysulfat LD_50_-Werte für Ratten von > 2000 mg/kg KG berichtet (ECHA 2020 a, b, 2022 c, 2023 a). Für Aluminiumsulfat war die LD_50_ für Ratten > 2000 mg/kg KG und für Kaninchen > 5000 mg/kg KG (ECHA 2023 c).

### 5.2 Subakute, subchronische und chronische Toxizität

#### 5.2.1 Inhalative Aufnahme

##### 5.2.1.1 Wirkungen auf die Lunge

Die Inhalationsstudien mit löslichen Aluminiumverbindungen sind in Tabelle 8 dargestellt.

**Tab. 8:** Toxizität löslicher Aluminiumverbindungen nach wiederholter inhalativer Exposition

**Spezies,** **Stamm, ** **Anzahl pro Gruppe**	**Exposition**	**Befunde**	**Literatur**
Ratte, Wistar, 5 ♂	**2 Wo,** **AlCl_3_,** 0, 1, 5, 25 mg/m^3^ (0,2; 1; 5 mg Al/m^3^), 6 h/d, 5 d/Wo, wässrige Lösung (6 %) des wasserfreien Aluminiumchlorids, nur über die Nase, Flüssigaerosol, OECD TG 412	**ab 1 mg/m^3^ (0,2 mg Al/m^3^): LOAEC** BALF: Gesamtzellzahl ↑, abs. u. rel. Neutrophile ↑, rel. Monozyten ↑, rel. Makrophagen ↓, Gesamtprotein ↑, LDH ↑, NAG ↑, GGT ↑, Nase: Nasenhöhle, Schnittebene IV: Erosion/Ulzera (1/5); **ab 5 mg/m^3^ (1 mg Al/m^3^):** Lunge: Entzündung, gemischte Zellen, minimal (2/5), Nase: Nasenhöhle, Schnittebene III: Erosion/Ulzera, minimal bis leicht (3/5), Schnittebene IV: Erosion/Ulzera, mäßig bis stark (5/5); **25 mg/m^3^ (5 mg Al/m^3^):** Atemgeräusche (1/5), BALF: abs. Makrophagen ↑, Lymphozyten ↑, epitheliale Zellen ↑, Lunge: Entzündung, gemischte Zellen, minimal bis mäßig (5/5), Nase: Nasenhöhle, Schnittebene III u. IV: Erosion/Ulzera, minimal bis stark (5/5)	BASF SE 2019 b
Ratte, Wistar, 10 ♂, 10 ♀	**13 Wo,** **AlCl_3_**, 0; 0,1; 0,5; 2,0 mg/m^3^ (0,02; 0,1; 0,4 mg Al/m^3^), 6 h/d, 5 d/Wo, wässrige Lösung (0,2 %, pH-Wert 3,86–4,0) des wasserfreien Aluminiumchlorids, MMAD: 0,90–1,39 μm, GSD: < 3, Reinheit > 99 %, nur über die Nase, Flüssigaerosol, OECD TG 413	**ab 0,1 mg/m^3^ (0,02 mg Al/m^3^): LOAEC** ♂/♀: BALF: ALP ↑ (≤ 1,5-fach, aber signifikant), Gesamtprotein ↑ (≤ 1,5-fach, aber signifikant), Larynx: epitheliale Veränderungen Grad 1, Trachea: Carina-Spitze minimale Abflachung des Epithels mit Verlust der Flimmerhärchen, Partikel in Histiozyten (nicht advers; 10/10 ♂; 9/10 ♀), ♂: Lunge: Histiozyten ↑ (1/10), Blut: Neutrophile ↑, Lymphozyten ↓ (innerhalb oder marginal außerhalb historischer Kontrollbereiche), ♀: BALF: abs. u. rel. Neutrophile ↑, Blut: abs. Monozyten ↑ (innerhalb historischem Kontrollbereich), mediastinale Lymphknoten: makroskopisch vergrößert (0/10, 1/10, 3/10, 10/10 bei 0,02; 0,1; 0,4 mg Al/m^3^); **ab 0,5 mg/m^3^ (0,1 mg Al/m^3^):** ♂/♀: BALF: Lymphozyten ↑, Monozyten ↑, GGT ↑, Histiozyten ↑ (10/10), Blut: abs. u. rel. Neutrophile ↑, Lymphozyten ↓, minimale Entzündung, gemischte Zellen, (10/10 ♂, 7/10 ♀), mediastinale und tracheobronchiale Lymphknoten: Makophagen-Aggregate, ♂: BALF: abs. u. rel. Neutrophile ↑, Gesamtzellzahl ↑, LDH ↑, Blut: rel. Monozyten ↑ (innerhalb des historischen Kontrollbereichs), minimale Zelltrümmer in Alveolen (7/10); minimale (multi)fokale lymphoide Infiltration (6/10); **2 mg/m^3^ (0,4 mg Al/m^3^): ** ♂/♀: abs. u. rel. Lungengewicht ↑, Lunge: minimale bis leichte Makrophagen-Aggregate mit aufgenommenen Partikeln in BALF (10/10 ♂, 7/10 ♀), Zelltrümmer in Alveolen mäßig ↑ (10/10 ♂), minimal oder leicht (8/10 ♀); mäßige (multi)fokale lymphoide Infiltration (9/10 ♂, 7/10 ♀), mäßige Entzündung (gemischte Zellen) (10/10 ♂, 10/10 ♀), tracheobronchiale Lymphknoten: makroskopisch vergrößert, Larynx: epitheliale Veränderungen Grad 2, ♂: BALF: NAG ↑, Nase: Nasenhöhle, Schnittebene IV: Erosion/Ulzera 4/10; mediastinale Lymphknoten: makroskopisch vergrößert, ♀: BALF: LDH ↑	BASF SE 2019 a
Ratte, Sprague Dawley, 10 ♂	**5, 9, 13, 18, 26 Wo,** **AlCl_3_**, 0; 1,28 mg/m^3^ (0,26 mg Al/m^3^), 6 h/d, 5 d/Wo, MMAD: < 10 μm, Staub, Nachbeobachtung 63 d	**1,28 mg/m^3^ (0,26 mg Al/m^3^):** BALF: Lysozym ↑, Protein ↑, ALP ↑	Finelli et al. 1981
Ratte, F344, 10 ♂, 10 ♀ (davon je 5 für Histopathologie)	**6 Mo,** **ACH**, 0; 0,25; 2,5; 25 mg/m^3^ (ca. 0,08; 0,77; 7,7 mg Al/m^3^), 6 h/d, 5 d/Wo, EAD: 50 %: 1,6; 1,20; 1,53 μm, 84 %: 6,20; 5,78; 5,34 μm, GSD 3,88; 4,82; 3,49, Staub	**ab 0,25 mg/m^3 ^(ca. 0,08 mg Al/m^3^):** ♂/♀: peribronchiale Lymphknoten: Mikrogranulome (1/10, k. A. zum Geschlecht), Lunge: alveoläre Makrophagen leicht ↑ (k. w. A.); **ab 2,5 mg/m^3 ^(ca. 0,77 mg Al/m^3^): ** ♂/♀: peribronchiale Lymphknoten: Mikrogranulome (10/10), Lunge: multifokale granulomatöse Pneumonie (10/10) (gekennzeichnet durch Proliferation und/oder Infiltration von mononukleären Entzündungszellen u. großen Makrophagen in die Alveolen); **25 mg/m^3^ (ca. 7,7 mg Al/m^3^):** ♂/♀: KG ↓, Lunge: rel. u. abs. Gewicht ↑, multifokale Aggregate zellulärer Makrophagen begleitet von alveolären Zelltrümmern (10/10), Nase: Becherzellen ↑	Steinhagen et al. 1978; Stone et al. 1979
Meerschweinchen, Hartley, 10 ♂, 10 ♀ (davon je 5 für Histopathologie)	**6 Mo,** **ACH**, 0; 0,25; 2,5; 25 mg/m^3^ (ca. 0,08; 0,77; 7,7 mg Al/m^3^), 6 h/d, 5 d/Wo, EAD: 50 %: 1,6; 1,20; 1,53 μm, 84 %: 6,20; 5,78; 5,34 μm, GSD 3,88; 4,82; 3,49, Staub	**ab 0,25 mg/m^3 ^(ca. 0,08 mg Al/m^3^):** ♂/♀: Lunge: alveoläre Makrophagen leicht ↑ (3/10, k. A. zum Geschlecht); **ab 2,5 mg/m^3 ^(ca. 0,77 mg Al/m^3^):** ♂/♀: peribronchiale Lymphknoten: Mikrogranulome (10/10), Lunge: multifokale granulomatöse Pneumonie (10/10) (gekennzeichnet durch Proliferation und/oder Infiltration von mononukleären Entzündungszellen u. großen Makrophagen in die Alveolen); **25 mg/m^3 ^(ca. 7,7 mg Al/m^3^): ** ♂/♀: Lunge: rel. Gewicht ↑, minimale Ödeme	Steinhagen et al. 1978; Stone et al. 1979

abs.: absolute(s); ACH: Aluminiumchlorhydrat; Al: Aluminium; ALP: Alkalische Phosphatase; BALF: bronchoalveoläre Lavageflüssigkeit; BALT: Bronchien-assoziiertes lymphatisches Gewebe; BUN: Blutharnstoff-Stickstoff; d: Tage; EAD: massenäquivalenter aerodynamischer Durchmesser; FOB: functional observational battery; GGT: γ-Glutamyltransferase; GSD: geometrische Standardabweichung; k. A.: keine Angabe; LDH: Lactatdehydrogenase; MMAD: massenmedianer aerodynamischer Durchmesser; NAG: β-N-Acetylglucosaminidase; rel.: relative(s); Wo: Woche(n)

Die Studie mit der niedrigsten LOAEC ist eine 13-Wochen-Inhalationsstudie an Wistar-Ratten nach OECD-Prüfrichtlinie 413, in der männliche und weibliche Tiere an sechs Stunden pro Tag, fünf Tage pro Woche nur über die Nase gegen ein Aerosol einer wässrigen Lösung (0,2%ig) des wasserfreien Aluminiumchlorids exponiert wurden. Die Zielkonzentrationen waren 0,1; 0,5 und 2 mg Aluminiumchlorid/m^3^ (0,02; 0,1; 0,4 mg Al/m^3^). Eine Kontrollgruppe wurde gegen befeuchtete Luft exponiert (BASF SE 2019 a). Da festes Aluminiumchlorid aufgrund seiner extremen Hygroskopie nicht verwendet werden konnte (Düse und Apparatur verstopften), wurde eine wässrige Lösung eingesetzt. Ergebnisse von Hydrolysekinetik-Experimenten zeigten, dass die Hydrolyse von wasserfreiem Aluminiumchlorid bei Kontakt mit der Luft sehr schnell abläuft. Es wird erwartet, dass die Hydrolysereaktion noch schneller abläuft, wenn die Substanz als Staubaerosol in der Luft vorhanden ist, da die Kontaktfläche zwischen der Substanz und der Umgebungsluft größer ist. Unterschiedlich erzeugte Hydrolyseprodukte an der Luft verglichen mit Wasser zeigten, dass Aluminiumchloridhexahydrat (AlCl_3_ × 6 H_2_O) immer das Hauptreaktionsprodukt war (BASF SE 2018). Daher wurde in der vorliegenden Studie wasserfreies Aluminiumchlorid gemahlen und in Reinstwasser gelöst, wo es ein stabiles Aluminiumchloridhexahydrat bildete und nach Verdünnung als wässrige Lösung versprüht. Die Stabilität des so gebildeten Aluminiumchloridhexahydrats in wässriger Lösung wurde durch NMR-Analysen untersucht. Alle Kaskadenimpaktor-Messungen der Partikelgröße ergaben MMAD zwischen 0,90 und 1,39 μm mit GSD < 3. Somit waren die Aerosole für Ratten in hohem Maße lungengängig (ECHA 2022 a). Vorversuche wurden mit wässrigen Lösungen von Aluminiumchlorid in verschiedener Konzentration durchgeführt, wobei sich die 0,2%ige Lösung als am geeignetsten für die Hauptstudie erwies (Leibold 2023).

Die Ergebnisse sind im Detail in Tabelle 9 dargestellt. Bei der niedrigsten Konzentration von 0,1 mg/m^3^ (0,02 mg Al/m^3^) waren in der BALF der männlichen und weiblichen Tiere Gesamtprotein und ALP erhöht, bei den weiblichen Tieren zusätzlich die absolute Zahl und der relative Anteil an Neutrophilen. Basierend darauf sehen die Studienautoren 0,1 mg/m^3^ (0,02 mg Al/m^3^) als LOAEC für die weiblichen und als NOAEC für die männlichen Ratten. Die hämatologische Untersuchung ergab ab 0,5 mg/m^3^ (0,1 mg Al/m^3^) eine erhöhte absolute und relative Neutrophilenzahl und eine verringerte Lymphozytenzahl bei männlichen und weiblichen Tieren, was wahrscheinlich auf die lokale Entzündung zurückzuführen ist (BASF SE 2019 a). Die Adversität der Befunde am Larynx bei 0,1 mg/m^3^ ist fraglich, da sie in der Ausprägung Grad 1 haben (Kaufmann et al. 2009). Die Befunde in der Lunge bei 0,1 mg/m^3^ (Ansammlung von Makrophagen) sind vermutlich eine physiologische Reaktion und daher ebenfalls von fraglicher Adversität. Da die bei der niedrigsten Konzentration von 0,02 mg Al/m^3^ bei männlichen und weiblichen Tieren erhöhte ALP in der BALF ein Zeichen von geschädigten Pneumozyten ist (Schildge et al. 2000 und erste Zeichen histopathologischer Befunde zu beobachten sind, wird diese Konzentration von der Kommission für beide Geschlechter als LOAEC betrachtet.

Zu der 13-Wochen-Inhalationsstudie liegt eine 14-tägige Vorstudie in Übereinstimmung mit OECD-Prüfrichtlinie 412 an männlichen Wistar-Ratten vor. Gruppen von je fünf männlichen Tieren wurden sechs Stunden pro Tag, fünf Tage pro Woche gegen Konzentrationen von 0, 1, 5 oder 25 mg/m^3^ (0,2; 1; 5 mg Al/m^3^) eines Aerosols von Aluminiumchlorid in einer wässrigen Lösung (6%ig) exponiert. Tägliche klinische Beobachtungen und Körpergewichte wurden aufgezeichnet. Am Ende der Studie wurden Blut, BALF und Atemwege untersucht. Ab der niedrigsten Konzentration traten in der BALF ein Anstieg der Gesamtzellzahl, der absoluten und relativen Neutrophilen sowie der relativen Monozyten auf. Der Anteil an Makrophagen in der BALF war reduziert, Gesamtprotein und Enzymaktivitäten der LDH, β-*N*-Acetylglucosaminidase (NAG) und GGT in der BALF erhöht. In der Nasenhöhle (Schnittebene IV) traten minimale Erosionen/Ulzera bei einem der fünf Tiere auf (BASF SE 2019 b). Weitere Effekte bei höheren Konzentrationen siehe Tabelle 8.

Ein Vergleich der Ergebnisse der 13-Wochen-Inhalationsstudie (Aerosol aus 0,2%iger AlCl_3_-Lösung; BASF SE 2019 a) und der 14-Tage-Vorstudie (Aerosol aus 6%iger AlCl_3_-Lösung; BASF SE 2019 b) zeigt, dass die Wirkstärke mit der Konzentration von Aluminiumchlorid in der Aerosol-Lösung zunimmt, siehe Tabelle 8 und Tabelle 9.

**Tab. 9:** Vergleich der BALF-Befunde der 13-Wochen-Inhalationsstudie (Aerosol aus 0,2%iger AlCl_3_-Lösung) (BASF SE 2019 a) mit der 14-Tage-Vorstudie (Aerosol aus 6%iger Lösung) (BASF SE 2019 b)

**Exposition**	**Konzentrationen in der BALF der männlichen Tiere**						
	**Neutrophile [Zahl/µl]**	**Gesamtzellzahl [Zahl/µl]**	**Monozyten [Zahl/µl]**	**Gesamtprotein [mg/l]**	**LDH [µkat/l]**	**GGT [nkat/l]**	**NAG [nkat/l]**
0,5 mg/m^3^, 13 Wo	38,97	82,57	0,06	58	0,62	68	0,74
1 mg/m^3^, 2 Wo	43,26	93,53	1,02	62	1,07	85	1,00
2 mg/m^3^, 13 Wo	161,58	195,54	0,46	101	1,46	104	0,92

GGT: γ-Glutamyltransferase; LDH: Lactatdehydrogenase; NAG: β-*N*-Acetylglucosaminidase; Wo: Wochen

In einer Untersuchung an männlichen Sprague-Dawley-Ratten wurde Aluminiumchlorid (parallel auch Aluminiumfluorid) in nur einer Expositionskonzentration von 1,28 mg/m^3^ eingesetzt, die zu deutlichen Effekten in der Lunge führte. Neben dem Körpergewicht und den Organgewichten von Nieren, Leber, Lunge und Gehirn wurde nur eine ausgewählte Anzahl von Parametern (BALF: Lysozym, Glucose-6-phosphat-Dehydrogenase, Protein, ALP, Makrophagenanzahl und -vitalität) bestimmt, von denen die Autoren annahmen, dass sie frühe Warnzeichen für pathologische Effekte sind. In der 13. und 18. Studienwoche wurden Zunahmen des relativen Leber- und/oder Nierengewichts um etwa 10 % beobachtet (Finelli et al. 1981), jedoch sind die Veränderungen des Leber- und Nierengewichtes nicht spezifisch der Aluminiumfluorid- oder Aluminiumchloridgruppe zugeordnet, so dass sie nicht zur Bewertung herangezogen werden können. Eine histopathologische Untersuchung wurde nicht durchgeführt.

Sechsmonatige Inhalationsstudien an männlichen und weiblichen F344-Ratten und Hartley-Meerschweinchen führten bei 0,25 mg Aluminiumchlorhydrat-Staub/m^3^ zu Mikrogranulomen in den peribronchialen Lymphknoten bei Ratten, und einem leichten Anstieg alveolärer Makrophagen in der Lunge von Ratten und Meerschweinchen. Die nächsthöhere Konzentration von 2,5 mg/m^3 ^führte zu deutlichen Befunden an der Lunge beider Spezies (Steinhagen et al. 1978; Stone et al. 1979). Die mit dem am Auge allenfalls leicht reizend wirkenden Aluminiumchlorhydrat bei 0,25 mg/m^3^ beobachtete Makrophagenansammlung in der Lunge ist per se nicht als advers zu betrachten. Mikrogranulome können jedoch als Zeichen einer spezifischen Entzündung gewertet werden (Pagán und Ramakrishnan 2018) und treten bei 0,25 mg/m^3^ bei einer von zehn Ratten und in den peribronchialen Lymphknoten auf. Bei der nächsthöheren Konzentration sind Mikrogranulome in den peribronchialen Lymphknoten bei allen zehn Tieren zu finden, so dass dieser Effekt von der Kommission als pathologische Reaktion und als ein bei 0,25 mg/m^3^ beginnender Effekt gewertet wird. Die Konzentration von 0,25 mg/m^3^ der Studie von Steinhagen et al. (1978) ist somit eine LOAEC.

**Fazit:** Die niedrigste LOAEC für lösliche Aluminiumverbindungen resultiert aus der 13-wöchigen Inhalationsstudie nach OECD-Prüfrichtlinie 413 an Wistar-Ratten mit dem ätzend wirkenden Aluminiumchlorid (siehe Abschnitt 2.1) und beträgt 0,1 mg AlCl_3_/m^3^ (0,02 mg Al/m^3^). Bei dieser Konzentration zeigten erhöhte ALP-Werte und Neutrophile in der BALF eine beginnende Lungentoxizität an. Der Vergleich der 13-Wochen-Inhalationsstudie mit der 14-Tage-Vorstudie ergibt eine Zunahme der Wirkstärke mit der Konzentration von Aluminiumchlorid in der zur Erzeugung des Aerosols verwendeten Aluminiumchlorid-Lösung.

Mit dem am Auge allenfalls schwach reizend wirkenden Aluminiumchlorhydrat (siehe Abschnitt 2.1) kann aus einer sechsmonatigen Inhalationsstudie mit F344-Ratten eine LOAEC von 0,25 mg/m^3^ (ca. 0,08 mg Al/m^3^) abgeleitet werden, bei der ebenfalls eine beginnende Lungentoxizität zu beobachten ist.

##### 5.2.1.2 Wirkungen auf das ZNS 

Hierzu liegen keine spezifischen Untersuchungen vor.

Die Untersuchung des Verhaltens (FOB: functional observational battery) der Wistar-Ratten in der oben berichteten 13-Wochen-Inhalationsstudie nach OECD-Prüfrichtlinie 413 ergab keinen Effekt (BASF SE 2019 a). Eine FOB ist eine reine Verhaltensbeobachtung und keine gezielte Testung bestimmter kognitiver oder motorischer Funktionen, wie sie in den epidemiologischen Untersuchungen durchgeführt wurden (siehe Abschnitt 4.2).

Bei F344-Ratten und Hartley-Meerschweinchen, die sechs Monate lang gegen bis zu 6,1 mg Al/m^3^ als Aluminiumchlorhydrat-Staub ausgesetzt waren, wurden keine Veränderungen des Gehirngewichts oder in der Histologie festgestellt (Steinhagen et al. 1978). Bei Sprague-Dawley-Ratten, die bis zu 26 Wochen lang gegen 0,37 mg Al/m^3^ als Aluminiumchlorid exponiert worden waren, wurden keine Auswirkungen auf das Gehirngewicht festgestellt. Eine histopathologische Untersuchung fand nicht statt (Finelli et al. 1981).

**Fazit:** Es liegen keine Studien vor, um die neurotoxische Wirkung am Tier nach inhalativer Exposition löslicher Aluminiumverbindungen abschließend bewerten zu können.

#### 5.2.2 Orale Aufnahme

Seit dem Nachtrag (Greim 2007) sind zahlreiche neue Studien mit oraler wiederholter Gabe von löslichen Aluminiumverbindungen hinzugekommen.

##### 5.2.2.1 Neurotoxizität

Der systematische Review von Willhite et al. (2014) fasst zusammen, dass in keiner der neueren Tierstudien, in denen die orale Verabreichung von Aluminiumsalzen untersucht wurde, eindeutige Hinweise auf neurologische Schäden ohne gleichzeitige systemische Toxizität vorliegen.

Der Endpunkt Neurotoxizität nach oraler Gabe wird hier nicht erneut abgehandelt, da die neurotoxische Wirkung beim Menschen nach inhalativer Exposition am Arbeitsplatz bekannt ist (Hartwig und MAK Commission 2025) und erst bei Konzentrationen oberhalb der für die löslichen Aluminiumverbindungen beobachteten Effekte an der Lunge von Tieren (siehe Abschnitt 5.2.1.1) auftreten. Zum Schutz vor neurotoxischer Wirkung ist der BAT-Wert von 50 µg/g Kreatinin (Klotz et al. 2018) einzuhalten.

##### 5.2.2.2 Weitere systemische Toxizität

Die in Greim (2007) für die systemische Toxizität von löslichen Aluminiumverbindungen ermittelten NOAEL betrugen für Ratten 52 mg Al/kg KG und Tag (LOAEL 260 mg/kg KG und Tag, 100 Tage) und für Beagle-Hunde 24 mg/kg KG und Tag (LOAEL 77 mg/kg KG und Tag, sechs Monate). Für Neuseeländer-Kaninchen wird ein LOAEL von 6,8 mg Al/kg KG und Tag (16 Wochen) beschrieben. Es traten vor allem erniedrigte Körpergewichte und Effekte auf Leber und Nieren auf.

Aufgrund der für den Arbeitsplatz geringeren Relevanz oraler Studien werden im Folgenden nur die Studien zu den in Tabelle 1 aufgeführten Aluminiumverbindungen beschrieben, die nach aktuellen Prüfrichtlinien durchgeführt wurden.

Die Studien sind in Tabelle 10 zusammengefasst.

**Tab. 10:** Toxizität von löslichen Aluminiumverbindungen nach wiederholter oraler Verabreichung

**Spezies, ** **Stamm, ** **Anzahl pro Gruppe**	**Exposition**	**Befunde^a)^**	**Literatur**
**Ratte**, Wistar Crl: (WI) BR, 10 ♂, 10 ♀	**Screening-Test zur Reproduktions- u. Entwicklungstoxizität**, **2 Wo vor Verpaarung, während Verpaarung (**♂, ♀**) bis mindestens zum 3. PND (**♀**)**, Expositionsdauer: ♂: 4 Wo, ♀: 37‑53 d, **Al-Chlorid, basisch, Al(OH)_1,3_Cl_1,7_ (109,36 g/mol) in wässriger Lösung, ** 0, 40, 200, 1000 mg Lösung/kg KG u. d (gemessen: 0; 3,4; 18,4; 92,8 mg Al/kg KG u. d), Gavage, 7 d/Wo, Vehikel: Wasser, **OECD TG 422**	**18,4 mg Al/kg KG: NOAEL lokale Reizeffekte (Magen);** **92,8 mg Al/kg KG: NOAEL systemische Effekte** ♂/♀: Drüsenmagen: minimale, leichte bis mäßige subakute Entzündung, minimale bis mäßige eosinophile Sphäroide in der äußeren Mucosa (degenerative Produkte, die möglicherweise mit der Entzündung unterhalb der Mucosabasis assoziiert sind), Blut: MCHC ↓ (♂: 2 %, ♀: 3,3 %), ♂: Blut: Thrombozytenzahl ↑ (27 %), ♀: KG ↓ (2.–4. Behandlungs-Wo, KG-Zunahme nicht betroffen)	NOTOX B.V. 2007 a
**Ratte**, Sprague Dawley, je 20 ♀	**GD 6–PND 364**, **Aluminiumcitrat,** pH-Wert der Al-Lösung zwischen 6 u. 7 eingestellt, 0, 30, 100, 300 mg Al/kg KG u. d, Trinkwasser, gemessene Dosis anhand Trinkwasserverbrauch: während Gestation: 25,9–27,0; 94,3–102,0; 195,4–199,9 mg/kg KG u. d, während Laktation: 28,6–44,5; 99,9–165,2; 298,6–523,3 mg/kg KG u. d, Kontrolle: unbehandeltes Trinkwasser oder Natriumcitrat (27,2 g/l Trinkwasser), **OECD TG 426 u. OECD TG 452**	**102,0 mg Al/kg KG: NOAEL** **bei 199,9 mg Al/kg KG**: Diarrhoe	Poirier et al. 2011; ToxTest Alberta Research Council Inc. 2010

^a)^ wenn nicht anders angegeben, sind die aufgeführten Veränderungen statistisch signifikant

Al: Aluminium; d: Tage; FOB: functional observational battery; GD: Gestationstag; MCHC: mittlere korpuskuläre Hämoglobin-Konzentration; PND: Postnataltag; TG: Test Guideline (Prüfrichtlinie); Wo: Woche(n)

#### 5.2.3 Dermale Aufnahme

Hierzu liegen keine validen Untersuchungen vor.

### 5.3 Wirkung auf Haut und Schleimhäute

#### 5.3.1 Haut

Aufgrund der Vielzahl erschienener Publikationen werden im Folgenden nur die Studien zu den in Tabelle 1 beschriebenen Aluminiumverbindungen, die nach aktuellen Prüfrichtlinien durchgeführt wurden, dargestellt (Testbedingungen und Ergebnisse siehe Tabelle 11).

**Tab. 11:** Studien mit löslichen Aluminiumverbindungen zur Reizwirkung an der Haut

**Testsubstanz**	**Testsystem, Anzahl, Dauer**	**Dosis**	**Effekte**	**Bewertung **	**Literatur**
Aluminiumammoniumdisulfat	Neuseeländer-Kaninchen, 3 ♂, 4 h, semiokklusiv, Ablesezeitpunkte: 1, 24, 48, 72 h, OECD TG 404	0,5 g (Pulver, angefeuchtet mit Wasser)	PII: 0 von 8, sehr leichte Erytheme bei einem Tier nach 4 h, reversibel nach 24 h	nicht reizend	ECHA 2020 a
	Humanhaut, EpiDerm^TM^ SIT (EPI-200)-Modell, 3 Replikate, Exposition: 60 min, Ablesezeitpunkt: 42 h, OECD TG 439	0,025 g	%-Gewebelebensfähigkeit im Mittel: 96,9	nicht reizend	
Aluminiumchlorhydrat	Neuseeländer-Kaninchen, 3 ♂, 4 h, semiokklusiv, Ablesezeitpunkte: 30 min, 1, 24, 48, 72 h, OECD TG 404	0,5 g (Pulver, angefeuchtet mit 0,9%iger NaCl-Lösung)	PII: 0 von 8, o. B.	nicht reizend	ECHA 2022 c
Aluminiumchlorid	Humanhaut, Corrositex® Biobarrier Membrane-Modell, 4 Replikate, OECD TG 435	0,5 g	mittlere Durchbruchszeit: 16,87 min	ätzendes Potenzial	BASF SE 2015; ECHA 2022 a
Aluminiumchlorid, basisch	Neuseeländer-Kaninchen, 3 (k. w. A.), 4 h, semiokklusiv, Ablesezeitpunkte: 30 min, 1, 24, 48, 72 h, OECD TG 404	0,5 ml (35%ig, Rest der Lösung unbekannt)	PII: 0 von 8, o. B.	nicht reizend	ECHA 2023 a
Aluminiumchloridhydroxysulfat	Neuseeländer-Kaninchen, 3 ♂, 4 h, semiokklusiv, Ablesezeitpunkte: 1, 24, 48, 72 h, OECD TG 404	0,5 ml	PII: 0 von 8, sehr leichte Erytheme bei einem Tier nach 4 h, reversibel nach 24 h	nicht reizend	ECHA 2020 b
Aluminiumdiacetat	Humanhaut, RHE^TM^-Modell, 3 Replikate, Exposition: 42 min, Ablesezeitpunkt: 42 h, OECD TG 439	0,016 g	%-Gewebelebensfähigkeit: 93,5; 94,3; 93,9	nicht reizend	ECHA 2020 c
Aluminiumlactat	Humanhaut, EPISKIN Small-Model^TM^, 3 Replikate, Exposition: 15 min, Ablesezeitpunkt: 44 h, OECD TG 439	0,010–0,012 g in 5 µl Wasser	%-Gewebelebensfähigkeit im Mittel: 103	nicht reizend	ECHA 2022 b
Aluminiumnitrat	Neuseeländer-Kaninchen, 3 ♂, 4 h, semiokklusiv, k. w. A., OECD TG 404	0,5 g angeteigt (k. w. A.)	PII: 0,35 von 8, Effekte vollständig reversibel	nicht bis leicht reizend	ECHA 2023 b
Aluminiumsulfat	Neuseeländer-Kaninchen, 1 ♂, 2 ♀, 4 h, semiokklusiv, Ablesezeitpunkte: 1, 24, 48, 72 h, OECD TG 404	0,5 g angefeuchtet (mit 0,5 ml Wasser)	PII: 0 von 8, o. B.	nicht reizend	ECHA 2023 c
	Neuseeländer-Kaninchen, 3 ♂, 4 h, semiokklusiv, Ablesezeitpunkte: 1, 24, 48, 72 h, OECD TG 404	0,5 ml	PII: 0 von 8, sehr leichte Erytheme bei zwei Tieren nach 4 h, reversibel nach 24 h	nicht reizend	

h: Stunden; k. w. A.: keine weiteren Angaben; o. B.: ohne Befund; PII: Primärer Irritationsindex = (∑ Erythem 24/48/72 h + ∑ Ödem 24/48/72 h)/3 × Anzahl der Tiere; TG: Test Guideline (Prüfrichtlinie)

**Fazit:** Die verschiedenen löslichen Aluminiumverbindungen sind von nicht reizend (Aluminiumammoniumdisulfat, -chlorhydrat, Aluminiumchlorid, basisch (35%ig), Aluminiumchloridhydroxysulfat, -diacetat, -lactat, -sulfat) bis ätzend (Aluminiumchlorid) an der Haut von Kaninchen oder an Humanhaut-Modellen bewertet worden.

#### 5.3.2 Auge

Aufgrund der Vielzahl erschienener Publikationen werden im Folgenden nur die Studien zu den Aluminiumverbindungen aus Tabelle 1 beschrieben, die nach aktuellen Prüfrichtlinien durchgeführt wurden. Die Testbedingungen, Ergebnisse und Einstufungen nach CLP sind in Tabelle 12 dargestellt.

**Tab. 12:** Studien mit löslichen Aluminiumverbindungen zur Reizwirkung am Auge

**Testsubstanz**	**Testsystem, Anzahl, Dauer **	**Dosis**	**Effekte**	**CLP-Einstufung**	**Literatur**
Aluminiumammoniumdisulfat	Neuseeländer-Kaninchen, 3 ♂, Ablesezeitpunkte: 1, 24, 48, 72 h, Nachbeobachtung bis 21 d, OECD TG 405	100 mg	Reizwerte der Einzeltiere (24–72 h): Cornea-Trübung 0 von max. 4, Iris 0 von max. 2, Bindehäute: Erythem 0,67–1 von max. 3, Schwellung 0,33–0,67 von max. 4, Effekte bei 3/3 nach 72 h reversibel	nicht reizend	ECHA 2020 a
	EpiOcular^TM ^EIT(OCL-200)-Modell, 3 Replikate, Exposition: 6 h, Ablesezeitpunkt: 18 h, OECD TG 492	50 mg	% mittlere Gewebelebensfähigkeit: 67,9		
Aluminiumchlorhydrat	Neuseeländer-Kaninchen, 3 ♂, Ablesezeitpunkte: 1, 24, 48, 72 h, Nachbeobachtung bis 21 d, OECD TG 405	100 mg	Reizwerte der Einzeltiere (24–72 h): Cornea-Trübung 0 von max. 4, Iris 0 von max. 2, Bindehäute: Erythem 2 von max. 3 (3/3 Tieren), Schwellung 0 von max. 4, Effekte bei 3/3 nach 72 h reversibel	nicht reizend	Aventis Pharma Deutschland GmbH 2010; ECHA 2022 c
Aluminiumchloridhydroxysulfat	Neuseeländer-Kaninchen, 3 ♂, Ablesezeitpunkte: 1, 24, 48, 72 h, Nachbeobachtung bis 21 d, OECD TG 405	0,1 ml	Reizwerte der Einzeltiere (24–72 h): Cornea-Trübung 0 von max. 4, Iris 0 von max. 2, Bindehäute: Erythem 1,0–2,0 von max. 3, Ausfluss (24 und 48 h; k. w. A.), Schwellung 0,7–1,0 von max. 4, Effekte bei 3/3 nach bis 6 d reversibel	augenreizend, Kategorie 2	ECHA 2020 b
Aluminiumdiacetat	BOCP-Test, 3 Replikate, Ablesezeitpunkt: 240 min, OECD TG 437	150 mg in 0,75 ml 0,9%iger NaCl-Lösung	Reizwerte (Scores), IVIS: 146,9; 173,9; 182,9	irreversible Wirkungen am Auge, Kategorie 1	ECHA 2020 c
Aluminiumlactat	Neuseeländer-Kaninchen, 3 ♂, Ablesezeitpunkte: 1, 24, 48, 72 h, Nachbeobachtung bis 6 d, OECD TG 405	0,01 ml (6 mg)	Reizwerte der Einzeltiere (24–72 h): Cornea-Trübung 0–0,3 von max. 4, Iris 0–0,3 von max. 2, Bindehäute: Erythem 2,0 von max. 3, Ausfluss (24–78 h), Schwellung 1,3 von max. 4, Effekte nach 6 d reversibel	augenreizend, Kategorie 2	ECHA 2022 b
Aluminiumnitrat	Neuseeländer-Kaninchen, 3 ♂, Ablesezeitpunkte: 1, 24, 72 h, Nachbeobachtung bis 6 d, OECD TG 405	100 mg	Reizwerte nach OECD nicht angegeben – starke Reizung nach 7 d nicht reversibel bei Augen, die nicht gewaschen oder nach 4 sec gespült wurden	irreversible Wirkungen am Auge, Kategorie 1	ECHA 2023 b
Aluminiumsulfat	Neuseeländer-Kaninchen, 1 ♂, Ablesezeitpunkte: 1, 24, 48 h, OECD TG 405	100 mg	Reizwerte (24–48 h): Cornea-Trübung 2 von max. 4, Verschorfung der Cornea (24–48 h), Iris 1 von max. 2, Bindehäute: Erythem 2 von max. 3, Verschorfung der Binde- und Nickhaut (48 h), Schwellung 3 von max. 4, Versuch nach 48 h abgebrochen wegen starker Effekte	irreversible Wirkungen am Auge, Kategorie 1	ECHA 2023 c
	Neuseeländer-Kaninchen, 3 ♂, Ablesezeitpunkte: 1, 24, 48, 72 h, Nachbeobachtung bis 14 d, OECD TG 405	0,1 ml	Reizwerte der Einzeltiere (24–72 h): Cornea-Trübung 0 von max. 4, Iris 1 von max. 2, Bindehäute: Erythem 0–2 von max. 3, Chemosis 1–2 von max. 4, Effekte nach 7 d reversibel		

BOCP-Test: Test mit Rinderaugen auf Opazität und Permeabilität (Bovine Corneal Opacity-Permeability); d: Tage; h: Stunde(n); IVIS: In vitro irritancy score; TG: Test Guideline (Prüfrichtlinie)

**Fazit:** Die Aluminiumverbindungen wurden anhand von Untersuchungen am Auge von Kaninchen und in vitro von nicht reizend (Aluminiumchlorhydrat und Aluminiumammoniumdisulfat) bis zu irreversibel augenschädigend (Aluminiumchloridhydroxysulfat, Aluminiumdiacetat, Aluminiumlactat, Aluminiumnitrat, Aluminiumsulfat) bewertet. Für das an der Haut stark reizend bis ätzend wirkende Aluminiumchlorid wurde keine Studie durchgeführt, da die verfügbaren Informationen anzeigen, dass die Kriterien für eine Einstufung in die Kategorie 1 (irreversible Wirkungen am Auge) nach CLP erfüllt sind (ECHA 2022 a).

### 5.4 Allergene Wirkung

#### 5.4.1 Hautsensibilisierende Wirkung

Im Nachtrag (Greim 2007) wurden ein Landsteiner-Draize-Test, ein Maximierungstest sowie ein Local Lymph Node Assay mit Aluminiumchlorid beschrieben. Alle drei Tests lieferten ein negatives Testergebnis. Neue nachfolgend aufgeführte tierexperimentelle Untersuchungen ergaben ebenfalls negative Ergebnisse.

Ein Maximierungstest mit Dialuminiumchloridpentahydroxid, gleichwertig mit OECD-Prüfrichtlinie 406, verlief an zehn getesteten Pirbright-White-Meerschweinchen negativ. Die intradermale Induktion erfolgte mit einer 0,2%igen wässrigen Lösung, die dermale Induktion und Provokation erfolgte mit einer 25%igen wässrigen Lösung. Da bei der intradermalen Induktionsbehandlung bereits starke Irritationen auftraten, wurde vor der dermalen Applikation auf die Vorbehandlung mit Natriumlaurylsulfat verzichtet. Bei der dermalen Induktion wurden starke Ödeme, Nekrosen, offene Wunden an den Stellen, welche zuvor mit FCA behandelt worden waren, beobachtet. Bei der Provokation zeigte keines der Tiere eine positive Hautreaktion (Hoechst Marion Roussel Deutschland GmbH o.J.).

Auch ein Maximierungstest mit Aluminiumchlorhydrat nach OECD-Prüfrichtlinie 406 an 20 Dunkin-Hartley-Meerschweinchen war negativ. Die intradermale Induktion erfolgte mit 0,1%iger Testzubereitung, die epikutane Induktion sowie die Provokation erfolgten mit der 50%igen (G/G) Testzubereitung in Wasser. Es wurden keine Hautreaktionen beobachtet (ECHA 2023 a).

Ein Maximierungstest mit Aluminiumdiacetat nach OECD-Prüfrichtlinie 406 an 20 Dunkin-Hartley-Meerschweinchen verlief ebenfalls negativ. Die intradermale Induktion erfolgte mit 5%iger wässriger Testzubereitung, die epikutane Induktion sowie die Provokation erfolgten mit der 40%igen (G/G) Testzubereitung. Es wurden keine Hautreaktionen beobachtet (ECHA 2020 c). Angaben über eine Positivkontrolle fehlen.

Ein Local Lymph Node Assay (LLNA) mit Aluminiumsulfat nach OECD-Prüfrichtlinie 429 mit fünf CBA/J-Mäusen pro Behandlungsgruppe zeigte ein negatives Ergebnis. Mit den Testkonzentrationen 10, 25 und 50 % (Testzubereitung in 1 % wässriger Pluronic L02-Lösung) wurden Stimulationsindices von 0,9; 1,6 und 0,5 beobachtet (ECHA 2023 c).

Ein weiterer LLNA mit Aluminiumammoniumdisulfat nach OECD-Prüfrichtlinie 429 mit vier CBA/J-Mäusen pro Behandlungsgruppe verlief ebenfalls negativ. Mit den Testkonzentrationen 10, 25 und 50 % (Testzubereitung in Propylenglykol) wurden Stimulationsindices von 0,8; 1,5 und 1,1 beobachtet. Insgesamt ist der LLNA jedoch wenig geeignet, um das sensibilisierende Potenzial von Metallen zu erfassen (ICCVAM 2009).

Es liegen keine Untersuchungen mit tierversuchsfreien Alternativverfahren vor.

#### 5.4.2 Atemwegssensibilisierende Wirkung

Es steht kein validiertes Tiermodell zur Verfügung.

### 5.5 Reproduktionstoxizität

Detaillierte Zusammenstellungen von Daten zur Reproduktionstoxizität von Aluminiumverbindungen sind in einem Übersichtsartikel (Yokel 2020) und in den Berichten der ATSDR und der EFSA zu finden (ATSDR 2008; EFSA 2008).

#### 5.5.1 Fertilität

##### 5.5.1.1 In vivo

Aus einer Ein-Generationen-Studie an Sprague-Dawley-Ratten mit Schlundsondengabe lässt sich bis zur höchsten Dosis von 52 mg Al/kg KG und Tag, verabreicht als Aluminiumnitrat, kein Hinweis auf eine Störung der Fertilität ableiten. Bei männlichen Ratten, Mäusen und Kaninchen treten verringerte Gewichte der Reproduktionsorgane nur bei Dosierungen auf, die zu einer Erniedrigung der Körpergewichte führen (Greim 2007).

In Tabelle 13 sind die neu hinzugekommenen Generationenstudien und Studien zur Fertilität von löslichen Aluminiumverbindungen zusammengestellt. Studien mit einmaliger i.p. Gabe, ohne histologische Untersuchung oder einer geringeren Tierzahl als fünf pro Gruppe wurden nicht aufgenommen.

In einer kombinierten Studie nach OECD-Prüfrichtlinie 422 mit oraler wiederholter Verabreichung und Screening-Test auf Reproduktions-/Entwicklungstoxizität an männlichen und weiblichen Wistar Crl: (WI) BR-Ratten kam es bis zur höchsten Dosis von 1000 mg einer Lösung von basischem Aluminiumchlorid/kg KG und Tag (gemessen: 92,8 mg Al/kg KG und Tag) nicht zu Effekten auf die Fertilität und auf die männlichen und weiblichen Reproduktionsorgane. Der NOAEL für Fertilität und für systemische Toxizität lag bei der höchsten Dosis von 92,8 mg Al/kg KG und Tag (NOTOX B.V. 2007 a).

In einer 2-Generationen-Studie nach OECD-Prüfrichtlinie 416 an Crl:CD(SD)-Ratten mit Trinkwassergabe traten bis zur höchsten Konzentration von 3000 mg Aluminiumsulfat/l Trinkwasser (F0 ♂: 31,2; ♀: 52,0 mg Al/kg KG und Tag) keine Effekte auf Fertilität sowie männliche und weibliche Reproduktionsorgane auf. Ab der niedrigsten Konzentration von 120 mg Aluminiumsulfat/l Trinkwasser (F0 ♂: 2,96; ♀: 4,50 mg Al/kg KG und Tag) kam es zu einer Abnahme der Trinkwasseraufnahme vermutlich wegen der durch die Azidität von Aluminiumsulfat (Trinkwasser-pH-Wert 3,57–4,20) verminderten Palatabilität. Der NOAEL für Parentaltoxizität lag bei 600 mg Aluminiumsulfat/l Wasser (F0 ♂: 8,06; ♀: 13,5 mg Al/kg KG und Tag) (Hirata-Koizumi et al. 2011 b). Der NOAEL für Fertilität betrug 3000 mg Aluminiumsulfat/l Wasser (F0 ♂: 31,2; ♀: 52,0 mg Al/kg KG und Tag), was der höchsten Dosis entspricht.

Dieselbe Arbeitsgruppe führte eine weitere 2-Generationen-Studie nach OECD-Prüfrichtlinie 416 mit 0, 50, 500 oder 5000 mg Aluminiumammoniumsulfat/l Trinkwasser durch. Es kam mit dieser Testsubstanz ebenfalls zu einer erniedrigten Trinkwasseraufnahme, die die Autoren auf die adstringierende Wirkung zurückführten. Der pH-Wert des Aluminiumsulfat-haltigen Trinkwassers ist nicht angegeben. Es ergab sich ein NOAEL für Parentaltoxizität und Fertilität von 5000 mg Aluminiumammoniumsulfat/l Wasser (F0 ♂: 36,3; ♀: 59,0 mg Al/kg KG und Tag), der höchsten Konzentration (Hirata-Koizumi et al. 2011 a).

**Tab. 13:** Generationenstudien und Studien zur Fertilität von löslichen Aluminiumverbindungen

**Spezies, Stamm, Anzahl pro Gruppe**	**Exposition**	**Befunde^a)^**	**Literatur**
**Ratte**, Wistar Crl: (WI) BR, 10 ♂, 10 ♀	**Screening-Test zur Reproduktions- u. Entwicklungstoxizität**, **2 Wo vor Verpaarung, während Verpaarung (**♂, ♀**) bis mindestens zum 3. PND (**♀**)**, Expositionsdauer: ♂: 4 Wo, ♀: 37–53 d, **Al-Chlorid, basisch, Al(OH)_1,3_Cl_1,7_ (109,36 g/mol) in wässriger Lösung**, 0, 40, 200, 1000 mg Lösung/kg KG u. d (gemessen: 0; 3,4; 18,4; 92,8 mg Al/kg KG u. d), Gavage, 7 d/Wo, Vehikel: Wasser, **OECD TG 422**	**18,4 mg Al/kg KG: NOAEL lokale Reizeffekte Magen**; **92,8 mg Al/kg KG: NOAEL systemische Effekte, Effekte auf die Fertilität, perinatale Toxizität**; siehe auch Abschnitt 5.2.2	NOTOX B.V. 2007 a
**Ratte**, Crl:CD(SD), 24 ♂, 24 ♀	**2-Generationen-Studie**, **10 Wo vor Verpaarung bis 3 Wo nach Entwöhnung**, **Al_2_(SO_4_)_3_**, 0, 120, 600, 3000 mg/l Trinkwasser (basierend auf Al im Trinkwasser u. Futter: F0 ♂: 1,62; 2,96; 8,06; 31,2 mg Al/kg KG u. d; F0 ♀: 2,29; 4,50; 13,5; 52,0 mg Al/kg KG u. d; F1 ♂: 1,93; 3,55; 9,78; 38,5 mg Al/kg KG u. d; F1 ♀: 2,35; 4,72; 14,0; 55,6 mg Al/kg KG u. d), Trinkwasser, 7 d/Wo, **OECD TG 416**	**ab 120 mg Al-Sulfat/l (F0 ♂: 2,96/♀: 4,50 mg Al/kg KG)**: F0: Wasseraufnahme ↓ (♂/♀: gesamte Behandlungszeit), F1-Adulte: Wasseraufnahme ↓ (♂: 3.–6., 8., 10. Behandlungs-Wo); **600 mg Al-Sulfat/l (F0 ♂: 8,06/♀: 13,5 mg Al/kg KG): NOAEL für Parentaltoxizität**; **ab 600 mg Al-Sulfat/l**: F0: Futteraufnahme ↓ (♂: 1. Behandlungs-Wo, ♀: 3. Postnatal-Wo), Nebenhoden: Spermienzahl ↓ (253,8 ± 61,3 × 10^6^/ Nebenhodenschwanz; Kontrolle: 286,3 ± 40,3 × 10^6^/ Nebenhodenschwanz, nicht in der F1-Generation), F1-Adulte: Wasseraufnahme ↓ (♂: gesamte Behandlungszeit, ♀: 3. Postnatal-Wo), Futteraufnahme ↓ (♂: 10. Behandlungs-Wo, ♀: 3. Postnatal-Wo); **3000 mg Al-Sulfat/l (F0 ♂: 31,2/♀: 52,0 mg Al/kg KG): NOAEL für Effekte auf die Fertilität u. perinatale Toxizität**; **bei 3000 mg Al-Sulfat/l**: F0: Futteraufnahme ↓ (♂: 8., 13.–14. Behandlungs-Wo, ♀: 1. Behandlungs-Wo), KG ↓ (♂: 1.–3. Behandlungs-Wo, ♀: 1.–2 Behandlungs-Wo), abs. u. rel. Lebergewicht ↓ (♂), F1-Jungtiere: KG ↓ (♂/♀: PND 21), Vaginalöffnung verzögert (Pubertätseintritt ♀: d 31,4 ± 1,7, Kontrolle: d 29,5 ± 2,1), abs. u. rel. Lebergewicht ↓ (♂/♀: PND 21), abs. u. rel. Milzgewicht ↓ (♂: PND 21), F1-Adulte: Wasseraufnahme ↓ (♀: gesamte Behandlungszeit), F2-Jungtiere: KG ↓ (♀: PND 21), abs. u. rel. Lebergewicht ↓ (♀: PND 21), abs. u. rel. Thymusgewicht ↓ (♀: PND 21), abs. u. rel. Milzgewicht ↓ (♂: PND 21)	Hirata-Koizumi et al. 2011 b
**Ratte**, Crl:CD(SD), 24 ♂, 24 ♀	**2-Generationen-Studie**, **10 Wo vor Verpaarung bis 3 Wo nach Entwöhnung**, **AlNH_4_(SO_4_)_2_**, 0, 50, 500, 5000 mg/l Trinkwasser (basierend auf Al im Trinkwasser u. Futter: F0 ♂: 1,56; 1,98; 5,35; 36,3 mg Al/kg KG u. d; F0 ♀: 2,20; 2,89; 8,81; 59,0 mg Al/kg KG u. d; F1 ♂: 1,83; 2,35; 6,57; 44,2 mg Al/kg KG u. d; F1 ♀: 2,39; 3,10; 9,36; 61,1 mg Al/kg KG u. d), 7 d/Wo, **OECD TG 416**	**ab 50 mg Al-Ammoniumsulfat/l**: F0: Wasseraufnahme ↓ (♂: gesamte Behandlungszeit, ♀: 4. u. 8.–10. Behandlungs-Wo), F1-Adulte: Wasseraufnahme ↓ (♀: 1., 9., 10. Behandlungs-Wo, 1. Gestations-Wo, 1. Postnatal-Wo); **ab 500 mg Al-Ammoniumsulfat/l**: F0: Wasseraufnahme ↓ (♀: gesamte Behandlungszeit), Futteraufnahme ↓ (♀: 1. Behandlungs-Wo), F1-Adulte: Wasseraufnahme ↓ (♂/♀: gesamte Behandlungszeit); **5000 mg Al-Ammoniumsulfat/l (F0 ♂: 36,3/♀: 59,0 mg Al/kg KG): NOAEL für Parentaltoxizität, Effekte auf die Fertilität u. perinatale Toxizität**; **bei 5000 mg Al-Ammoniumsulfat/l Wasser**: F0: Futteraufnahme ↓ (♂: 1. Behandlungs-Wo, ♀: 2. u. 3. Postnatal-Wo), KG ↓ (♂: 2. Behandlungs-Wo, ♀: 2. Behandlungs-Wo u. 21. PND), F1-Jungtiere: Vaginalöffnung verzögert (Pubertätseintritt ♀: d 32,3 ± 1,8, Kontrolle: d 30,2 ± 2,1), abs. u. rel. Lebergewicht ↓ (♂: PND 21), abs. u. rel. Nierengewicht ↓ (♂: PND 21), abs. u. rel. Milzgewicht ↓ (♂/♀: PND 21), abs. u. rel. Thymusgewicht ↓ (♂: PND 21), F1 Adulte: Futteraufnahme ↓ (♀: 2. u. 3. Postnatal-Wo), KG ↓ (♂/♀: 2. u. 3. Behandlungs-Wo), F2-Jungtiere: abs. u. rel. Lebergewicht ↓ (♀: PND 21), abs. u. rel. Thymusgewicht ↓ (♂/♀: PND 21), abs. u. rel. Milzgewicht ↓ (♂/♀: PND 21)	Hirata-Koizumi et al. 2011 a
**Ratte**, SD, 7 ♂/Zeitpunkt	**Dominant-Letal-Test,** **30, 60, 90 d**, Verpaarung mit 1 unbehandeltem virginen ♀ (1 Wo) u. wöchentlicher Wechsel mit neuem unbehandeltem virginen ♀ (7 Wo lang) ohne weitere Al-Exposition, AlCl_3_ × 6 H_2_O, 0; 44,8; 447,6; 4476 mg/l Trinkwasser (0; 0,45; 45; 55 mg Al/kg KG u. d^b)^), 7 d/Wo	**4,5 mg Al/kg KG: NOAEL für Effekte auf die Fertilität u. Effekte auf ♂/♀ Reproduktionsorgane**, kein Effekt auf Anzahl der Implantationen u. Wurfgröße, Hoden: Histologie ohne auffällige Befunde	Dixon et al. 1979
**Maus**, Swiss, 10 ♂	**12 Wochen vor Verpaarung**, Verpaarung eines ♂ mit unbehandelten virginen ♀ (10 d) u. Untersuchung am GD20; **AlCl_3_**, 0, 1000, 1200, 1400 mg/l Trinkwasser (0, 100, 124, 135 mg AlCl_3_/kg KG u. d bzw. 0, 20, 25, 27 mg Al/kg KG u. d), 7 d/Wo	**20 mg Al/kg KG**: Anzahl Resorptionen/Gesamtzahl an Implantationsstellen ↓, Prozentsatz ♀ mit Resorptionen ↑, Hoden: geringe Proliferation des Bindegewebes, Nebenhoden: Spermienzahl ↓; **25 mg Al/kg KG**: Hoden: stark verstopfte Blutgefäße u. Zunahme des interstitiellen Bindegewebes, komplette Zerstörung von Samenkanälchen u. Ersatz durch nekrotische u. degenerierte Zellen, basale Zellschicht intakt; **27 mg Al/kg KG**: Prozentsatz trächtiger ♀ ↓, keine Effekte auf: Anzahl Implantationsstellen, Anzahl lebender Feten, Hoden: verstopfte Blutgefäße, Zunahme des interstitiellen Bindegewebes, Anzahl Leydig-Zellen ↑, Veränderungen von Samenkanälchen, große nekrotische Bereiche, Samenkanälchen in der Peripherie mit weniger oder keinen Spermatiden, Nebenhoden: keine Spermatozoen, Verstärkung der histologischen Veränderungen im Hoden mit der Zeit (Untersuchung nach 3, 6, 9, 12 Wo)	Mayyas et al. 2005
**Maus,** Swiss, 10 ♀	**12 Wo vor Verpaarung,** Verpaarung von zwei ♀ mit einem unbehandelten ♂ mit bekannter Fertilität (10 d) u. Untersuchung am GD20, **AlCl_3_,** 0, 1000, 1200, 1400 mg/l Trinkwasser (0, 75, 94, 117 mg AlCl_3_/kg KG u. d bzw. 0, 15, 19, 25 mg Al/kg KG u. d), 7 d/Wo	**ab 15 mg Al/kg KG**: KG, Wasseraufnahme ↓, Prozentsatz trächtiger ♀ ↓, Anzahl der Resorptionen/Gesamtzahl der Implantationen ↓, Tierzahl mit Resorptionen ↑; **23 mg Al/kg KG**: Ovarien: verstopfte Blutgefäße mit großer Anzahl atretischer Follikel, Anzahl der Implantationsstellen nicht verändert	Mohammad et al. 2008

^a)^ wenn nicht anders angegeben, sind die aufgeführten Veränderungen statistisch signifikant

^b)^ Umrechnungsfaktor 0,09 (subchronisch) nach EFSA Scientific Committee (2012)

abs.: absolutes; Al: Aluminium; AlCl_3_: Aluminiumchlorid; AlNH_4_(SO_4_)_2_: Aluminiumammoniumdisulfat; Al_2_(SO_4_)_3_: Aluminiumsulfat; d: Tag(e); GD: Gestationstag; k. A.: keine Angabe; KG: Körpergewicht; k. w. A.: keine weitere Angabe; MCHC: mittlere korpuskuläre Hämoglobin-Konzentration; PND: Postnataltag; rel.: relatives; TG: Test Guideline (Prüfrichtlinie); Wo: Woche(n)

Die vermeintliche Zwei-Generationenstudie von Trif et al. (2010) ist eine Ein-Generationenstudie. In dieser Studie sowie der Drei-Generationenstudie von Muselin et al. (2016) fehlen wesentliche Fertilitäts-, Gestations- und Überlebens-Indices. In der Studie von Kumar und Singh (2015) ist die Tierzahl mit fünf männlichen Mäusen sehr gering. Diese Studien werden aufgrund der genannten Defizite nicht zur Bewertung herangezogen.

In einer Studie nach OECD-Prüfrichtlinie 413 an Wistar-Ratten mit 13-wöchiger inhalativer Exposition gegen Aluminiumchlorid kam es bis zur höchsten Konzentration von 0,4 mg Al/m^3^ nicht zu Effekten auf die männlichen und weiblichen Reproduktionsorgane (BASF SE 2019 a). Eine weitere Studie mit inhalativer sechsmonatiger Exposition von F344-Ratten und Hartley-Meerschweinchen gegen Aluminiumchlorhydrat (Staub) ergab ebenfalls bis zur höchsten Konzentration von 7,7 mg Al/m^3^ keine histologischen Befunde in Hoden, Prostata, Samenbläschen, Ovarien und Uterus (Steinhagen et al. 1978).

In Tabelle 14 sind die Studien mit Untersuchung von Reproduktionsorganen nach wiederholter oraler Exposition gegen lösliche Aluminiumverbindungen aufgeführt. Studien mit einmaliger i.p. Gabe, ohne histologische Untersuchung oder einer geringeren Tierzahl als fünf pro Gruppe (Nager) wurden nicht aufgenommen.

**Tab. 14:** Studien mit Untersuchung der Reproduktionsorgane nach wiederholter oraler Exposition gegen lösliche Aluminiumverbindungen

**Spezies, Stamm, Anzahl pro Gruppe**	**Exposition**	**Befunde^a)^**	**Literatur**
**Männliche Reproduktionsorgane**			
**Ratte**, Wistar, 10 ♂	**120 d**, **AlCl_3_** 0, 64, 128, 256 mg/kg KG u. d (0, 13, 26, 52 mg Al/kg KG u. d), Trinkwasser, 7 d/Wo	**ab 13 mg Al/kg KG**: KG ↓, Nebenhoden: Anteil abnormaler Spermien ↑, Hoden: ACP- u. LDH-x-Aktivität ↓; **ab 26 mg Al/kg KG**: abs. u. rel. Hoden- u. Nebenhodengewicht ↓, Nebenhoden: Spermienzahl ↓, Hoden: Al-Konzentration ↑, SDH- u. LDH-Aktivität ↓	Zhu et al. 2014
**Ratte**, Albino, 6 ♂	**30 oder 60 d**, **AlCl_3_** 0, 40, 80 mg/kg KG u. d (0, 8, 16 mg Al/kg KG u. d), Gavage, Vehikel: destilliertes Wasser, k. A. ob 5 oder 7 d/Wo	**ab 8 mg Al/kg KG**: rel. Hodengewicht ↓ (nach 60 d), Hoden: Hyperplasie (30 u. 60 d), Ablösung des Epithels der Samenkanälchen (30 u. 60 d), zunehmender Schweregrad der histologischen Veränderungen mit Dosis u. Zeitdauer, Nebenhoden: Spermienzahl ↓ (nach 30 u. 60 d), Prozentsatz lebender Spermien ↓ (nach 30 u. 60 d), Prozentsatz abnormaler Spermien ↑ (nach 60 d); **bei 16 mg Al/kg KG**: KG ↓ (nach 30 u. 60 d), rel. Prostatagewicht ↓ (nach 60 d), rel. Hodengewicht ↓ (nach 30 d), Hoden: koagulative Nekrose des Samenkanälchenepithels (60 d), Hyperämie von Blutgefäßen (30 u. 60 d), interstitielles Ödem (30 u. 60 d), Leydigzellproliferation (30 u. 60 d), Oligospermie (60 d)	Abdul-Rasoul et al. 2009
**Ratte**, Wistar, 5 ♂	**112 d**, **AlCl_3_**, 0,000067; 0,000335; 10; 40 mg Al/kg KG u. d, Gavage, Vehikel: destilliertes Wasser, 7 d/Wo	**ab 0,000067 mg Al/kg KG**: abs. Hodengewicht ↓ (–8 bis –14 %), morphometrische Parameter der Leydig-Zellen verändert, Serumtestosteron ↓, alle Effekte ohne Dosisabhängigkeit, Integrität der Spermienmembran dosisabhängig ↓; KG, Histologie der Hoden u. Spermienmorphologie bis zur höchsten Dosis nicht verändert; Effekte bei der niedrigsten Dosis nicht plausibel	Mouro et al. 2018
**Ratte**, Wistar, 20 ♂	**120 d**, **AlCl_3_**, 0, 400, 800, 1600 mg/l Trinkwasser (7, 15, 30 mg Al/kg KG u. d^b)^), 7 d/Wo	**ab 7 mg Al/kg KG**: KG ↓, Hoden: Stroma leicht ausgedehnt, Anzahl spermatogener Zellen ↓ (zunehmender Schweregrad mit steigender Dosis); **15 mg Al/kg KG**: Hoden: interstitielle Eosinophilie, spermatogene Zellen ins Lumen ↓, Spermienzahl ↓; **30 mg Al/kg KG**: Hoden: Stroma stark ausgedehnt, Lumen der Samenkanälchen eng, Spermienzahl ↓	Sun et al. 2018
**Ratte**, Wistar, 5 ♂	**6**,** 12 oder 18 Mo**, **AlCl_3_**, 0, 180, 720, 3600 mg/l Trinkwasser (0, 3, 13, 65,6 mg Al/kg KG u. d^b)^), 7 d/Wo	**ab 3 mg Al/kg KG**: Hoden: Störungen der Spermatogenese (12 Mo); **13 mg Al/kg KG**: Hoden: Störungen der Spermatogenese (6 Mo), leichte degenerative Veränderungen mit fokalen Bereichen nekrotischer spermatogener Zellen (6 Mo); **65,6 mg Al/kg KG**: KG ↓ (18 Mo); Hoden: intensive Nekrosen (12 Mo), keine Spermatozoen im Lumen (12 Mo), Keimepithel in Samenkanälchen abgebaut (12 Mo); dosis- u. zeitabhängige Verstärkung der histologischen Effekte; dosis- u zeitabhängige Zunahme der Al-Konzentrationen in Plasma u. Hoden	Hichem et al. 2013
**Ratte**, Wistar, 6 ♂	**60 d**, **AlCl_3_ × 6 H_2_O**, 0; 1,5; 8,3 mg Al/kg KG u. d, Trinkwasser, 7 d/Wo	**ab 1,5 mg Al/kg KG**: Hoden: Spermienzahl ↓, ROS ↑, Nebenhoden: Spermientransitzeit ↑ (Spermienzahl nicht verändert), Spermien (aus Vas deferens): Anteil abnormaler Spermien ↑; **8,3 mg Al/kg KG**: Hoden: Anzahl leerer Samenkanälchen ↑, aktivierte Makrophagen ↑, Al-Konzentration ↑ (3,35 µg/g, Kontrolle: 1,79 µg/g, andere Gruppen nicht gemessen, Spermien (aus Vas deferens): Anzahl u. Beweglichkeit ↓; keine Veränderung: KG, Futter- u. Trinkwasseraufnahme	Martinez et al. 2017
	**42 d**, **AlCl_3_ × 6 H_2_O**, 0, 100 mg Al/kg KG u. d, Gavage, Vehikel: Wasser, 7 d/Wo	**Gavage**: **100 mg Al/kg KG**: ventraler Prostatalappen: abs. u. rel. Gewicht ↓, Hoden: Anzahl leerer Samenkanälchen ↑ (Anzahl aktivierter Makrophagen nicht erhöht), Spermienzahl ↓ (Nebenhoden: Spermienzahl u. Spermientransitzeit nicht verändert), ROS ↑, Spermien (aus Vas deferens): Anteil abnormaler Spermien ↑ u. Beweglichkeit ↓; keine Veränderung: KG, Futter- u. Trinkwasseraufnahme	
**Weibliche Reproduktionsorgane**			
**Ratte,** Wistar, 20 ♀	**120 d**, **AlCl_3_**, 0, 64, 128, 256 mg/kg KG u. d (0, 13, 26, 52 mg Al/kg KG u. d), Trinkwasser, 7 d/Wo	**ab 13 mg Al/kg KG**: Ovarien: ACP-, ALP- u. SDH-Aktivität ↓, Mg^2+^-, Na^+^-K^+^- u. Ca^2+^-ATPase-Aktivität ↓; **bei 52 mg Al/kg KG**: Ovarien: auffällige ultrastrukturelle Befunde, Al-Konzentration in Ovarien nicht bestimmt	Fu et al. 2014

^a)^ wenn nicht anders angegeben, sind die aufgeführten Veränderungen statistisch signifikant

^b)^ Umrechnungsfaktor 0,09 (subchronisch) nach EFSA Scientific Committee (2012)

ACP: saure Phosphatase; Al: Aluminium; AlCl_3_: Aluminiumchlorid; Akt.: Aktivität; ALP: alkalische Phosphatase; d: Tag(e); KG: Körpergewicht; LDH: Lactatdehydrogenase; LDH-x: Lactatdehydrogenase-Isoenzym; Mo: Monat(e); ROS: reaktive Sauerstoffspezies; SDH: Succinatdehydrogenase; TG: Test Guideline (Prüfrichtlinie); Wo: Woche(n)

In mehreren nicht nach gültigen Prüfrichtlinien durchgeführten Studien an Ratten mit Gabe von Aluminiumchlorid im Trinkwasser oder per Gavage kam es zu Effekten auf Spermien (Abdul-Rasoul et al. 2009; Hichem et al. 2013; Martinez et al. 2017; Sun et al. 2018; Zhu et al. 2014), Organgewichte (Abdul-Rasoul et al. 2009; Zhu et al. 2014) oder Histologie der Hoden (Abdul-Rasoul et al. 2009; Hichem et al. 2013; Martinez et al. 2017; Sun et al. 2018). Die Effekte bei extrem niedrigen Dosierungen (Mouro et al. 2018) sind im Vergleich zu den anderen Studien nicht plausibel. Die niedrigste orale Dosis mit Spermieneffekten bei Ratten betrug 1,5 mg Al/kg KG und Tag (verabreicht als Aluminiumchlorid im Trinkwasser; Martinez et al. 2017). Es wurden in dieser Studie jedoch nur sechs Tiere pro Dosis untersucht. Im Widerspruch dazu traten bei Ratten in den Studien nach OECD-Prüfrichtlinie 416 bis zur höchsten Dosis von ca. 31 mg Al/kg KG und Tag (Aluminiumsulfat) bzw. 36 mg Al/kg KG und Tag (Aluminiumammoniumsulfat) keine Effekte auf die Spermien auf (Hirata-Koizumi et al. 2011 a, b).

In einer Studie an weiblichen Wistar-Ratten kam es nach 120-tägiger Exposition gegen Aluminiumchlorid mit dem Trinkwasser bei 52 mg Al/kg KG und Tag zu auffälligen ultrastrukturellen Befunden in den Ovarien (Fu et al. 2014).

##### 5.5.1.2 In vitro

Die Beweglichkeit von Spermien gesunder, nicht rauchender Probanden wurde in vitro nach zweistündiger Inkubation mit 125–5000 µM Aluminiumchlorid untersucht. Ab 1000 µM war die Beweglichkeit der Spermien statistisch signifikant erniedrigt sowie die MDA-Konzentration statistisch signifikant erhöht, die NOAEC betrug 500 µM (Jamalan et al. 2016).

Für eine In-vitro-Studie wurden von mindestens 30 männlichen Spendern (Alter: 27–45 Jahre) Spermienproben hoher Qualität hinsichtlich der Anzahl und Beweglichkeit eingesetzt. Nach 180-minütiger Behandlung der Spermatozoen mit 0,5 mM Aluminiumchlorid waren der Prozentsatz der Akrosomen sowie die Plasmamembranintegrität, die Viabilität, das mitochondriale Membranpotenzial und die gesamte antioxidative Kapazität der Spermatozoen im Vergleich zur Kontrollgruppe erniedrigt, während die MDA-Konzentration im Vergleich zur Kontrolle erhöht war. Die gleichzeitige Gabe von Silymarin, einem wirksamen Antioxidans, verhinderte die Effekte von Aluminiumchlorid auf diese Parameter (Aghashahi et al. 2020).

##### 5.5.1.3 Fazit

###### 5.5.1.3.1 Fertilität

Aus Studien nach OECD-Prüfrichtlinie 422 oder 416 an Ratten ergeben sich NOAEL für Fertilität von 92,8 mg Al/kg KG und Tag (basisches Aluminiumchlorid; NOTOX B.V. 2007 a), 31,2 (♂)/52,0 (♀) mg Al/kg KG und Tag (Aluminiumsulfat; Hirata-Koizumi et al. 2011 b) und 36,3 (♂)/59,0 (♀) mg Al/kg KG und Tag (Aluminiumammoniumsulfat; Hirata-Koizumi et al. 2011 a). Die NOAEL entsprechen den jeweils höchsten Dosierungen. Bei Mäusen gibt es in Studien, die nicht nach gültigen Prüfrichtlinien durchgeführt sind, ab 15 mg Al/kg KG und Tag Effekte auf die Fertilität, allerdings waren auch Körpergewicht und Trinkwasseraufnahme deutlich vermindert (Aluminiumchlorid; Mayyas et al. 2005; Mohammad et al. 2008).

###### 5.5.1.3.2 Effekte auf die Reproduktionsorgane

In den Inhalationsstudien (BASF SE 2019 a; Steinhagen et al. 1978; Tabelle 8) wurden bei der histopathologischen Untersuchung keine Effekte auf die männlichen und weiblichen Reproduktionsorgane von Ratten oder Meerschweinchen festgestellt.

Die Reproduktionsorgane männlicher Tiere sind nach oraler Aufnahme im Vergleich zu weiblichen Tieren empfindlicher für Aluminium-induzierte Effekte.

#### 5.5.2 Entwicklungstoxizität

##### 5.5.2.1 Pränatale Entwicklungstoxizität

###### 5.5.2.1.1 Ratte

Die Daten aus Greim (2007) lassen sich wie folgt zusammenfassen und bewerten:

In Studien zur pränatalen Entwicklungstoxizität führten Aluminiumnitrat und Aluminiumchlorid bei Ratten nach Schlundsondengabe zu verringerten Fetengewichten und verzögerten Ossifikationen bei gleichzeitiger Maternaltoxizität in Form von verringerter Körpergewichtszunahme. In einer Studie traten auch Veränderungen der Rippen (kurze Rippe) auf, die nicht dosisabhängig waren (Greim 2007). In dieser Studie erfolgte die Darstellung der Variationen und Fehlbildungen nur auf Feten-, nicht auf Wurfbasis. Laut der DevTox-Datenbank handelt es sich bei einer kurzen Rippe um eine Veränderung, die zwischen Variation und Fehlbildung liegt (BfR 2021). Ab 13 mg Al/kg KG und Tag (Aluminiumnitrat) wiesen zudem die Muttertiere eine erniedrigte Körpergewichtszunahme und die Feten ein erniedrigtes Körpergewicht pro Wurf auf. Die Effekte sind als beginnende entwicklungstoxische Effekte mit Maternaltoxizität (ca. 20 % geringere Körpergewichtszunahme) zu sehen. Die Zahl verkümmerter Feten stieg dosisabhängig an, das Körpergewicht der Feten fiel dosisabhängig ab. Es ist unklar, ob dies ein direkter Effekt des Aluminiums ist oder sekundär entstanden ist. Das Fehlen des Schwertfortsatzes bei den Feten nach Schlundsondengabe von 133 mg Al/kg KG und Tag (Aluminiumcitrat) bei Sprague-Dawley-Ratten wird als Variation angesehen. Es waren 31/70 (44 %) Feten bzw. 9/15 (60 %) Würfen betroffen verglichen mit 20/90 (22 %) Feten bzw. 9/17 (53 %) Würfen bei den Kontrolltieren (Greim 2007).

Die einzige neu hinzugekommenen pränatale Entwicklungstoxizitätsstudie an Ratten kann aufgrund erheblicher methodischer Schwächen nicht zur Bewertung herangezogen werden: Zehn Ratten (k. A. zum Stamm) erhielten vom 0. bis 18. Gestationstag 193 mg Aluminiumsulfat/kg KG und Tag (0, 30 mg Al/kg KG und Tag). Eine Untersuchung der fetalen Wirbelsäule fand mit Hämatoxylin- und Eosin-Färbung am 18. Gestationstag statt. Die Muttertiere wiesen eine verminderte Körpergewichtszunahme sowie eine reduzierte Anzahl an Corpora lutea, Implantationsstellen und lebenden Feten pro Muttertier auf. Das Körpergewicht der Feten war vermindert (nicht auf Wurf bezogen), die Scheitel-Rumpf-Länge reduziert und der Prozentsatz äußerlich veränderter Feten (k. w. A.) erhöht (Yassa et al. 2017). Die Befunde waren Ossifikationsverzögerungen, keine Fehlbildungen. Es wurde nur eine Dosis eingesetzt, es fehlen Angaben zur Futter- und Trinkwasseraufnahme, eine Kontrollgruppe für die Sulfataufnahme und eine Untersuchung der Wirbelsäule mit Anfärbung von knöchernen und knorpeligen Strukturen mittels Alizarinrot und Alcianblau.

Ein NOAEL für pränatale entwicklungstoxische Effekte bei Ratten kann aus den vorliegenden Studien nicht abgeleitet werden. Die niedrigste Effektdosis lag bei 13 mg Al/kg KG und Tag (Aluminiumnitrat; Greim 2007).

###### 5.5.2.1.2 Maus

Die Daten aus Greim (2007) lassen sich wie folgt zusammenfassen und bewerten:

In Studien zur pränatalen Entwicklungstoxizität bei Mäusen hatten Aluminiumlactat und Aluminiumchlorid nach Schlundsondengabe eine erhöhte Anzahl an Resorptionen, verringerte Fetengewichte, verzögerte Ossifikationen, dorsale Hyperkyphose und/oder Gaumenspalten zur Folge, bei gleichzeitiger Maternaltoxizität wie verringerter Körpergewichtszunahme (Colomina et al. 1992; Cranmer et al. 1986). Die niedrigste Effektdosis lag bei 40 mg Al/kg KG und Tag (Cranmer et al. 1986). Die Resorptionen sind als schwerwiegend einzuordnen. Es liegen jedoch keine Angaben zum Körpergewicht der Muttertiere aus der Studie von Cranmer et al. (1986) vor, was die Bewertung erschwert. Bei Swiss-Mäusen trat nach Verabreichung von 57,5 mg Al/kg KG und Tag (Aluminiumlactat) per Schlundsonde bei den Feten eine erhöhte Inzidenz an Gaumenspalten auf (7/53 Feten (13 %), 4/10 Würfen (40 %), Kontrolle: 0/54 Feten, 0/13 Würfen) (Colomina et al. 1992). Es wurde nur eine Dosis verabreicht. Bei Mäusen kommt es mit einer Gestationsdauer von 19 bis 20 Tagen aufgrund des Gehirnwachstums zu einem schnellen Schädelwachstum. Das Wachstum in lateraler Richtung führt zu einer Vergrößerung des Spaltes, der durch die Gaumenplatten geschlossen werden muss. Falls die Rotation der Platten verspätet oder beeinträchtigt ist (z. B. durch Wachstumsverzögerungen beim Embryo) können sich die Platten nicht treffen und daher nicht fusionieren, was zu einer Gaumenspalte führt. Spezies mit einer längeren Gestationsdauer verfügen über ein proportional längeres Fenster für den Schluss der Gaumen, sodass Entwicklungsverzögerungen keinen derartigen Effekt auf den Schluss der Gaumen zur Folge haben (DeSesso und Scialli 2018).

Ein oraler NOAEL für pränatale entwicklungstoxische Effekte bei Mäusen kann aus den vorliegenden Studien nicht abgeleitet werden. Die niedrigste Effektdosis lag bei 40 mg Al/kg KG und Tag (Aluminiumchlorid). Die beobachteten Resorptionen sind als schwerwiegend einzuordnen. Es liegt keine Angabe zum Körpergewicht der Muttertiere vor.

##### 5.5.2.2 Perinatale Entwicklungstoxizität

In der bereits beschriebenen, kombinierten Studie nach OECD-Prüfrichtlinie 422 an männlichen und weiblichen Wistar Crl: (WI) BR-Ratten traten bis zu höchsten Dosis von 92,8 mg Al/kg KG und Tag bis zum 3. Postnataltag (Studienende) keine Effekte auf die Nachkommen auf (siehe Abschnitt 5.5.1.1; NOTOX B.V. 2007 a). In den ebenfalls bereits in Abschnitt 5.5.1.1 beschriebenen 2-Generationen-Studien nach OECD-Prüfrichtlinie 416 an Crl:CD(SD)-Ratten traten bis zum 4. Postnataltag bis zu den jeweils höchsten Konzentrationen (F0 ♂: 31,2; ♀: 52,0 mg Al/kg KG und Tag bzw. F0 ♂: 36,3; ♀: 59,0 mg Al/kg KG und Tag) keine Effekte auf die Nachkommen auf (Hirata-Koizumi et al. 2011 a, b).

Die höchste orale Dosis von 92,8 mg Al/kg KG und Tag entspricht damit dem NOAEL für perinatale Toxizität.

##### 5.5.2.3 Postnatale Entwicklungstoxizität und Entwicklungsneurotoxizität

Die Daten aus Greim (2007) lassen sich wie folgt zusammenfassen:

In prä- und postnatalen Entwicklungstoxizitätsstudien wurden mit löslichen Aluminiumsalzen Effekte auf das Verhalten beobachtet, meist bei gleichzeitig verringerter Körpergewichtszunahme der Nachkommen. Der NOAEL für entwicklungsneurotoxische Effekte von Aluminiumlactat nach oraler Applikation bei Swiss-Mäusen lag bei 10 mg Al/kg KG und Tag. Ab 50 mg Al/kg KG und Tag kam es zu einer verminderten Lernleistung im Morris-Water-Maze-Test sowie zu motorischen Effekten. Die Griffstärke der Hintergliedmaßen war bei 100 mg Al/kg KG und Tag verringert. Bei Neuseeländer-Kaninchen wurde nach s.c. Injektion von Aluminiumlactat ein NOAEL für entwicklungsneurotoxische Wirkungen von 2,7 mg Al/kg KG und Tag erhalten, wobei der NOAEL für maternale Toxizität bei 0,7 mg Al/kg KG und Tag lag. Bei 11 mg Al/kg KG und Tag kam es bei den Nachkommen zu erhöhter Mortalität, sowie zu einer verminderten klassischen Konditionierung. Es wurde ausführlich auf die methodischen Schwächen der Verhaltensuntersuchungen hingewiesen (Greim 2007).

Effekte mit der höchsten Konsistenz sind die verminderte Griffstärke der Vorder- und Hintergliedmaßen, reduzierte spontane motorische Aktivität, thermale Sensitivität und Schreckreaktion (ATSDR 2008).

Tabelle 15 umfasst die Studien zur postnatalen Entwicklungstoxizität und Entwicklungsneurotoxizität, die seit 2007 durchgeführt worden sind. Studien ohne pränatale Exposition werden nicht berücksichtigt.

**Tab. 15:** Entwicklungs(neuro)toxizitätsstudien mit prä-, peri- und postnataler Verabreichung löslicher Aluminiumverbindungen

**Spezies, Stamm, Anzahl pro Gruppe**	**Exposition**	**Befunde^a)^**	**Literatur**
**Ratte**, Sprague Dawley, je 20 ♀	**GD 6–PND 364**, **Aluminiumcitrat**, pH-Wert der Al-Lösung zwischen 6 u. 7 eingestellt, Zieldosis: 0, 30, 100, 300 mg Al/kg KG u. d, Trinkwasser, gemessene Dosis anhand Trinkwasserverbrauch: Muttertiere während Gestation: 0; 25,9–27,0; 94,3–102,0; 195,4–199,9 mg/kg KG u. d; Muttertiere Laktation: 0; 28,6–44,5; 99,9–165,2; 298,6–523,3 mg/kg KG u. d; Nachkommen: Zieldosen bis 7. Lebenswoche überschritten (bis ca. 200 %), danach unterschritten (auf ca. 30–50 %), Kontrolle: unbehandeltes Trinkwasser oder Natriumcitrat (27,2 g/l Trinkwasser), nach Geburt: Würfe verkleinert auf je 4 ♂ u. ♀, Doppelblindstudie **OECD TG 426**	**27 mg Al/kg KG: NOAEL neuromuskuläre Effekte auf die Nachkommen**; **102 mg Al/kg KG: NOAEL Maternaltoxizität**; **ab 102 mg Al/kg KG**: ♂/♀ Nachkommen: Griffstärke Vorder-u. Hintergliedmaßen dosisabhängig ↓ (Effekte stärker bei jüngeren Tieren, KG nicht verändert), ♂ Nachkommen: Harnwegssystem: Hydronephrose, Dilatation der Urethra, Calculi, ♀ Nachkommen: Beeinträchtigung der Fußspreizung (Effekte geringer ausgeprägt als bei Griffstärke); **bei 199,9 mg Al/kg KG**: Muttertiere: Diarrhoe, ♂/♀ Nachkommen: KG-Zunahme ↓, verzögerte sexuelle Reife und verzögerter Eintritt der Entwicklungsmeilensteine, Aufblähung des Abdomens, Hämaturie, Diarrhoe, ♂ Nachkommen: Mortalität ↑, ♀ Nachkommen: Harnwegssystem: Hydronephrose, Dilatation der Urethra, Calculi, leichte mikrozytäre Anämie; Nachkommen: Neuropathologie, motorische Aktivität u. auditorische Schreckantwort, T-Maze-Test, Morris-Water-Maze-Test (kein Effekt auf Lernen u. Gedächtnis) ohne Befund, keine Al-Messung im Urin	Poirier et al. 2011; ToxTest Alberta Research Council Inc. 2010
**Ratte**, Wistar, 5 ♂, 10 ♀	**Beginn der Verpaarung bis zum Alter von 4 Mo**, **AlCl_3_**, 0, 3000 mg/l Trinkwasser (0, ca. 270 mg/kg KG u. d^b)^, 0, 55 mg Al/kg KG u. d), Kontrollgruppe: Trinkwasser, Untersuchungen: immunhistochemische Lokalisierung der Tyrosinhydroxylase in Substantia nigra (Alter 4 Mo), lokomotorische Aktivität: Open-Field-Test (Alter 4 Mo)	**bei ca. 55 mg Al/kg KG**: Nachkommen: lokomotorische Aktivität ↓, Tyrosinhydroxylase ↓	Erazi et al. 2011
**Ratte**, Wistar, Dosisgruppe: 13 ♀, Kontrollgruppe 23 ♀	**GD 6–PND 21 od. PND 70**, **AlCl_3_**, 0, 500 mg/l Trinkwasser (0, ca. 45 mg AlCl_3_/kg KG u. d^b)^, 0, ca. 9 mg Al/kg KG u. d), Kontrollgruppe: unbehandeltes Trinkwasser, Untersuchungen: ♂ Nachkommen: Open-Field-Test (PND 70)	**bei ca. 9 mg Al/kg KG**: Nachkommen: KG-Zunahme ↓ (in 1. u. 2. Wo), Open-Field-Test: Latenzperiode ↓, Anzahl der betretenen Quadrate ↑ (Effekte stärker ausgeprägt, wenn Al-Behandlung durchgehend bis PND 70); keine Effekte auf: Gestationsdauer, Geschlechterverhältnis, Open-Field-Test: Anzahl des Schnüffelns u. Streckens	Kinawy und Al-Eidan 2018
**Ratte**, Wistar, je 6 ♀	**GD 14–PND 14**, **AlCl_3_**, 0, 400 mg/l Trinkwasser (0; 15,1 mg AlCl_3_/Tier u. d, mittleres KG: 207,7 g, ergibt 15 mg Al/kg KG u. Tag), Kontrollgruppe: unbehandeltes Trinkwasser, Untersuchungen: Histologie Cerebellum (PND 14)	**bei 15 mg Al/kg KG**: Muttertiere: KG ↓, Futter- u. Trinkwasseraufnahme ↓, abs. u. rel. Cerebellum-Gewicht ↓, Al-Konzentration in Plasma u. Cerebellum ↑, Nachkommen: KG ↓, abs. u. rel. Cerebellum-Gewicht ↓, Al-Konzentration in Plasma und Cerebellum ↑, Cerebellum: Purkinje-Zellkörper kaum differenziert, häufig mit pyknotischen Zellen u. ausgeprägtem Ödem	Ghorbel et al. 2016
**Maus**, Slc: ICR, je 10‑12 ♀	**GD 6–PND 21**, **AlCl_3_ × 6 H_2_O**, 0, 900, 1800 mg/l Trinkwasser (0, 182, 364 mg Al/l Trinkwasser, Muttertiere während Gestation: 0, 108, 207 mg Al/kg KG u. Tag, Muttertiere während der Laktation: 0; 320; 575 mg Al/kg KG u. Tag), Untersuchungen: ♂ Nachkommen: Histologie u. Immunhistochemie (PND 21, PND 77)	**108 mg Al/kg KG**: Nachkommen: KG ↓ (♀: PND 21); **207 mg Al/kg KG**: Muttertiere: Trinkwasseraufnahme ↓ (GD 17‑PND 21), Nachkommen: KG ↓ (♂: PND 21, ♀, ♂: PND 77), ♂: Hilus des Gyrus dentatus im Hippocampus: Anzahl p21^Cip1/Waf1+^-positiver Zellen ↓ (PND 77, Hinweis auf beeinträchtigte Neurogenese im Hippocampus); keine Effekte: Muttertiere: KG während Gestation u. Laktation, Futteraufnahme während Gestation, Anzahl Implantationsstellen, Anzahl lebender Nachkommen, abs. u. rel. Gehirngewicht (PND 21), Nachkommen: Geschlechterverhältnis, Histopathologie Gehirn (♂: PND 21, PND 77), k. A. zum pH-Wert der Al-Lösung, keine passende Kontrolle für Chloridaufnahme	Inohana et al. 2018

^a)^ wenn nicht anders angegeben, sind die aufgeführten Veränderungen statistisch signifikant

^b)^ Umrechnungsfaktor 0,09 (subchronisch) nach EFSA Scientific Committee (2012)

abs.: absolut; Al: Aluminium; AlCl_3_: Aluminiumchlorid; d: Tag(e); FOB: functional observational battery; GD: Gestationstag; k. (w.) A.: keine (weitere) Angabe; Mo: Monat(e); PND: Postnataltag; rel.: relativ; TG: Test Guideline (Prüfrichtlinie); Wo: Woche(n)

In einer Studie mit prä- und postnataler Exposition von Sprague-Dawley-Ratten nach der OECD-Prüfrichtlinie 426 lagen die Zieldosierungen bei 0, 30, 100 und 300 mg Al/kg KG und Tag, basierend auf einer erwarteten Trinkwasseraufnahme von 120 ml/kg KG und Tag. Anhand des gemessenen Trinkwasserverbrauchs war die Aluminiumaufnahme jedoch während der Gestation geringer, in der frühen Postnatalzeit etwa doppelt so hoch während der ersten sieben Wochen, später geringer. Die Muttertiere der höchsten Dosisgruppe hatten während der Gestation Diarrhoe. Die männlichen und weiblichen Nachkommen wiesen ab der mittleren Konzentration eine dosisabhängig erniedrigte Griffstärke der Vorder- und Hintergliedmaßen auf, was bei jüngeren Tieren stärker ausgeprägt war. Damit einhergehend war auch ein Effekt auf die Fußspreizung festzustellen, der jedoch im Vergleich zum Effekt auf die Griffstärke geringer ausgeprägt war. Die Neuropathologie und Verhaltenstests waren ohne auffällige Befunde. Männliche Nachkommen reagierten für Effekte auf das Harnwegssystem, die ab der mittleren Dosis auftraten, empfindlicher als weibliche Nachkommen. Der NOAEL für neuromuskuläre Effekte auf die Nachkommen lag bei 27 mg Al/kg KG und Tag (Dosis der Muttertiere während Gestation). Gewebemessungen von Aluminium bei den Nachkommen ergaben, dass diese dosisabhängig waren, wobei die Aluminiumkonzentration im Gehirn die beste Assoziation mit der gegebenen Dosis aufwies. Bei den Geweben des ZNS war die höchste Aluminiumkonzentration im Hirnstamm festzustellen (Poirier et al. 2011; ToxTest Alberta Research Council Inc. 2010). Einer Evaluierung des Joint FAO/WHO Expert Committee on Food Additives zufolge wird der NOAEL leicht überschätzt, wenn die Effekte auf die Nachkommen in utero vermittelt werden. Der NOAEL wird dann unterschätzt, wenn die Effekte über die Laktation oder die ersten Wochen nach dem Absetzen vermittelt werden. Da der Effekt auf die Griffstärke bei jüngeren Tieren stärker ausgeprägt war, kommt der In-utero-Exposition und der Exposition während der Laktation eine höhere Bedeutung zu als der späteren Expositionszeit mit der geringeren Trinkwasseraufnahme (WHO 2012). Der NOAEL für Maternaltoxizität liegt bei 102 mg Al/kg KG und Tag, da die Muttertiere während der Gestation in der hohen Dosisgruppe Diarrhoe hatten.

Es liegen drei Studien an Wistar-Ratten mit prä- und postnataler Gabe von jeweils nur einer Dosis AlCl_3_ mit dem Trinkwasser vor. Bei allen Studien traten bei 400, 500 bzw. 3000 mg/l Trinkwasser (ca. 9, 15 bzw. 55 mg Al/kg KG und Tag) entwicklungsneurotoxische Effekte wie eine erniedrigte lokomotorische Aktivität bzw. Effekte im Open-Field-Test oder histopathologische Veränderungen im Gehirn auf (Erazi et al. 2011; Ghorbel et al. 2016; Kinawy und Al-Eidan 2018). Da jeweils nur eine Dosis eingesetzt wurde, lässt sich keine Aussage zur Dosis-Wirkungs-Beziehung treffen. Weitere Kritikpunkte sind eine geringe Tierzahl, fehlende Angaben zu Maternaltoxizität, Körpergewicht, Futter- und Trinkwasseraufnahme, Verblindung der Verhaltenstoxizitätsuntersuchung und fehlende Kontrolle für die Chloridaufnahme. Aufgrund der beschriebenen Unsicherheiten sind die Studien nicht zur Bewertung der Entwicklungsneurotoxizität geeignet.

Bei einer Untersuchung an Slc:ICR-Mäusen mit der Gabe von Aluminiumchlorid vom 6. Gestationstag bis zum 21. Postnataltag mit dem Trinkwasser zeigten sich bei der höheren von zwei Dosierungen von 207 mg Al/kg KG und Tag bei den männlichen Nachkommen Hinweise auf eine beeinträchtigte Neurogenese im Hippocampus bei reduziertem Körpergewicht. Maternaltoxizität trat nicht auf (Inohana et al. 2018). Es fehlen Angaben zum pH-Wert des Trinkwassers und eine passende Kontrolle für die Chloridaufnahme.

Einen verlässlichen Vergleich der Qualität und Stärke von Aluminium-induzierten neurotoxischen Effekten bei juvenilen und adulten Tieren vorzunehmen, ist aufgrund des Einflusses von Alter und Körpergewicht sowie der oben beschriebenen Schwierigkeiten nicht möglich. Zudem liegt hierfür nur eine Studie vor, bei der die Muttertiere bei prä- und postnataler Gabe bei Tests der Griffstärke von Vorder- und Hintergliedmaßen sowie negative Geotaxis ohne Effekte blieben, während bei den Nachkommen derartige Effekte festzustellen waren (Greim 2007).

##### 5.5.2.4 Fazit

In Studien zur pränatalen Entwicklungstoxizität nach Schlundsondengabe führen Aluminiumnitrat und Aluminiumchlorid bei Ratten sowie Aluminiumlactat und Aluminiumchlorid bei Mäusen zu entwicklungstoxischen Effekten bei gleichzeitiger Maternaltoxizität in Form von verringerter Körpergewichtszunahme. Im Vordergrund stehen verringerte Fetengewichte und verzögerte Ossifikationen. Die niedrigste Effektdosis liegt für Ratten bei 13 mg Al/kg KG und Tag (Aluminiumnitrat) und bei Mäusen bei 40 mg Al/kg KG und Tag (Aluminiumchlorid). Ein NOAEL für pränatale Entwicklungstoxizität bei Ratten und Mäusen kann nicht abgeleitet werden (Greim 2007).

Mehrere Studien mit methodischen Schwächen zeigen entwicklungsneurotoxische Effekte wie verminderte Griffstärke der Vorder- und Hintergliedmaßen, reduzierte motorische Aktivität und reduzierte Schreckreaktion bei Ratten, Mäusen und Kaninchen, zum Teil ohne Maternaltoxizität. In einer Studie an Sprague-Dawley-Ratten nach OECD-Prüfrichtlinie 426 mit der Gabe von Aluminiumcitrat über das Trinkwasser kam es bei den Nachkommen ab 102 mg Al/kg KG und Tag zu neuromuskulären Effekten, einer dosisabhängig erniedrigten Griffstärke bei Vorder- und Hintergliedmaßen sowie zu einer Beeinträchtigung der Fußspreizung. Der NOAEL lag bei 27 mg Al/kg KG und Tag (Poirier et al. 2011; ToxTest Alberta Research Council Inc. 2010).

### 5.6 Genotoxizität

#### 5.6.1 In vitro

##### 5.6.1.1 Bakterien

Übereinstimmend ist in Übersichtsarbeiten für lösliche Aluminiumverbindungen in einer Vielzahl von Untersuchungen an Bakterien keine mutagene Wirkung beschrieben (ATSDR 2008; COT 2013; EFSA 2008; Greim 2007; NEG und DECOS 2011). Von einem positiven Ergebnis in Konzentrationen von 20 bis 5000 μg Aluminiumchlorid/Platte wird für die Salmonella-typhimurium-Stämme TA98 und TA100 im Präinkubationstest mit und ohne Zusatz metabolischer Aktivierung berichtet, die Stämme TA1535, TA1537 und TA1538 hingegen lieferten unter den gleichen Bedingungen negative Ergebnisse (NEG und DECOS 2011). Die Studie ist nicht im Original erhältlich. Acht weitere Genmutationstests nach OECD-Prüfrichtlinie 471 (von 1997), die auch die Salmonella-typhimurium-Stämme TA98 und TA100 in Konzentrationen bis 5000 μg/Platte untersuchten, sind negativ (ECHA 2020 a, b, c, 2022 b, 2023 c; Harlan Cytotest Cell Research GmbH 2015; Hoechst Marion Roussel Deutschland GmbH 1999 a; NOTOX B.V. 2010 c).

##### 5.6.1.2 Säugetierzellen

Zahlreiche Mutationstests in Säugetierzellen zeigen ebenfalls keine mutagene Wirkung (ATSDR 2008; COT 2013; EFSA 2008; Greim 2007; NEG und DECOS 2011). Dies wird bestätigt durch einen neuen HPRT-Test mit V79-Zellen nach OECD-Prüfrichtlinie 476 mit Aluminiumdiacetat (ECHA 2020 c) und sechs neue nach OECD-Prüfrichtlinie 476 durchgeführte TK^+/–^-Mutationstests mit L5178Y-Mauslymphomzellen mit Aluminiumchlorid, -chlorhydrat, -chloridhydroxysulfat, -trilactat, -ammoniumdisulfat und Aluminiumsulfat, basisch (Covance Laboratories Ltd 2010; ECHA 2020 a, b, 2022 b, 2023 c; NOTOX B.V. 2010 b). Für Aluminiumchlorid, Aluminiumnitrat und einen Aluminiummaltolat-Komplex wurden Effekte auf die DNA wie Komplexierung, Konformationsänderungen, Entwindung und Präzipitation von Chromatin beschrieben (Greim 2007). Untersuchungen mit Aluminiumchlorid zeigten die Bildung von DNA-Protein-Cross-Links (Greim 2007; Wedrychowski et al. 1986).

Zu den Endpunkten DNA-Schäden, Schwesterchromatidaustausch (SCE), Chromosomenaberrationen und Mikronukleusbildung sind die Untersuchungen aus Greim (2007) sowie neue publizierte Daten im Folgenden und in Tabelle 16 zusammengefasst:

In der humanen Brustepithelzelllinie MCF-10A wurde mit Aluminiumchlorid ab 0,3 µg Al/ml und ab einer Stunde Inkubation statistisch signifikant vermehrt phosphoryliertes Histon H2AX (γ-H2AX) nachgewiesen. Dies gilt auch für primäre humane Brustzellen nach einer 16-stündigen Inkubation mit 2,7 µg Al/ml. Mit Keratinozyten (HaCaT) verlief der Test negativ. In einem zusätzlichen Experiment wurden MCF-10A-Zellen mit 8,1 µg Al/ml (Aluminiumchlorid) für 16 Stunden inkubiert und bestrahlt (1 Gy). Nach acht und 24 Stunden erfolgte die Auszählung der γ-H2AX-Foci. Die Inkubation mit Aluminiumchlorid ergab keinen Unterschied zur Kontrolle. Die Autoren leiten daraus ab, dass die DNA-Reparatur nicht durch Aluminium beeinträchtigt wird (Sappino et al. 2012). Die Phosphorylierung von H2AX geschieht im Zuge der DNA-Schadenserkennung. Meist sind DNA-Doppelstrangbrüche der Auslöser, aber auch Einzelstrangbrüche, z. B. ausgelöst durch UV-Strahlung oder oxidativen Stress (Mishima 2017), sowie auch DNA-Schäden während der Proliferation oder der Apoptose. Die erhöhte γ-H2AX-Antikörper-Bindung ist daher nicht ausschließlich ein Nachweis induzierter Doppelstrangbrüche, sondern generell von DNA-Schäden. Da in der Veröffentlichung von Sappino et al. (2012) Foci in Zellen gezählt wurden, wurden die Daten nicht durch die ebenfalls beobachtete Apoptose verfälscht, da Apoptose zu einer gleichmäßigen Färbung des Zellkerns führt und nicht zu Foci (Solier und Pommier 2009). Dies wurde auch bestätigt durch das Anfärben der Zellen mit Annexin V, welches keinen Hinweis auf Apoptose ergab (Sappino et al. 2012). Um zu beweisen, dass es sich zweifelsfrei um DNA-Doppelstrangbrüche handelt, muss die Co-Lokalisation eines zweiten Proteins (z. B. 53BP1) nachgewiesen werden (Rothkamm et al. 2015). Dies ist im vorliegenden Fall nicht geschehen. So wurde in dieser Studie der Anstieg nicht weiter spezifizierter DNA-Schäden über die γ-H2AX-Quantifizierung nachgewiesen. Die angegebene Zeit nach Bestrahlung zur Untersuchung der DNA-Reparaturhemmung ist mit 8 bzw. 24 Stunden zu lange gewählt und es wird nicht gezeigt, dass in dem Versuchsaufbau auch tatsächlich durch die Bestrahlung Schäden entstanden sind.

Die dreistündige Inkubation von V79-Zellen mit Aluminiumchlorid in Konzentrationen bis zu 26,5 µg Al/ml in serumfreiem Medium induzierte keine DNA-Schäden bei einem Nachweis mittels einer FACS (fluoreszenzaktivierte Zellanalyse)-Untersuchung von γ-H2AX. Unter physiologischen Bedingungen (ca. 10 % FCS (fetales Kälberserum)) akkumulierte statistisch signifikant weniger Aluminium (etwa achtfach) intrazellulär in den V79-Zellen als unter serumfreien Bedingungen. Zudem zeigten sich ab 2,7 µg Al/ml eine statistisch signifikante Verminderung der Zellviabilität sowie die Zunahme von Nekrose (Tenan et al. 2021). Eine Übertragbarkeit der Ergebnisse auf die Situation in vivo ist somit stark limitiert.

Aluminiumchlorid induzierte in murinen (HC11) und humanen (NMuMG) Brustepithelzelllinien nach 24 Stunden Inkubation mit 2,7 µg Al/ml eine statistisch signifikante Erhöhung an DNA-Schäden in Form von γ-H2AX-Foci. Bei den sieben Tage lang mit 2,7 µg Al/ml behandelten HC11-Zellen trat eine leichte Verminderung der Proliferation, aber keine erkennbare Zytotoxizität (Annexin V/Propidiumiodid-Apoptose-Assay) auf (Mandriota et al. 2020). Auch in dieser Studie fehlt der Nachweis einer Co-Lokalisation eines zweiten Proteins, sodass hier nicht weiter spezifizierte DNA-Schäden über die γ-H2AX-Quantifizierung nachgewiesen wurden.

Weitere Untersuchungen zu (oxidativen) DNA-Schäden zeigten mit Aluminiumchlorid in humanen Lymphozyten oder Fibroblasten-Zelllinien ein positives Ergebnis entweder bei zytotoxischen Konzentrationen oder ohne entsprechende Messung dazu (Lankoff et al. 2006; Lima et al. 2007; Viau et al. 2021). In Abwesenheit von Zytotoxizität konnten in humanen Neuroblastom-, Brust-, Lymphozyten-, Colon- und Leberzelllinien keine DNA-Schäden nachgewiesen werden (Caicedo et al. 2008; Jalili et al. 2022; Roszak et al. 2017; Villarini et al. 2017). In drei Untersuchungen wurde eine Zunahme an SCE beobachtet, mit und ohne gleichzeitige Zytotoxizität bzw. in der dritten Studie wurde keine Zytotoxizität bestimmt (Türkez und Geyikoğlu 2011; Roy et al. 1990; Patel et al. 2009).

In einer Untersuchung zu chromosomalen Rearrangements wurde die murine Brustepithelzelllinie HC11 71 Wochen lang mit 10 oder 100 µM Aluminiumchlorid (0,27 bzw. 2,7 µg Al/ml) und die Zelllinie NMuMG 38 oder 40 Wochen lang mit 100 µM Aluminiumchlorid kultiviert. In den HC11-Zellen stieg die Gesamtzahl an chromosomalen Rearrangements konzentrationsabhängig an. Im Vergleich zur mitgeführten Kontrolle zeigte sich in den mit 10 oder 100 µM inkubierten HC11-Zellen ein 2,3-facher bzw. 3,0-facher Anstieg an einzigartigen chromosomalen Rearrangements. Die modale Chromosomenzahl konnte in der mit 2,7 µg Al/ml kultivierten Zellpopulation aufgrund zu starker Schwankungen nicht bestimmt werden, was auf starke genomische Instabilität hindeutet. Ein Verlust oder Zugewinn eines bestimmten Chromosoms wurde nicht beobachtet. Auch für die parentale Zelllinie ist eine Erhöhung der modalen Chromosomenanzahl beschrieben. Die Gesamtzahl sowie die der einzigartigen chromosomalen Rearrangements war auch bei der mit Wasser behandelten Kontrolle (nach 71 bzw. 38 Wochen Inkubation) gegenüber der parentalen Zelllinie (Zeitpunkt Null der Inkubation) erhöht. Auch in den NMuMG-Zellen nahm durch Aluminiumchlorid sowohl die Gesamtzahl als auch die Anzahl der für die jeweilige Behandlung einzigartigen chromosomalen Rearrangements zu. Etwa 70 % der mit Aluminium behandelten Zellen trugen eine Translokation mit Amplifikation von Chromosom 9 auf Chromosom 12. In einem weiteren Ansatz führte die 24-stündige Inkubation von HC11-Zellen und NMuMG-Zellen mit 2,7 µg Al/ml zu einer statistisch signifikanten Zunahme an Fragmentierungen, wobei nur bei den HC11-Zellen zusätzlich Chromosomenbrüche signifikant erhöht waren. In den HC11-Zellen tragen beide Allele für p53 eine Mutation. Ein Zytotoxizitätstest (Annexin V/Propidiumiodid-Apoptose-Assay) nach bis zu sieben Tagen war negativ (Mandriota et al. 2020). Die gesamten Ergebnisse der Langzeitversuche sind ohne erkennbare statistische Auswertung dargestellt. Angaben zur Zytotoxizität über einen längeren Inkubationszeitraum liegen nicht vor. Bei den Untersuchungen mit 100 µM ist eine Ausflockung von Proteinen anzunehmen. Eine Zunahme an chromosomalen Rearrangements mit der Zeit zeigt sich bereits bei den unbehandelten Zellen. Die Effekte nach der sehr langen Inkubationszeit von 38 bzw. 71 Wochen könnten zu einem Selektionsprozess der Zellen führen, wodurch nur jene Zellen überleben, die Aluminiumchlorid tolerieren. Insgesamt kann die Studie nicht überzeugend zeigen, dass Aluminiumchlorid die genomische Instabilität verursacht hat.

Chromosomenaberrationen wurden in humanen Lymphozyten durch 3,2 µg Al/ml, gegeben als Aluminiumsulfat, induziert. Ein verminderter mitotischer Index als Hinweis auf Zytotoxizität war nur bei den Lymphozyten männlicher Probanden in der höchsten der drei Altersgruppen (41–50 Jahre) festzustellen (Greim 2007; Roy et al. 1990). Bei humanen Lymphozyten kam es ebenfalls bei 3,2 µg Al/ml zu Chromosomenaberrationen, wobei keine Zytotoxizität untersucht wurde (Türkez und Geyikoğlu 2011). Da in dieser Studie nicht zwischen Gaps und anderen Aberrationstypen unterschieden und nur ein Zehntel der vorgeschriebenen Metaphasen pro Konzentration untersucht wurde, wird diese Studie nicht zur Bewertung herangezogen.

In einer weiteren Untersuchung mit humanen Lymphozyten waren ab 0,1 µg Al/ml, gegeben als Aluminiumchlorid, Polyploidien, Endoreduplikationen sowie die Summe von Gaps und Brüchen statistisch signifikant erhöht. Die Einzelauswertung der Gaps und Brüche zeigte einen Anstieg ohne statistische Signifikanz. Gleichzeitig trat Zytotoxizität auf (Lima et al. 2007). Ebenfalls an humanen Lymphozyten induzierte Aluminiumchlorid in einer hohen und zytotoxischen Konzentration von 607 µg Al/ml eine erhöhte Anzahl an Chromosomenaberrationen (Patel et al. 2009).

In der Lungenzelllinie V79 führte Aluminiumchlorid ab 0,3 µg Al/ml zur konzentrationsabhängigen Zunahme von Chromosomenbrüchen. Inkubiert wurde eine Stunde lang ohne Serum, dann 23 Stunden mit 1 % FCS. Gaps wurden nicht bestimmt. Ab 2,7 µg Al/ml zeigte sich eine statistisch signifikante Verminderung der Zellviabilität sowie eine Zunahme von Nekrose (Tenan et al. 2021). Wie oben bereits beschrieben, ist eine Übertragbarkeit der Ergebnisse auf die In-vivo-Situation stark limitiert.

Ein Chromosomenaberrationstest an CHL-Zellen war bis zu einer Konzentration von 104,5 µg Al/ml, gegeben als Aluminiumkaliumdisulfat, negativ (Ishidate et al. 1988), ebenso wie zwei neuere Chromosomenaberrationstests nach OECD-Prüfrichtlinie 473 mit Aluminiumdiacetat bzw. -ammoniumdisulfat an humanen Lymphozyten (ECHA 2020 a, c).

Aluminiumsulfat führte zu positiven Ergebnissen in Mikronukleustests mit humanen Lymphozyten bzw. dermalen Fibroblasten (Greim 2007; Migliore et al. 1999; Roy et al. 1990; Trippi et al. 2001; Viau et al. 2021). Das Ergebnis einer Zentromerfärbung mittels Fluoreszenz-in-situ-Hybridisierung (FISH) interpretieren die Autoren so, dass das Aluminiumion sowohl aneugen als auch klastogen wirken könnte, aber aufgrund des höheren prozentualen Anteils an Mikronuklei, die ein ganzes Chromosom enthalten, eine Störung der Chromosomenverteilung bewirkt (Migliore et al. 1999; Greim 2007). Auch Aluminiumchlorid führte in humanen Lymphozyten ab der niedrigsten Konzentration von 0,2 µg Al/ml zu einer erhöhten Anzahl an Mikronuklei. Es zeigte sich eine signifikante Zunahme an Zentromer-positiven und -negativen Mikronuklei (nur bei 1 µg Al/ml mit der FISH-Methode untersucht), was auf eine klastogene und aneugene Wirkung hindeutet. Ab 0,4 µg Al/ml wurde ein Anstieg der Apoptose gemessen, weshalb die Mikronuklei ab dieser Konzentration auch durch zytotoxische Effekte bedingt sein können (Banasik et al. 2005). Bei der sehr hohen Konzentration von 607 µg Al/ml, gegeben als Aluminiumchlorid, war das Ergebnis bei gleichzeitiger Aneuploidie positiv (Patel et al. 2009). Ab 0,1 µg Al/ml nahmen die Mikronuklei in Humanlymphozyten statistisch signifikant zu (k. A. zur Zytotoxizität) (Paz et al. 2017). In dieser Studie hatte bereits die Negativkontrolle eine hohe Anzahl an Mikronuklei.

Ein Test nach OECD-Prüfrichtlinie 487 mit Aluminiumlactat zeigte einen konzentrationsabhängigen Anstieg an Mikronuklei in humanen Lymphozyten (ECHA 2022 b). Hingegen war in drei weiteren Mikronukleustests ein negatives Ergebnis festzustellen (ECHA 2020 b, 2023 c; Jalili et al. 2020; NOTOX B.V. 2010 a). Zwei der Tests wurden nach OECD-Prüfrichtlinie 487 mit humanen Lymphozyten mit basischem Aluminiumchlorid (NOTOX B.V. 2010 a) bzw. Aluminiumsulfat, basisch (ECHA 2023 c) durchgeführt und ergaben mit und ohne Zusatz metabolischer Aktivierung keine erhöhte Mikronukleusbildung.

##### 5.6.1.3 Fazit

Lösliche Aluminiumverbindungen sind in zahlreichen bakteriellen Testsystemen, HPRT- und TK^+/–^-Mutationstests in Säugetierzellen nicht mutagen. DNA-schädigende Wirkungen sowie die Bildung von SCE, Chromosomenaberrationen und Mikronuklei lassen sich meist nur in Anwesenheit von Zytotoxizität oder bei einer hohen Aluminiumkonzentration beobachten. Vor allem zahlreiche neue nach aktuellen Prüfrichtlinien durchgeführte Chromosomenaberrations- und Mikronukleustests sind bis auf eine Ausnahme negativ.

**Tab. 16:** (oxidative) DNA-Schäden, Schwesterchromatidaustausche, Chromosomenaberrationen und Mikronukleus-Bildung durch lösliche Aluminiumverbindungen bei Säugetierzellen in vitro

**Endpunkt**	**Testsystem**	**Konzentration und Zeit**	**wirksame Konz.^a)^**	**Zytotox.^a)^/Anmerkung**	**Ergebnis**		**Literatur**
					**–m. A.**	**+m. A.**	
DNA-Schäden (γ-H2AX)	AlCl_3_ × 6 H_2_O, in H_2_O, MCF-10A-Brustepithelzelllinie	0, 10, 100, 300 µM (0,3; 2,7; 8,1 µg Al/ml), 1 h, 16 h	ab 0,3 µg Al/ml	keine (Apoptose-Messung)	+	n. u.	Sappino et al. 2012
	AlCl_3_ × 6 H_2_O, in H_2_O, primäre humane Brustzellen	0, 100, 300 µM (2,7; 8,1 µg Al/ml), 16 h	ab 2,7 µg Al/ml	n. u.	+	n. u.	Sappino et al. 2012
	AlCl_3_ × 6 H_2_O, in H_2_O, HaCaT-Keratinozyten	0, 100, 300 µM (2,7; 8,1 µg Al/ml), 16 h	–	n. u.	+	n. u.	Sappino et al. 2012
	AlCl_3_ × 6 H_2_O, in H_2_O, V79-Zellen (Lungenfibroblasten-Zellen Hamster)	0, 10, 100, 300, 1000 µM (0,3; 2,7; 8,1; 27 µg Al/ml), 3 h	–	Messung in G1-Phase; ab 2,7 µg Al/ml: Viabilität ↓, Nekrose ↑ (Annexin V/Propidiumiodid-Apoptose-Assay), serumfreie Inkubation	+	n. u.	Tenan et al. 2021
	AlCl_3_ × 6 H_2_O, in H_2_O mit 0,05 % BSA, Caco-2-Zellen, HepaRG-Zellen	0, 90, 128 µg/ml (0; 10,1; 14,3 µg Al/ml), 24 h		keine, keine erhöhten ROS	–	n. u.	Jalili et al. 2022
	AlCl_3_, in H_2_O, HC11-Zellen, NMuMG-Zellen (Brustepithelzelllinie)	0, 100 µM (2,7 µg Al/ml), 24 h	2,7 µg Al/ml	HC11-Zellen: Zytotoxizität nach 4 u. 7 d (Zellzahl mit Trypanblau, Annexin V/Propidiumiodid-Apoptose-Assay): 2,7 µg Al/ml: nur nach 7 d nur Zellzahl ohne Trypanblau sig. ↓	HC11-Zellen: + NMuMG-Zellen: –	n. u.	Mandriota et al. 2020
	AlCl_3_, in Zellkulturmedium, 149BR-, HF19-, MRC5-Zellen (humane Fibroblasten-Zelllinien)	0, 3, 10, 30, 100, 300, 1000 µM abgelesen aus Diagramm (0,1; 0,3; 0,8; 2,7; 8,1; 27 µg Al/ml), 24 h	ab 0,8 µg Al/ml > 2 γ-H2AX-Foci pro Zelle	n. u. (Hintergrundwert γ-H2AX-Foci pro Zelle < 2)	+	n. u.	Viau et al. 2021
DNA-Schäden (Comet, neutral)	AlCl_3_, k. A. zum Lösungsmittel, humane Lymphozyten-Zelllinie (Jurkat)	0; 0,05; 0,1; 0,5; 1; 5 mM (1,4; 2,7; 13,5; 27,0; 135 µg Al/ml), 48 h	–	keine (FACS, Permeabilitätsmessung mittels Propidiumiodid-Färbung)	–	n. u.	Caicedo et al. 2008
DNA-Schäden (Comet, alkalisch)	AlCl_3_ × 6 H_2_O, in 0,9%iger NaCl-Lösung, humane Lymphozyten, 3 Probanden (k. A. zum Geschlecht)	0, 1, 2, 5, 10, 25 µg/ml (0,1; 0,2; 0,6; 1,1; 2,8 µg Al/ml), 72 h	ab 0,2 µg Al/ml	ab 0,2 µg Al/ml (FACS, Annexin V/Propidiumiodid-Apoptose-Assay)	+	n. u.	Lankoff et al. 2006
	AlCl_3_ × 6 H_2_O, in H_2_O mit 0,05 % BSA, Caco-2-Zellen, HepaRG-Zellen	0, 90, 128 µg/ml (0; 10,1; 14,3 µg Al/ml), 5 h, 24 h	–	keine, keine erhöhten ROS	–	n. u.	Jalili et al. 2022
	AlCl_3_, in Methanol, humane Lymphozyten, 4 Probanden (♂ u. ♀)	0, 5, 10, 15, 25 µM (0,1; 0,3; 0,4; 0,7 µg Al/ml), 3 h	ab 0,1 µg Al/ml	ab 0,1 µg Al/ml MI ↓, keine Konzentrations-Wirkungs-Beziehung	+	n. u.	Lima et al. 2007
	AlCl_3_, in H_2_O, humane Neuroblastom-Zelllinien (SH-SY5Y5, SK-N-BE-2)	0, 4, 40 µM (0,1; 1,1 µg Al/ml), 1 h, 5 h	–	keine (LDH-Assay)	–	n. u.	Villarini et al. 2017
	AlCl_3_, in H_2_O, humane Brustzelllinien (MCF-10A, MCF-7, MDA-MB-231)	0, 250 µM (6,8 µg Al/ml), 24 h	–	keine (WST-1-Reduktionstest)	–	n. u.	Roszak et al. 2017
Oxidative DNA-Schäden (Comet mit FpG, alkalisch)	AlCl_3_ × 6 H_2_O, in H_2_O mit 0,05 % BSA, Caco-2-Zellen, HepaRG-Zellen	0, 90, 128 µg/ml (10,1; 14,3 µg Al/ml), 5 h (nur Caco-2-Zellen), 24 h	–	keine, keine erhöhten ROS	–	n. u.	Jalili et al. 2022
	AlCl_3_, in deionisiertem H_2_O, humane Brustzelllinien (MCF-10A, MCF-7, MDA-DB-231)	0, 250 µM (6,8 µg Al/ml), 24 h	–	keine (WST-1-Reduktionstest)	–	n. u.	Roszak et al. 2017
Oxidative DNA-Schäden (Comet-Assay mit FpG und EndoIII, alkalisch)	AlCl_3_ × 6 H_2_O, in 0,9%iger NaCl-Lösung, humane Lymphozyten, 3 Probanden (k. A. zum Geschlecht)	0, 1, 2, 5, 10, 25 µg/ml (0,1; 0,2; 0,6; 1,1; 2,8 µg Al/ml), 72 h	ab 0,2 µg Al/ml	ab 0,2 µg Al/ml (FACS, Annexin V/FITC-Apoptose-Assay)	+	n. u.	Lankoff et al. 2006
SCE	AlCl_3_, k. A. zum Lösungsmittel, humane Lymphozyten, 5 Probanden (k. A. zum Geschlecht)	0, 3 mg/ml (607 µg Al/ml), 69 h	607 µg Al/ml	607 µg Al/ml (CCPI ↓)	+	n. u.	Patel et al. 2009
	Al_2_(SO_4_)_3_, k. A. zum Lösungsmittel, humane Lymphozyten (k. A. zum Spender)	0, 10, 20 µg/ml (1,6; 3,2 µg Al/ml), k. A.	3,2 µg Al/ml	n. u.	+	n. u.	Türkez und Geyikoğlu 2011
	Al_2_(SO_4_)_3_, k. A. zum Lösungsmittel, humane Lymphozyten, je 5 ♂ u. ♀ Spender pro Altersgruppe (0–10, 21–30, 41–50 Jahre)	0, 20 µg/ml (3,2 µg Al/ml), 72 h	3,2 µg Al/ml	keine (Replikationsindex)	+	n. u.	Greim 2007; Roy et al. 1990
CA (Rearrangements)	AlCl_3_, in H_2_O, HC11-Zellen, NMuMG-Zellen (Brustepithelzelllinien)	HC11-Zellen: 0, 10, 100 µM (0,27; 2,7 µg Al/ml), 71 Wo; NMuMG-Zellen: 0, 100 µM (2,7 µg Al/ml), 38/40 Wo	HC11: ab 0,27 µg Al/ml NMuMG: bei 2,6 µg Al/ml	n. u.	+	n. u.	Mandriota et al. 2020
CA	AlCl_3_, in H_2_O, HC11-Zellen, NMuMG-Zellen (Brustepithelzelllinien)	0, 100 µM (2,7 µg Al/ml), 24 h	2,7 µg Al/ml	keine nach 4 u. 7 d (Annexin V/Propidiumiodid-Apoptose-Assay)	HC11-Zellen: + NMuMG-Zellen: –	n. u.	Mandriota et al. 2020
	AlCl_3_, k. A. zum Lösungsmittel, humane Lymphozyten, 5 Probanden (k. A. zum Geschlecht)	0, 3 mg/ml (607 µg Al/ml), 69 h	607 µg Al/ml	607 µg Al/ml (CCPI ↓), Auswertung fehlerhaft (inkl. Gaps)	+	n. u.	Patel et al. 2009
	AlCl_3_, in Methanol, humane Lymphozyten, 4 Probanden (♂ u. ♀)	0, 5, 10, 15, 25 µM (0,13; 0,3; 0,4; 0,7 µg Al/ml), 1 h, 3 h, 6 h, 28 h, 52 h	ab 0,1 µg Al/ml	ab 0,1 µg Al/ml MI ↓, Gaps + Brüche, Polyploidien, Endoreduplikationen ↑, Brüche fraglich ↑	+	n. u.	Lima et al. 2007
	AlCl_3_ × 6 H_2_O, in H_2_O, V79-Zellen	0, 10, 100, 300 µM (0,3; 2,7; 8,1 µg Al/ml), 24 h	ab 0,3 µg Al/ml	2,7 µg Al/ml: nach 3 u. 24 h: Viabilität ↓, Nekrose ↑ (Annexin V/Propidiumiodid-Apoptose-Assay), Gaps nicht bestimmt; Inkubation unter unphysiologischen Bedingungen; 3 h bei 2,7 µg Al/ml: Zellen mit multipolaren Spindeln ↑	+	n. u.	Tenan et al. 2021
	Al_2_(SO_4_)_3_, k. A. zum Lösungsmittel, humane Lymphozyten, je 5 ♂ u. ♀ Spender pro Altersgruppe (0–10, 21–30, 41–50 Jahre)	0, 20 µg/ml (3,2 µg Al/ml), 72 h	3,2 µg Al/ml	MI bei Lymphozyten der ♂ Probanden der höchsten Altersgruppe ↓	+	n. u.	Greim 2007; Roy et al. 1990
	Al_2_(SO_4_)_3_, k. A. zum Lösungsmittel, humane Lymphozyten (k. A. zum Spender)	0, 10, 20 µg/ml (1,6; 3,2 µg Al/ml), 72 h	3,2 µg Al/ml	n. u., SOD ↓, G6PDH ↓, CAT ↓, GSH ↓, k. A. zu Art der CA, nur 30 Metaphasen/Konzentration ausgezählt, invalide	+	n. u.	Türkez und Geyikoğlu 2011
	AlK(SO_4_)_2_, k. A. zum Lösungsmittel, CHL-Zellen	0, 1000 µg/ml (104,5 µg Al/ml), 48 h	–	k. A.	–	n. u.	Ishidate et al. 1988
	Al(CH_3_COO)_2_(OH) × H_2_O, in DMSO, humane Lymphozyten, 2 Probanden (♀), OECD TG 473 (von 2016)	0, 62,5–500 µg/ml (8,6–69,1 µg Al/ml), 5 h (–m. A.), 0, 7,81–31,3 µg/ml (1,1–4,3 µg Al/ml), 29 h (–m. A.), 0, 125–1000 µg/ml (17,3–138,3 µg Al/ml), 5 h (+m. A.)	–	MI ↓ > 138,3 µg Al/ml, (5 h) u. > 8,6 µg Al/ml, (29 h), Präzipitation ab 138,3 µg Al/ml (5 h) u. ab 69,2 µg Al/ml (29 h)	–	–	ECHA 2020 c
	AlNH_4_(SO_4_)_2_ in Wasser, humane Lymphozyten, 3 Probanden (♂), OECD TG 473 (Jahr n. a.)	0, 40–120 µg/ml (4,6–16,6 µg Al/ml), 3 h (+m. A.), 0, 50–120 µg/ml (6,9–16,6 µg Al/ml), 3 h (–m. A.), Untersuchung: 17 h (0, 50–200 µg/ml (6,9–27,7 µg Al/ml)), 20 h (–m. A.)	–	keine (MI), Präzipitation ab 6,9 µg Al/ml (+m. A.), ab 11,1 µg Al/ml (–m. A.) (3 h), ab 20,8 µg Al/ml (–m. A.) (20 h)	–	–	ECHA 2020 a
MN (FISH)	AlCl_3_, k. A. zum Lösungsmittel, humane Lymphozyten, 3 Probanden (♂)	0, 1, 2, 5, 10, 25 µg/ml (0,2; 0,4; 1,0; 2,0; 5,1 µg Al/ml), 72 h	ab 0,2 µg Al/ml	Apoptose ab 0,4 µg Al/ml, MNC+ u. MNC– ↑ (nur bei 1 µg Al/ml untersucht)	+	n. u.	Banasik et al. 2005
	Al_2_(SO_4_)_3_, in H_2_O, humane Lymphozyten, 2 Probanden (A u. B) (♂)	0, 500, 1000, 2000, 4000 µM (27, 54, 108, 216 mg Al/ml), 72 h	A: bei 54 mg Al/ml, 108 mg Al/ml, B: ab 27 mg Al/ml	A: ab 27 mg Al/ml (% BNC ↓), B: keine Zytotoxizität (% BNC unverändert), MNC+ ↑ bei 54 mg Al/ml u. 108 mg Al/ml (nur diese Konz. untersucht)	+	n. u.	Greim 2007; Migliore et al. 1999
MN	AlCl_3_, in Zellkulturmedium, Fibroblasten-Zelllinie 149BR	0, 3, 10, 30, 100, 300, 1000 µM abgelesen aus Diagramm (0,1; 0,3; 0,8; 2,7; 8,1; 27 µg Al/ml), 24 h	ab 0,8 µg Al/ml > 2 MN pro Zelle	keine (Hintergrundwert γ-H2AX-Foci pro Zelle < 2)	+	n. u.	Viau et al. 2021
	AlCl_3_, k. A. zum Lösungsmittel, humane Lymphozyten, 5 Probanden (k. w. A.)	0, 3 mg/ml (607 µg Al/ml), 69 h	607 µg Al/ml	607 µg Al/ml: CCPI ↓, Aneuploidie (Hyper- und Hypoploidie) ↑	+	n. u.	Patel et al. 2009
	AlCl_3_ × 6 H_2_O, in destilliertem H_2_O, humane Lymphozyten, 3 Probanden (2 ♂ u. 1 ♀)	0, 5, 10, 20 µM (0,1; 0,3; 0,5 µg Al/ml), 72 h	ab 0,1 µg Al/ml	n. u., Negativkontrolle hat hohe Anzahl an MN	+	n. u.	Paz et al. 2017
	AlCl_3_ × 6 H_2_O, in H_2_O mit 0,05 % BSA, Caco-2-Zellen, HepaRG-Zellen	0, 90, 128 µg/ml (10,1; 14,3 µg Al/ml), 24 h	–	keine (RI), keine erhöhten ROS	–	n. u.	Jalili et al. 2020
	Al_2_(SO_4_)_3_, k. A. zum Lösungsmittel, humane Lymphozyten, je 5 ♂ u. ♀ Spender pro Altersgruppe (0–10, 21–30, 41–50 Jahre)	0, 20 µg/ml (3,2 µg Al/ml), 72 h	3,2 µg Al/ml	MI bei ♂ Probanden der höchsten Altersgruppe ↓	+	n. u.	Greim 2007; Roy et al. 1990
	Al_2_(SO_4_)_3_, in deionisiertem H_2_O, humane dermale Fibroblasten u. Lymphozyten, 17 Probanden (♂ u. ♀), 64 ± 7 Jahre, R/NR	0, 1 mM (54 µg Al/ml), 48 h	54 µg Al/ml	% BNC ↓	+	n. u.	Greim 2007; Trippi et al. 2001
	Al(OH)_1,22_Cl_1,78_, in H_2_O, Reinheit > 99,7 %, humane Lymphozyten (♂), OECD TG 487 (von 2009)	0, 100–600 µg/ml (24,4–146,1 µg Al/ml), 3 h (+/–m. A.) und 24 h (–m. A.), Untersuchung: 27 h bzw. 24 h	–	keine (CBPI), bei 186 µg Al/ml Präzipitation	–	–	NOTOX B.V. 2010 a
	C_9_H_15_AlO_9_, in DMSO, humane Lymphozyten (♂), OECD TG 487 (von 2010)	0, 100–2800 µg/ml (9,2–256,8 µg Al/ml), 3 h (+/–m. A.) und 500–3000 µg/ml (45,9–275,1 µg Al/ml), 24 h (–m. A.), Untersuchung: 27 h bzw. 24 h	konzentrationsabhängig, k. w. A.	CBPI: 74–98 % bei 3000 µg/ml, 13–23 % bei 92 μg Al/l, k. w. A., pH 5,92 bei 275,1 µg Al/ml, pH 7,89 in Kontrolle, keine Präzipitation, aber ab 92 µg Al/ml leicht trüb	+	+	ECHA 2022 b
	Al_2_(OH)_0,16–0,18_(SO_4_)_2,91–2,92_, in RPMI-Medium, humane Lymphozyten (♂), OECD TG 487 (Entwurf von 2009)	0, 30–1000 µg/ml (4,8–160,4 µg Al/ml), 3 h (+/–m. A.) und 0, 50–1750 µg/ml (80,2–481,3 µg Al/ml), 24 h (–m. A.), Untersuchung: 27 h bzw. 24 h	–	CBPI ↓, Präzipitation ab 112,3 µg Al/ml (+m. A.), ab 160,4 µg Al/ml (–m. A.)	–	–	ECHA 2023 c

^a)^ Ergebnis statistisch signifikant, wenn nicht anders angegeben

Al: Aluminium; AlCl_3_: Aluminiumchlorid; Al_2_Cl_2_(OH)_2_SO_4_: Aluminiumchloridhydroxysulfat; Al(CH_3_COO)_2_(OH): Aluminiumdiacetat; AlNH_4_(SO_4_)_2_: Aluminiumammoniumdisulfat; Al(OH)_1.22_Cl_1.78_: basisches Aluminiumchlorid; Al_2_(OH)_0,16–0,18_(SO_4_)_2,91–2,92_: Aluminiumsulfat, basisch; Al_2_(SO_4_)_3_: Aluminiumsulfat; BNC: binucleated cells (zweikernige Zellen); BSA: bovines Serumalbumin; CA: Chromosomenaberrationen; Caco-2-Zellen: humane Colon-Adenokarzinom-Zellen; CAT: Katalase; CBPI: cytokinesis-block proliferation index; CCPI: cell cycle proliferative index; C_9_H_15_AlO_9_: Aluminiumlactat; Endo III: Endonuklease III; FACS: Durchflusszytometrie (Fluorescence Activated Cell Sorting); FISH: Fluoreszenz-in-situ-Hybridisierung; FpG: Formamidopyrimidin-DNA-Glycosylase; G6PDH: Glucose-6-phosphat-Dehydrogenase; γ-H2AX: phosphoryliertes Histon H2AX; GSH: Glutathion; h: Stunde(n); HepaRG-Zellen: immortalisierte humane Leber-Stamm-Zelllinie; k. (w.) A.: keine (weiteren) Angaben; LDH: Lactatdehydrogenase; MI: mitotischer Index; MN: Mikronuklei; MNC+: Zentromer-positive Mikronuklei; MNC–: Zentromer-negative Mikronuklei; n. a.: nicht angegeben; NR: Nichtraucher; n. u.: nicht untersucht; R: Raucher; RI: Replikationsindex; ROS: reaktive Sauerstoffspezies; SCE: Schwesterchromatidaustausche; SOD: Superoxiddismutase; TG: Test Guideline (Prüfrichtlinie)

#### 5.6.2 In vivo

Die Untersuchungen zur Genotoxizität in vivo mit löslichen Aluminiumverbindungen sind in Tabelle 17 aufgeführt.

**Tab. 17:** In-vivo-Studien zur Genotoxizität mit löslichen Aluminiumverbindungen

**Endpunkt**	**Testsystem**	**Expositionszeit, Substanz, Dosis, Lösungsmittel, Expositionsweg, Untersuchungszeitpunkt**	**Ergebnis^a)^**	**Zytotox.^a)^/Anmerkungen**	**Literatur**
DNA-Fragmentierung, Gehirn	Ratte, Wistar, je 6 ♂	7 d, AlCl_3_ × 6 H_2_O, 0, 150 mg/kg KG u. d (17 mg Al/kg KG u. d), 0,9 % Kochsalzlösung, i.p., untersucht nach 24 h	+	histopathologische Veränderungen, SOD ↓, GSH-Px ↓, MDA ↑	Liaquat et al. 2019
DNA-Schäden (Comet, alkalisch), Hippocampus, Cortex	Maus, ICR, je 15, k. w. A.	100 d, AlCl_3_, 0, 10, 50, 300 mg/kg KG u. d (2, 10, 61 mg Al/kg KG u. d), in Käse mit Zitronensaft, untersucht nach 24 h	+ ab 2 mg Al/kg KG	n. u., ab 2 mg Al/kg KG: MDA ↑, SOD ↓	Rui und Yongjian 2010
DNA-Schäden (Comet, alkalisch), peripheres Blut	Ratte, Sprague-Dawley, je 3 ♀	5, 10, 15 d, AlCl_3_, 0, 10 mg Al/kg KG u. d, k. A. zum Lösungsmittel, Gavage	+ (10, 15 d)	keine (Trypanblau) aber starke Fragmentierung (clouds) nach 10 u. 15 d, anästhesiert mit Halothan, Studie zweifelhaft valide	García-Alegría et al. 2020
DNA-Schäden (Comet, alkalisch), Leber, Zwölffingerdarm, Milz, Knochenmark, Nieren, Blut	Ratte, Sprague Dawley, je 5 ♂	dreimalig (0, 24, 45 h), AlCl_3_ × 6 H_2_O, 0, 25 mg/kg KG u. d (3 mg Al/kg KG u. d), k. A. zum Lösungsmittel, Gavage, untersucht nach 3 h	–	keine im Knochenmark (PCE/NCE-Verhältnis), in Leber, Zwölffingerdarm, Milz, Nieren, Blut n. u.	Jalili et al. 2020
oxidative DNA-Schäden (8-OHdG), mitochondriale DNA aus Hippocampus, Cortex	Maus, ICR, je 15, k. w. A.	100 d, AlCl_3_, 0, 10, 50, 300 mg/kg KG u. d (2, 10, 61 mg Al/kg KG u. d), in Käse mit Zitronensaft, untersucht nach 24 h	+ ab 2 mg Al/kg KG	n. u., ab 2 mg Al/kg KG: MDA ↑, SOD ↓	Rui und Yongjian 2010
oxidative DNA-Schäden (8-OHdG), Herz	Ratte, Wistar, je 7 ♂	120 d, AlCl_3_, 0; 0,02; 0,1; 50; 200 mg/kg KG u. d (0,004; 0,02; 10; 40 mg Al/kg KG u. d), destilliertes Wasser, Gavage	+ ab 0,02 mg Al/kg KG u. d	ab 0,02 mg Al/kg KG (strukturelle Veränderungen der Kardiomyozyten)	Novaes et al. 2018
oxidative DNA-Schäden (Comet mit FpG, alkalisch), Leber, Zwölffingerdarm, Milz, Knochenmark, Nieren, Blut	Ratte, Sprague-Dawley, je 5, k. w. A.	dreimalig (0, 24, 45 h), AlCl_3_ × 6 H_2_O, 0, 25 mg/kg KG (3 mg Al/kg KG), k. A. zum Lösungsmittel, Gavage, untersucht nach 3 h	–	keine im Knochenmark (PCE/NCE-Verhältnis), in Leber, Zwölffingerdarm, Milz, Nieren, Blut n. u.	Jalili et al. 2020
SCE, Knochenmark	Maus, Swiss, je 5 ♂	einmalig, Al_2_(SO_4_)_3_ × 18 H_2_O, 0, 100, 200, 400 mg/kg KG (8, 16, 32 mg Al/kg KG), Kochsalzlösung, Gavage	+ ab 16 mg Al/kg KG	Proliferationsindex bei allen Konzentrationen unverändert	Dhir et al. 1993; Greim 2007
CA, Knochenmark	Ratte, Albino, je 5 ♂, k. w. A.	7, 14, 21 d, Al_2_(SO_4_)_3_ × 18 H_2_O, 0, 212, 265, 353, 530, 1060, 2120 mg/kg KG u. d (17, 21, 29, 43, 86, 172 mg Al/kg KG u. d), deionisiertes Wasser, Gavage, untersucht nach 24 h	+ 7 d: ab 17 mg Al/kg KG, 14 d: ab 86 mg Al/kg KG, 21 d: ab 21 mg Al/kg KG	ab 17 mg Al/kg KG (Mitoseindex sporadisch stat. sign. ↓ (7, 14, 21 d)), keine Positivkontrolle	Greim 2007; Roy et al. 1991
	Maus, Swiss, je 5 ♂	einmalig, Al(CH_3_COO)_2_(OH) × H_2_O, 0, 50, 100, 150 mg/kg KG (12, 25, 37 mg Al/kg KG), destilliertes Wasser, i.p., untersucht nach 24, 48, 72 h	+ ab 12 mg Al/kg KG	ab 25 mg Al/kg KG (24 h) bzw. bei 37 mg/kg KG (48 h) (Mitoseindex ↓), LD_50_ nach 24 h: 1150 mg/kg KG	D’Souza et al. 2014
		7 d, Al(CH_3_COO)_2_(OH) × H_2_O, 0, 50 mg/kg KG u. d (12 mg Al/kg KG u. d), destilliertes Wasser, i.p., untersucht nach 24, 48, 72 h	+	Mitoseindex ↓	
	Maus, k. w. A.	einmalig, AlCl_3_, 0; 0,01; 0,05; 0,1 M 1 ml/Maus (ca. 44, 222, 443 mg/kg KG (EFSA 2008); 9; 44,9; 89,6 mg Al/kg KG), k. A. zum Lösungsmittel, i.p.	+	nicht dosisabhängig	ATSDR 2008; EFSA 2008
MN, peripheres Blut	Ratte, Sprague-Dawley, je 3 ♀	5, 10, 15 d, AlCl_3_, 0, 10 mg Al/kg KG u. d, k. A. zum Lösungsmittel, Gavage	+ (10, 15 d)	PCE/NCE-Verhältnis n. u., das Fehlen/geringe Vorkommen von MN in Kontrolle bzw. behandelten Proben ist zweifelhaft, Dokumentationsmängel: z. B. Widersprüche zwischen Tabelle und Abbildung, Studie insgesamt zweifelhaft valide	García-Alegría et al. 2020
MN, Knochenmark, Colon	Ratte, Sprague-Dawley, je 5, k. w. A.	dreimalig (0, 24, 45 h), AlCl_3_ × 6 H_2_O, 0, 25 mg/kg KG u. d (3 mg Al/kg KG u. d), Wasser mit 0,05 % BSA, Gavage, untersucht nach 3 h	-	Knochenmark: keine (PCE/NCE-Verhältnis), Colon: kein Anstieg von mitotischen und apoptotischen Zellen (k. w. A.), keine validierte Methode für MN	Jalili et al. 2020
MN, Knochenmark	Maus, NMRI, je 5 ♂ u. 5 ♀ OECD TG 474	2 d, Al_2_(OH)_5_Cl, 0, 2000 mg/kg KG u. d (ca. 619 mg Al/kg KG und d), gelöst in deionisiertem Wasser, Reinheit 100 %, Gavage, untersucht nach 24 h	–	keine (PCE/Gesamtzahl Erythrozyten)	Hoechst Marion Roussel Deutschland GmbH 1999 b
	Maus, Swiss, je 4 ♂ u. 4 ♀	einmalig, AlCl_3_ × 6 H_2_O, 0, 49, 98, 161 mg/kg KG (5, 11, 18 mg Al/kg KG), destilliertes Wasser, oral (k. w. A.), untersucht nach 24 h	+ ab 5 mg Al/kg KG	keine im Knochenmark (PCE/NCE-Verhältnis), ab 5 mg/kg KG: histopathologische Effekte in Leber, Nieren, Studie zweifelhaft valide	Paz et al. 2017, 2018
	Maus, Swiss, je 5, k. w. A.	einmalig, Al(CH_3_COO)_2_(OH) × H_2_O, 0, 50, 100, 150 mg/kg KG (12, 25, 37 mg Al/kg KG), destilliertes Wasser, i.p., untersucht nach 24, 48, 72 h	–	bei 37 mg Al/kg KG (PCE/NCE-Verhältnis verändert)	D’Souza et al. 2014
		7 d, Al(CH_3_COO)_2_(OH) × H_2_O, 0, 50 mg/kg KG u. d (7 mg Al/kg KG u. d), destilliertes Wasser, i.p., untersucht nach 24, 48, 72 h	+	PCE/NCE-Verhältnis verändert	
	Maus, Swiss, je 6 ♂ u. ♀	2 d, Al_2_(SO_4_)_3_ × 18 H_2_O, 0, 250, 500 mg/kg KG u. d (20, 40 mg Al/kg KG u. d), gelöst in Kochsalzlösung, i.p., untersucht nach 24 h	+ ab 20 mg Al/kg KG	keine (PCE/NCE-Verhältnis)	Greim 2007; Roy et al. 1992
MN, Hepatozyten	Ratte, Sprague-Dawley, je 5 ♂	30 d, AlCl_3_, 0, 34 mg/kg KG u. d (7 mg Al/kg KG u. d), k. A. zum Lösungsmittel, oral, k. w. A.	+	Schädigung der Leberzellen, ALP, AST, ALT, LDH im Serum ↑, keine Positivkontrolle	Türkez et al. 2010
	Ratte, Sprague-Dawley, je 6 ♂	10 Wo, AlCl_3_, 0, 5 mg/kg KG u. d (1 mg Al/kg KG u. d), k. A. zum Lösungsmittel, i.p.	+	Schädigung der Leberzellen, ALP, AST, ALT, LDH im Serum ↑	Geyikoglu et al. 2013
	Ratte, Sprague-Dawley, je 4 ♂	4 d, AlCl_3_, 0, 5 mg/kg KG u. d (1 mg Al/kg KG u. d), k. A. zum Lösungsmittel, i.p.	+	n. u.	Türkez et al. 2013
MN, fetale Leber	Maus, Swiss, je 3 ♀: 4 Feten pro behandeltem ♀, 14. Gestationstag	einmalig, Al(CH_3_COO)_2_(OH) × H_2_O, 0, 50, 100, 150 mg/kg KG (12, 25, 37 mg Al/kg KG), destilliertes Wasser, i.p., untersucht nach 24 h	+ ab 12 mg Al/kg KG	nicht dosisabhängig im fetalen Knochenmark ab 12 mg Al/kg KG (PCE/NCE-Verhältnis), in fetaler Leber n. u.	D’Souza et al. 2014

^a)^ Ergebnis statistisch signifikant, wenn nicht anders angegeben

Al: Aluminium; Al(CH_3_COO)_2_(OH): Aluminiumdiacetat; AlCl_3_: Aluminiumchlorid; Al_2_(OH)_5_Cl: Aluminiumchlorhydrat; ALP: alkalische Phosphatase; Al_2_(SO_4_)_3_: Aluminiumsulfat; ALT: Alaninaminotransferase; AST: Aspartataminotransferase; BSA: bovines Serumalbumin; CA: Chromosomenaberrationen; d: Tag(e); FpG: Formamidopyrimidin-DNA-Glycosylase; GSH-Px: Glutathion-Peroxidase; h: Stunde(n); i.p. intraperitoneal; k. (w.) A.: keine (weiteren) Angaben; LDH: Lactatdehydrogenase; MDA: Malondialdehyd; MI: mitotischer Index; MN: Mikronuklei; NCE: normochromatische Erythrozyten; 8-OHdG: 8-Oxo-2′-desoxyguanosin; PCE: polychromatische Erythrozyten; SCE: Schwesterchromatidaustausche; SOD: Superoxiddismutase; TG: Test Guideline (Prüfrichtlinie); Wo: Woche(n)

##### 5.6.2.1 Indikatortests

Eine Untersuchung zur DNA-Schädigung zeigte im Gehirn von Wistar-Ratten nach i.p. Gabe eine DNA-Fragmentierung bei 17 mg Al/kg KG und Tag bei gleichzeitiger Zytotoxizität (Liaquat et al. 2019).

Eine mittels Comet-Assay bestimmte DNA-Schädigung trat nach oraler Gabe ab 10 mg Al/kg KG und Tag im peripheren Blut von Sprague-Dawley-Ratten (García-Alegría et al. 2020) und ab 2 mg Al/kg KG und Tag im Gehirn von ICR-Mäusen auf, ohne dass Zytotoxizität untersucht wurde (Rui und Yongjian 2010). In der Studie an der Ratte betrug die Zellviabilität (Trypanblaufärbung) 96 % (García-Alegría et al. 2020). Allerdings zeigten sich nach 10 und 15 Tagen drei bzw. 73 „hedgehogs“ pro 100 Comets (in der Studie als Clouds bezeichnet), was auf Apoptose und somit Zytotoxizität hinweist. Weiterhin hat diese Publikation massive orthografische Mängel und ist deshalb zweifelhaft valide.

Nach dreimaliger Schlundsondengabe von 3 mg Al/kg KG und Tag an Sprague-Dawley-Ratten wurde mittels Comet-Assay keine DNA-Schädigung in Leber, Zwölffingerdarm, Milz, Knochenmark, Niere und Blut beobachtet. Zytotoxizität wurde nur im Knochenmark untersucht und trat nicht auf (Jalili et al. 2020).

In zwei Untersuchungen an ICR-Mäusen und Wistar-Ratten wurde nach oraler Gabe von Aluminiumchlorid die Induktion oxidativer DNA-Basenschäden nachgewiesen (Novaes et al. 2018; Rui und Yongjian 2010). Hierfür waren Kardiomyozyten von Wistar-Ratten mit Effekten ab 0,02 mg Al/kg KG und Tag nach 120 Tagen am sensitivsten bei gleichzeitig auftretender struktureller Veränderung, was als toxische Wirkung auf das Herz beurteilt werden kann (Novaes et al. 2018). Nach oraler Gabe von 3 mg Al/kg KG und Tag als Aluminiumchlorid traten in Leber, Zwölffingerdarm, Milz, Knochenmark, Niere und Blut von Sprague-Dawley-Ratten keine oxidativen DNA-Schäden auf (Jalili et al. 2020).

Aluminiumsulfat führte bei Mäusen nach einer einmaligen Schlundsondengabe ab 16 mg Al/kg KG und Tag zu einer erhöhten Häufigkeit von Schwesterchromatidaustauschen im Knochenmark. Der Proliferationsindex, als Maß für die Zytotoxizität, war bei allen Konzentrationen unverändert (Dhir et al. 1993; Greim 2007).

##### 5.6.2.2 Chromosomenaberrationen

An Ratten konnte eine klastogene Wirkung im Knochenmark nach siebentägiger Schlundsondengabe von Aluminiumsulfat ab 17 mg Al/kg KG und Tag beobachtet werden. Es trat gleichzeitig Zytotoxizität in Form eines sporadisch statistisch signifikant verringerten Mitoseindex auf. Nach 14- und 21-tägiger Gabe kam es erst ab höheren Dosierungen zu Chromosomenaberrationen (Roy et al. 1991). Die Auswertung erfolgte nicht blind, was zu einer Verzerrung der Bewertung führen kann. Die statistische Analyse erfolgte nach einer Kombination aller Kategorien abnormaler Zellen. Die Autoren des REACH-Registrierungsdossiers sehen die Studie als nicht valide an (ECHA 2023 c).

Eine erhöhte Anzahl an Chromosomenaberrationen im Knochenmark trat nach einmaliger und wiederholter siebentägiger i.p. Gabe an Swiss-Mäuse ab 12 mg Al/kg KG und Tag, gegeben als Aluminiumdiacetat, auf. Diese Dosis führte nach sieben Tagen auch zu Zytotoxizität. Nach einmaliger Gabe begann die Zytotoxizität bei 25 mg/kg KG, gemessen anhand eines verminderten Mitoseindexes (D’Souza et al. 2014). Aufgrund der i.p. Gabe ist diese Studie sowie auch der in EFSA (2008) und ATSDR (2008) genannte Chromosomenaberrationstest an Mäusen nur begrenzt bewertungsrelevant. Zudem fehlte eine mitlaufende Positivkontrolle.

##### 5.6.2.3 Mikronuklei

Im peripheren **Blut** von Sprague-Dawley-Ratten wurde nach zehntägiger Schlundsondengabe von 10 mg Al/kg KG und Tag an fünf Tagen pro Woche, gegeben als Aluminiumchlorid, eine signifikante Zunahme (p < 0,05) an Mikronukleus-haltigen Zellen (9,3/2000 mit 1 Mikronukleus) gezeigt, die sich nach 15 Tagen verstärkte (García-Alegría et al. 2020). Eine Bestimmung der Zytotoxizität wurde nicht durchgeführt. Im Comet-Assay an Leukozyten in derselben Publikation ergab sich 96 % Zellviabilität. Allerdings zeigten sich nach 10 und 15 Tagen drei bzw. 73 „hedgehogs“ pro 100 Comets (in der Studie als Clouds bezeichnet), was auf eine zytotoxische Wirkung hinweist. Weiterhin enthält die Veröffentlichung orthografische Mängel und Unstimmigkeiten, so stimmt die in Tabelle 2 angegebene Häufigkeit von Zellen mit einem Mikronukleus/2000 Zellen nach 15-tägiger Gabe nicht mit dem Wert in der Abbildung 2 überein. Das Fehlen bzw. geringe Vorkommen von Mikronuklei in Kontrolle bzw. behandelten Proben ist unplausibel. Insgesamt ist die Studie zweifelhaft valide.

Negative Befunde zeigten sich im **Knochenmark** von Sprague-Dawley-Ratten nach dreimaliger Schlundsondengabe von 3 mg Al/kg KG und Tag, gegeben als Aluminiumchlorid (Jalili et al. 2020) sowie von NMRI-Mäusen nach zweitägiger Schlundsondengabe von ca. 619 mg Al/kg KG und Tag, verabreicht als Aluminiumchlorhydrat in einer Untersuchung nach OECD-Prüfrichtlinie 474 (Hoechst Marion Roussel Deutschland GmbH 1999 b). Bei beiden Untersuchungen trat anhand des Verhältnisses von PCE (polychromatische Erythrozyten) zu NCE (normochromatische Erythrozyten) keine Zytotoxizität auf.

Eine Zunahme an Mikronuklei im Knochenmark ab der geringsten eingesetzten Dosis, aber ohne Dosis-Wirkungs-Beziehung, zeigte sich nach einmaliger oraler Gabe (k. w. A.) von 5, 11 oder 18 mg Al/kg KG und Tag, gegeben als Aluminiumchlorid, an jeweils vier männlichen bzw. weiblichen Swiss-Mäusen. Das PCE/NCE-Verhältnis zeigte keine Zytotoxizität an. In der höchsten Dosisgruppe traten jedoch statistisch signifikante Verminderungen des relativen Nieren- und Magengewichtes der männlichen Tiere sowie statistisch signifikante Effekte in Leber (sinusoidale Blutungen), Magen (eosinophile und neutrophile Entzündungszellen) und der Niere (mononukleäre Entzündungszellen und hydropische Degeneration) auf. Vier von acht Tieren der niedrigsten Dosisgruppe (k. A. zum Geschlecht) ließen statistisch signifikante Effekte in der Leber erkennen (vakuoläre Degenerationen) (Paz et al. 2017, 2018). Die histopathologischen Effekte sowie die Abnahme der relativen Organgewichte in der höchsten Dosisgruppe deuten darauf hin, dass bereits nennenswerte Toxizität auftritt. Die Autoren weisen nur im Text darauf hin, dass die histopathologischen Effekte auch in der Kontrollgruppe auftraten, eine genauere Beschreibung fehlt jedoch und in den Tabellen zu den histopathologischen Befunden in Magen, Leber und Niere der behandelten Tiere sind die Kontrollgruppen nicht aufgeführt. Ein genauer Vergleich der Inzidenzen der Kontrollgruppe mit der Gruppe der mit Aluminiumchlorid behandelten Tiere sowie ein Vergleich der Geschlechter ist somit mit den in der Veröffentlichung dargestellten Daten nicht möglich. Insgesamt ist die Aussagekraft dieser Studie stark eingeschränkt. Insbesondere das Fehlen detaillierter Daten der Kontrolltiere entspricht nicht dem wissenschaftlichen Standard.

Mit Swiss-Mäusen liegen Studien mit i.p. Gabe vor. Nach einmaliger Gabe von bis zu 37 mg Al/kg KG, gegeben als Aluminiumdiacetat, trat bei der höchsten Dosisgruppe Zytotoxizität (PCE/NCE-Verhältnis) auf, jedoch wurde keine erhöhte Mikronukleushäufigkeit beobachtet. Nach siebentägiger Gabe von 12 mg Al/kg KG und Tag stieg die Häufigkeit an Mikronuklei bei gleichzeitiger Zytotoxizität an (D’Souza et al. 2014). In einer weiteren Untersuchung trat nach zweitägiger Gabe ab 20 mg Al/kg KG und Tag, gegeben als Aluminiumsulfat, ohne Zytotoxizität (PCE/NCE-Verhältnis) eine erhöhte Mikronukleushäufigkeit auf (Greim 2007; Roy et al. 1992). Aufgrund der i.p. Gabe sind die Studien nur begrenzt bewertungsrelevant.

In Sprague-Dawley-Ratten war nach oraler Gabe (k. w. A.) von 7 mg Al/kg KG und Tag, verabreicht als Aluminiumchlorid, eine Zunahme (p < 0,05) an Mikronuklei in **Hepatozyten **(1,54 ± 0,63 im Vergleich zur Kontrolle 0,38 ± 0,08) festzustellen. Die Leberenzyme im Serum waren erhöht und die Lebern zeigten Schäden wie sinusoidale Erweiterungen, Stauungen der Zentralvene, Lipidansammlungen und Lymphozyteninfiltrationen (Türkez et al. 2010), was als Zeichen einer starken zytotoxischen Wirkung gewertet werden kann. Die Aussagekraft für eine klastogene Wirkung ist somit vermindert.

Zwei weitere Mikronukleustests an Hepatozyten ergaben positive Ergebnisse bei gleichzeitiger bzw. nicht untersuchter Zytotoxizität nach bis zu zehnwöchiger i.p. Gabe von 1 mg Al/kg KG, als Aluminiumchlorid, an Ratten (Geyikoglu et al. 2013; Türkez et al. 2013). Die Mikronukleushäufigkeit in der fetalen Leber stieg, ebenfalls bei gleichzeitiger Zytotoxizität im fetalen Knochenmark, nach einmaliger i.p. Gabe an trächtige Swiss-Mäuse am 14. Gestationstag an (D’Souza et al. 2014). Aufgrund der i.p. Gabe sind die Studien nur begrenzt bewertungsrelevant.

Negative Befunde im **Colon** von Sprague-Dawley-Ratten wurden nach dreimaliger Gabe per Schlundsonde von 3 mg Al/kg KG und Tag, als Aluminiumchlorid, erhalten. Es wurde kein Anstieg von mitotischen oder apoptotischen Zellen (k. w. A.) beobachtet (Jalili et al. 2020). Die Methode ist nicht validiert.

##### 5.6.2.4 Fazit

In vivo lassen sich in validen Untersuchungen DNA-Schäden, oxidative Basenschäden sowie die Bildung von Chromosomenaberrationen und Mikronuklei meist nur in Anwesenheit oder nahe zytotoxischer Dosen beobachten, vereinzelt treten Befunde ohne nachgewiesene Zytotoxizität auf. Die einzige In-vivo-Studie nach OECD-Prüfrichtlinie 474 (Hoechst Marion Roussel Deutschland GmbH 1999 b) ist negativ. Zytotoxizität kann zur Entgleisung zellulärer Mechanismen führen, wodurch wichtige Schutzwirkungen gegen Genotoxizität vermindert sind und dadurch genotoxische Effekte ermöglicht werden.

### 5.7 Kanzerogenität

#### 5.7.1 Kurzzeitstudien

##### Zelltransformationstests

Aluminiumchlorid und Aluminiumsulfat führten bei mit Adenoviren (SA7) behandelten embryonalen Zellen des Syrischen Hamsters zu keiner Erhöhung der Transformationshäufigkeit. Auch mit der Mausfibroblasten-Zelllinie C3H10 T1/2F war die Transformationshäufigkeit durch Aluminiumchlorid bis zur Zytotoxizität nicht statistisch signifikant erhöht (Greim 2007).

Folgende Studien sind neu hinzugekommen:

Die Untersuchung einer nicht malignen humanen Brustepithelzelllinie (MCF10A) zeigte einen Verlust der Kontaktinhibition nach sechswöchiger Inkubation mit 100 µM Aluminiumchlorid sowie eine sichtbare Induktion des „anchorage-independent growth“ nach neunwöchiger Inkubation mit 100 oder 300 µM Aluminiumchlorid. Die Autoren bewerten dies als eine beginnende maligne Transformation der Zellen (Sappino et al. 2012).

In einer nicht malignen humanen Brustepithelzelllinie (NMuMG) führte die vier Monate lange Inkubation mit 100 µM Aluminiumchlorid zu einem morphologisch veränderten Zellwachstum. In einem weiteren Versuchsansatz wurden die NMuMG-Zellen zunächst sechs bzw. acht Monate lang mit 100 µM Aluminiumchlorid inkubiert und anschließend s.c. in die Flanke von sechs Wochen alten weiblichen NSG-, NOD SCID- oder Swiss-Nacktmäusen (immundefiziente Stämme) injiziert. Die NSG-Mäuse entwickelten an der Injektionsstelle Tumore sowohl bei den mit Aluminiumchlorid vorinkubierten NMuMG-Zellen, als auch bei den nur mit Wasser vorinkubierten NMuMG-Zellen, welche als Kontrolle dienten. Allerdings waren die Tumore der Tiere, denen mit Aluminiumchlorid vorinkubierte NMuMG-Zellen injiziert wurden, statistisch signifikant größer und metastasierten häufiger in die Lunge. Bei NOD SCID- und Swiss-Nacktmäusen traten ebenfalls Tumore auf, ähnlich denen der NSG-Mäuse, jedoch nicht bei Injektion der Kontroll-Zellen. Eine vollständige Exom-Sequenzierung ergab in den mit Aluminiumchlorid behandelten NMuMG-Zellen Mutationen, die u. a. die Zellproliferation, Migration oder Metastasierung betrafen (Mandriota et al. 2016).

Die Übertragbarkeit der zuvor beschriebenen Studien auf die In-vivo-Situation ist kritisch zu sehen, da immundefiziente Mausstämme verwendet wurden. Zudem ist die Konzentration von 100 µM Aluminiumchlorid sehr hoch und führt vermutlich zu Ausflockungen, was eventuell einen Einfluss auf die Konzentrationen von Proteinen und essenziellen Metallionen im Kulturmedium hatte. Es ist plausibel, dass dadurch ein gewisser Selektionsdruck auf die Zellen ausgeübt wird (SCCS 2021).

In einer neueren Untersuchung derselben Arbeitsgruppe wurde die Brustepithelzelllinie HC11 der Maus 70 Wochen lang mit 10 µM oder 100 µM Aluminiumchlorid inkubiert. Es zeigte sich ein Verlust der Kontaktinhibition, eine konzentrationsabhängige Zunahme des „anchorage-independent growth“ sowie vermehrte chromosomale Rearrangements. Eine 24-stündige Inkubation mit 100 µM Aluminiumchlorid führte zu Chromosomenbrüchen, -fragmentierung und einem Anstieg an phosphoryliertem γ-H2AX (siehe Abschnitt 5.6.1.2). In einem weiteren Versuchsansatz wurden mit 10 µM bzw. 100 µM Aluminiumchlorid 71 Wochen lang vorinkubierte HC11-Zellen s.c. in die Flanke von immunkompetenten BALB/cByJ-Mäusen injiziert. Bei neun und acht (10 µM bzw. 100 µM) von zehn Tieren entwickelten sich Tumore an der Injektionsstelle. Zwei Tiere der 10-µM-Gruppe hatten Metastasen in der Lunge. Vier weiteren sechs Wochen alten BALB/cByJ-Mäusen wurden HC11-Zellen, welche 71 Wochen lang mit 10 µM Aluminiumchlorid inkubiert wurden, in das Brustfettpolster injiziert. Drei von vier Tieren entwickelten Tumore an der Injektionsstelle. Die Kontrolltiere, denen mit Wasser vorbehandelte HC11-Zellen injiziert wurden, hatten keine Tumore (Mandriota et al. 2020). Nach Aussage der Autoren tritt bei den vier bis sieben Tage lang mit 100 µM Aluminiumchlorid behandelten HC11-Zellen zwar eine leichte Verminderung der Proliferation, aber keine erkennbare Zytotoxizität (gemessen über einen Annexin V/Propidiumiodid-Apoptose-Assay) auf. Die sehr lange Inkubationszeit von 71 Wochen könnte zu einem Selektionsprozess der Zellen führen, wodurch nur jene Zellen überleben, die Aluminiumchlorid tolerieren. Insgesamt ist das verwendete Modell für toxikologische Untersuchungen weder gut etabliert noch validiert (SCCS 2021).

#### 5.7.2 Langzeitstudien

Es wurden seit Erscheinen der Begründung von 2007 keine neuen Untersuchungen durchgeführt. Neu aufgenommen wurde im Folgenden eine Trinkwasserstudie an Ratten (Schroeder und Mitchener 1975). Der Vollständigkeit halber werden hier zur Bewertung der Kanzerogenität nochmals alle Studien beschrieben.

##### 5.7.2.1 Fütterung

Das kanzerogene Potenzial von Aluminiumkaliumdisulfat (0; 85; 212,5; 425 oder 850 mg Al/kg KG und Tag) wurde in einer 20-monatigen Studie an je 60 männlichen und weiblichen B6C3F1-Mäusen pro Dosisgruppe untersucht. Die Inzidenz hepatozellulärer Karzinome zeigte eine dosisabhängige, in der obersten Dosisgruppe statistisch signifikante, Abnahme. Es ergibt sich aus der Studie kein Hinweis auf ein kanzerogenes Potenzial (Greim 2007). Diese Studie liegt nur als Zusammenfassung vor und es ist nicht klar, welche Organe untersucht wurden. Sie ist daher als nicht ausreichend anzusehen, um die kanzerogene Wirkung von Aluminium zu bewerten.

##### 5.7.2.2 Trinkwasser

An je 54 männliche und weibliche Swiss-Mäuse (Charles River SD) wurde 0 oder 5 mg Al/kg KG und Tag (als Aluminiumkaliumdisulfat) lebenslang (k. w. A.) mit dem Trinkwasser verabreicht. Die Häufigkeiten der lymphatischen Leukämien sowie der multiplen Tumoren (k. w. A.) waren statistisch signifikant erhöht, lagen aber im Rahmen der Spontaninzidenzen bei CD-1-Mäusen. Diese Studie kann aufgrund der fehlenden oder sehr kurz gehaltenen Dokumentation zu Studiendurchführung nicht zur Bewertung herangezogen werden (Greim 2007).

Gruppen von jeweils 52 männlichen und 52 weiblichen Long-Evans (BLU:LE)-Ratten erhielten 0 oder 5 mg Al/kg KG und Tag (als Aluminiumkaliumdisulfat) lebenslang mit dem Trinkwasser (k. w. A.). Nach dem natürlichen Tod wurden sie gewogen, makroskopische Läsionen festgestellt und Herz, Lunge, Leber, Niere und Milz mikroskopisch untersucht. Bei den männlichen Tieren war das Körpergewicht ab einem Alter von einem Jahr statistisch signifikant erhöht. Die Lebensdauer wurde durch Aluminiumkaliumdisulfat nicht beeinflusst. Es wurde nur etwa die Hälfte der Tiere autopsiert, der Rest starb an einer Lungeninfektion. Aluminiumkaliumdisulfat verursachte eine erhöhte Inzidenz (p < 0,005) von makroskopisch befundeten Tumoren bei männlichen Ratten (13/25; Kontrolle 4/26). Die Inzidenz an malignen Tumoren war bei den männlichen Tieren leicht erhöht, allerdings ohne statistische Signifikanz (Schroeder und Mitchener 1975). Die Tumortypen wurden in dieser Studie nicht aufgeschlüsselt, es erfolgte lediglich eine Unterscheidung in gutartig und bösartig. Zu Art und Lokalisation der Tumoren liegen keine Informationen vor. Die Studie kann daher nicht zur Bewertung herangezogen werden.

##### 5.7.2.3 Subkutan

Je 30 weiblichen OF1-Mäusen wurde einmal wöchentlich, vier Monate lang, je 5 mmol eines Eisen-ATP-Komplexes (FeATP) oder eines Aluminium-ATP-Komplexes (AlATP) s.c. injiziert. Es zeigten sich an der Injektionsstelle Tumoren, sowie Tumoren der Glandulae parotis und Glandula submandibularis in den Aluminium- und Eisen-ATP-behandelten Gruppen. Da nicht unterschieden wurde, welche der Tumoren bei Eisen-ATP- bzw. Aluminium-ATP-behandelten Mäusen auftraten, kann diese Studie nicht zur Bewertung herangezogen werden (Greim 2007).

##### 5.7.2.4 Fazit

Es liegen keine validen bewertungsrelevanten Studien am Tier vor, die Hinweise auf eine kanzerogene Wirkung von löslichen Aluminiumverbindungen geben.

## 6 Bewertung

Kritische Effekte sind Augenreizwirkung, Lungen- und Neurotoxizität beim Menschen.

**MAK-Wert. **Die Studien zur Augenreizwirkung beim Tier (Abschnitt 5.3.2) zeigen deutliche Unterschiede in der lokalen Wirkung der verschiedenen löslichen Aluminiumverbindungen. Während Aluminiumchlorid als ätzend am Auge bewertet ist, hat Aluminiumchlorhydrat allenfalls eine minimale Reizwirkung am Auge und ist als nicht reizend bewertet. Dies spiegelt sich in den Untersuchungen mit wiederholter inhalativer Exposition von Ratten wider (Abschnitt 5.2.1) und fließt in die MAK-Wert-Ableitung ein, bei der ein MAK-Wert ausgehend von den Daten zu Aluminiumchlorid als Worst Case für die deutlich bis stark augenreizenden Verbindungen (CLP-Einstufungen Kategorie 1 und 2) festgesetzt wird. Aus den Daten zu Aluminiumchlorhydrat wird ein separater MAK-Wert abgeleitet, der für die ebenfalls nach CLP als nicht reizend bewerteten Verbindungen gilt.

**MAK-Wert (A-Fraktion) **für **Aluminiumchlorid**, **Aluminiumchlorid, basisch**, **Aluminiumchloridhydroxysulfat**, **Aluminiumcitrat**, **Aluminiumdiacetat**, **Aluminiumlactat**, **Aluminiumnitrat**, **Aluminiumsulfat**

Die niedrigste LOAEC für lösliche Aluminiumverbindungen resultiert aus der 13-wöchigen Inhalationsstudie an Wistar-Ratten (OECD-Prüfrichtlinie 413) mit Aluminiumchlorid und beträgt 0,1 mg AlCl_3_/m^3^ (0,02 mg Al/m^3^). Bei dieser Konzentration zeigen sich die ersten Effekte der Lungentoxizität in Form von erhöhter ALP und Neutrophilenzahl in der BALF (BASF SE 2019 a). Aufgrund der leichten Effekte bei der LOAEC kann eine NAEC von 0,007 mg Al/m^3^ (LOAEC/3) abgeleitet werden. Die Extrapolation der Daten aus Tierversuchen auf den Menschen (1:2) und unter Einbezug der möglichen Zunahme der Effekte bei chronischer Exposition (1:2) und dem erhöhten Atemvolumen (1:2) ergibt eine Konzentration von 0,000875 mg Al/m^3^, was nach dem Preferred Value Approach einem MAK-Wert von 0,0005 mg Al/m^3^ entspräche. Der Vergleich der Ergebnisse der 13-Wochen-Inhalationsstudie (Aerosol aus 0,2%iger AlCl_3_-Lösung) und der 14-Tage-Vorstudie (Aerosol aus 6%iger Aluminiumchlorid-Lösung, BASF SE 2019 b) zeigt eine Zunahme der Wirkstärke mit der Konzentration von Aluminiumchlorid in der Lösung. Aufgrund der Unsicherheit bezüglich einer Wirkungsverstärkung bei einer Exposition gegen die unverdünnte Reinsubstanz wird der MAK-Wert auf 0,0002 mg Al/m^3^ (A-Fraktion) festgesetzt. Dieser MAK-Wert gilt für Aluminiumchlorid, sowie Aluminiumchlorid, basisch, Aluminiumchloridhydroxysulfat, Aluminiumcitrat, Aluminiumdiacetat, Aluminiumlactat, Aluminiumnitrat und Aluminiumsulfat, da diese Substanzen als augenreizend oder irreversibel augenschädigend (siehe Tabelle 2) bewertet wurden. Aufgrund der starken Hygroskopie ist nicht mit einer Exposition gegen wasserfreies Aluminiumchlorid zu rechnen.

**MAK-Wert (A-Fraktion) **für **Aluminiumchlorhydrat (Dialuminiumchloridpentahydroxid)**, **Aluminiumammoniumdisulfat**, **Aluminiumkaliumdisulfat**

Für das am Kaninchenauge allenfalls leicht reizend wirkende Aluminiumchlorhydrat wird der MAK-Wert aus der sechsmonatigen Inhalationsstudie an F344-Ratten abgeleitet (Steinhagen et al. 1978). Nach Exposition gegen die niedrigste Konzentration von 0,25 mg Aluminiumchlorhydrat/m^3^ (0,08 mg Al/m^3^) als Staub kam es bei einem Tier zu Mikrogranulomen in den peribronchialen Lymphknoten und einem leichten Anstieg alveolärer Makrophagen in der Lunge, so dass diese Konzentration von der Kommission als beginnender Effekt und als LOAEC bewertet wurde (siehe Abschnitt 5.2.1). Ausgehend von dieser LOAEC kann eine NAEC von 0,027 mg Al/m^3^ (LOAEC/3) abgeleitet werden. Die Extrapolation der Daten aus Tierversuchen auf den Menschen (1:2) und die Berücksichtigung des erhöhten Atemvolumens (1:2) ergibt eine Konzentration von 0,007 mg Al/m^3^. Unter Einbezug des Preferred Value Approach wird ein MAK-Wert von 0,005 mg Al/m^3^ (A-Fraktion) für Aluminiumchlorhydrat (Dialuminiumchloridpentahydroxid) festgesetzt. Dieser MAK-Wert gilt auch für Aluminiumammoniumdisulfat und Aluminiumkaliumdisulfat, die ebenfalls nicht reizend sind (siehe Tabelle 2).

**MAK-Wert (A-Fraktion) **für **Aluminiumgluconat **und** Aluminiummaltolat**

Für Aluminiumgluconat und Aluminiummaltolat liegen weder Daten zur Augenreizwirkung noch eine entsprechende Einstufung nach GHS/CLP-Kriterien vor. Solange keine Daten vorliegen, gilt für diese Verbindungen als konservativer Ansatz der MAK-Wert von 0,0002 mg Al/m^3^. Stellen sich Aluminiumgluconat und Aluminiummaltolat als nicht augenreizend heraus, würde für sie beim weiteren Fehlen von Inhalationsstudien ein MAK-Wert von 0,005 mg Al/m^3^ festgesetzt werden.

**MAK-Wert (E-Fraktion)**

Da die MAK-Werte aus Tierversuchen mit A-Fraktionen abgeleitet wurden und keine Informationen zur Wirkung der E-Fraktion beim Menschen vorliegen, sind die MAK-Werte für die A-Fraktion auch für die E-Fraktion anzuwenden.

**Neurotoxizität**

Für den Menschen ist die Neurotoxizität von Aluminium nachgewiesen. Bei Arbeitern, die gegen schwerlösliche Aluminiumverbindungen (0,47 bis 0,7 mg/m^3^; Aluminiumgehalt des Schweißrauches mindestens 90 %, A-Fraktion) exponiert waren, traten präklinische neurotoxische Effekte auf, für welche ein NOAEL von 38 µg Al/g Kreatinin abgeleitet wurde. Mit löslichen Aluminiumverbindungen liegen keine epidemiologischen Studien zur Neurotoxizität vor. Da bei den löslichen Aluminiumverbindungen die Toxizität an der Lunge der sensitivste Endpunkt ist, und der MAK-Wert deutlich unter dem der schwerlöslichen Aluminiumverbindungen (Hartwig und MAK Commission 2025) liegt, schützt der MAK-Wert auch vor der Neurotoxizität.

**Spitzenbegrenzung. **Der kritische Effekt der löslichen Aluminiumverbindungen ist die Lungentoxizität, woraus eine Einstufung in Spitzenbegrenzungs-Kategorie II resultieren würde. Wegen der deutlichen Unterschiede der lokalen Wirkung vor allem am Auge werden jedoch für die verschiedenen löslichen Aluminiumverbindungen unterschiedliche Spitzenbegrenzungs-Kategorien festgelegt.

**Aluminiumchlorid**,** Aluminiumchlorid, basisch**,** Aluminiumchloridhydroxysulfat**,** Aluminiumcitrat**, **Aluminiumdiacetat**, **Aluminiumgluconat**, **Aluminiumlactat**, **Aluminiummaltolat**, **Aluminiumnitrat **und** Aluminiumsulfat**

Die löslichen Aluminiumverbindungen mit stark reizender bis ätzender Wirkung am Auge werden in Spitzenbegrenzungs-Kategorie I eingestuft. Die mögliche Reizwirkung am Auge und fehlende Daten zur Reizschwelle beim Menschen würden zunächst für den Basisüberschreitungsfaktor von 1 sprechen. Allerdings liegt zwischen dem MAK-Wert, bei dessen Ableitung die Reizwirkung mit einfloss, und der aus der 13-Wochen-Inhalationsstudie an Wistar-Ratten abgeleiteten NAEC ein relativ großer Abstand (Faktor 35), sodass ein Überschreitungsfaktor von 2 gerechtfertigt ist.

**Aluminiumchlorhydrat (Dialuminiumchloridpentahydroxid)**,** Aluminiumammoniumdisulfat **und** Aluminiumkaliumdisulfat**

Bei den als nicht augenreizend bewerteten löslichen Aluminiumverbindungen sind die Wirkungen an der Lunge maßgebend, die erst bei längerer Exposition entstehen. Sie werden in Spitzenbegrenzungs-Kategorie II eingestuft. Da die nur leichten Effekte bei der aus der sechsmonatigen Inhalationsstudie an F344-Ratten und Hartley-Meerschweinchen abgeleiteten LOAEC (Steinhagen et al. 1978) bei der Ableitung der NAEC bereits berücksichtigt wurden und der MAK-Wert nach Berücksichtigung der Extrapolation der Daten aus den Tierversuchen und des erhöhten Atemvolumens nur wenig von der NAEC abweicht, wird als Überschreitungsfaktor für die Gruppe der schwach augenreizenden löslichen Aluminiumverbindungen der Basisüberschreitungsfaktor dieser Kategorie von 2 festgelegt.

**Fruchtschädigende Wirkung. **Die epidemiologischen Studien können aufgrund methodischer Schwächen nicht zur Bewertung herangezogen werden. Es liegen keine Tierstudien zur fruchtschädigenden Wirkung nach inhalativer Exposition vor. Nach oraler, s.c. oder i.v. Gabe wirken lösliche Aluminiumverbindungen bei Ratten und Mäusen nicht teratogen.

In Studien zur pränatalen Entwicklungstoxizität nach Schlundsondengabe führen Aluminiumnitrat und Aluminiumchlorid bei Ratten sowie Aluminiumlactat und Aluminiumchlorid bei Mäusen zu entwicklungstoxischen Effekten bei gleichzeitiger Maternaltoxizität in Form von verringerter Körpergewichtszunahme. Im Vordergrund stehen verringerte Fetengewichte und verzögerte Ossifikationen. Aus den Studien kann kein NOAEL für pränatale Entwicklungstoxizität bei Ratten und Mäusen abgeleitet werden. Der LOAEL für Ratten liegt bei 13 mg Al/kg KG und Tag (Aluminiumnitrat) und für Mäuse bei 40 mg Al/kg KG und Tag (Aluminiumchlorid; Greim 2007). Die Unsicherheiten bei der Übertragbarkeit von Tierstudien mit oraler Gabe von Metallen auf die Inhalation am Arbeitsplatz sind bekannt (AGS 2010).

Mehrere Studien mit methodischen Schwächen zeigen entwicklungsneurotoxische Effekte wie verminderte Griffstärke der Vorder- und Hintergliedmaßen, reduzierte motorische Aktivität und reduzierte Schreckreaktion bei Ratten, Mäusen und Kaninchen, zum Teil ohne Maternaltoxizität.

In einer Trinkwasserstudie nach OECD-Prüfrichtlinie 426 an Sprague-Dawley-Ratten mit Aluminiumcitrat kam es bei den Nachkommen ab 102 mg Al/kg KG und Tag zu neuromuskulären Effekten, dosisabhängig erniedrigter Griffstärke bei Vorder- und Hintergliedmaßen sowie Beeinträchtigung der Fußspreizung. Der NOAEL lag bei 27 mg Al/kg KG und Tag (Poirier et al. 2011; ToxTest Alberta Research Council Inc. 2010). Die gemessenen Parameter nach OECD-Prüfrichtlinie 426 gelten als relativ insensitiv (Paparella et al. 2020), insbesondere für kognitive Effekte, sodass hier eine Datenlücke für kognitive Effekte gegeben ist. Daher könnte eine höhere Empfindlichkeit der Feten bzw. Nachkommen gegeben sein, die aber aufgrund der genannten Unsicherheiten nicht zuverlässig belegt werden kann. Jedoch ist verglichen mit dem NOAEL von 27 mg/kg KG und Tag angesichts der niedrigen MAK-Werte von 0,0002 bzw. 0,005 mg Al/m^3^, die unter der Annahme eines Atemvolumens von 10 m^3^ pro 8 h höchstenfalls eine Aufnahme von 0,002 bzw. 0,05 mg Aluminium pro Arbeitstag ergeben (Berechnung siehe unten), ein ausreichend großer Abstand gegeben.

Ausgehend von den MAK-Werten von 0,0002 bzw. 0,005 mg Al/m^3^ ergibt sich unter der Annahme eines Atemvolumens von 10 m^3^ pro 8 h, einer 100%igen Deposition, einer 5%igen inhalativen Resorption (Abschnitt 3.1.1.1) und einem Blutvolumen von 5 l eine Blutkonzentration von 0,02 bzw. 0,5 µg Al/l zusätzlich zur Hintergrundbelastung (0,2 bzw. 5 µg/m^3^ × 10 m^3^ × 0,05 / 5 l). Dies entspricht einem konservativen Ansatz, da nicht berücksichtigt wird, dass sich Aluminium in andere Gewebe umverteilt, 60 % mit dem Urin ausgeschieden werden und die Ausscheidung bereits während der achtstündigen Exposition zu geringeren Blutspiegeln als den berechneten führt. Mittels der Daten von Goullé et al. (2005) und Nisse et al. (2017) lässt sich ausgehend von der zusätzlichen Aluminiumkonzentration im Blut unter der Annahme eines linearen Zusammenhangs eine Erhöhung der Aluminiumkonzentration im Urin von 0,2 % bzw. 0,1 % (für den MAK-Wert von 0,0002 mg Al/m^3^ E) bzw. von 4,0 % bzw. 2,7 % (für den MAK-Wert von 0,005 mg Al/m^3^ E) bezogen auf den Biologischen Arbeitsstoff-Referenzwert (BAR) von 15 µg Al/g Kreatinin berechnen (siehe Tabelle 18).

**Tab. 18:** Aluminiumkonzentrationen in Blut und Serum bei Exposition in Höhe der MAK-Werte löslicher Aluminiumverbindungen von 0,0002 mg Al/m^3^ bzw. 0,005 mg Al/m^3^

**Literatur**	**Blut, Median** **[µg/l] **	**Urin, Median** **[µg/g Kreatinin]**	**zusätzliches Al im Blut** **[µg/l]**	**zusätzliches Al im Urin^a)^** **[µg/g Kreatinin]**	**zusätzlicher Anteil von Al im Urin bezogen auf BAR von 15 µg/g Kreatinin**
**MAK-Wert: 0,0002 mg Al/m^3^** **Annahme: 5%ige inhalative Resorption** ^b)^					
Goullé et al. 2005	1,3	1,6	0,02	0,025	0,2 %
Nisse et al. 2017	3,1	2,4	0,02	0,015	0,1 %
**MAK-Wert: 0,005 mg Al/m^3^** **Annahme: 5%ige inhalative Resorption** ^b)^					
Goullé et al. 2005	1,3	1,6	0,5	0,6	4,0 %
Nisse et al. 2017	3,1	2,4	0,5	0,4	2,7 %

^a)^ Annahme linearer Zusammenhang, Umrechnung: Dreisatz

^b)^ Daten von Aluminium-exponierten Beschäftigen am Arbeitsplatz (Pierre et al. 1995), siehe Abschnitt 3.1.1.1

Die zusätzlich bei Exposition in Höhe des MAK-Wertes von 0,0002 bzw. 0,005 mg Al/m^3^ hinzukommende Blutkonzentration trägt nicht nennenswert zu einer Erhöhung über den Hintergrundbereich der Aluminiumkonzentration im Blut (95. Perzentile von 6,4 bis 33,3 µg Al/l; Alimonti et al. 2005; Goullé et al. 2005) bei. Dies gilt auch für den hinzukommenden Anteil im Urin bezogen auf den BAR von 15 µg/g Kreatinin.

Die Menge des Aluminiums, die den Fetus erreicht, ist vermutlich abhängig von der Konzentration im maternalen Blut (EFSA 2008). Bei einer Halbwertszeit von 5 h ist jedoch keine bedeutende Akkumulation im Blut zu erwarten.

Daher werden die löslichen Aluminiumverbindungen mit einem MAK-Wert von 0,0002 mg Al/m^3^ E sowie mit einem MAK-Wert von 0,005 mg Al/m^3^ E der Schwangerschaftsgruppe C zugeordnet.

**Krebserzeugende Wirkung. **Die epidemiologischen Untersuchungen lassen keine Aussage zum kanzerogenen Potenzial löslicher Aluminiumverbindungen zu.

Aus den Daten am Menschen kann kein Zusammenhang zwischen der Entstehung von Brustkrebs und der Verwendung aluminiumhaltiger Antitranspirantien abgeleitet werden. Aluminium wird offensichtlich als Konsequenz veränderter Transportvorgänge vermehrt im Tumorgewebe eingelagert, sodass eine erhöhte Konzentration im Tumorgewebe nicht als Hinweis auf einen ursächlichen Zusammenhang mit Brustkrebs zu werten ist.

Valide Langzeituntersuchungen zur kanzerogenen Wirkung liegen nicht vor. Aus den teilweise ungenügend berichteten Tierstudien ergibt sich kein entsprechender Verdacht.

Lösliche Aluminiumverbindungen werden daher nicht in eine Kategorie für Kanzerogene eingestuft.

**Keimzellmutagene Wirkung. **Aluminium ist bei Bakterien und Säugetierzellen nicht mutagen. In vitro und in vivo werden klastogene und aneugene Wirkungen sowie positive Indikatortests meist in Anwesenheit von Zytotoxizität beobachtet. Unterhalb zytotoxischer Konzentrationen werden in validen In-vivo-Studien keine Mikronuklei oder Chromosomenaberrationen induziert. Die durch lösliche Aluminiumverbindungen verursachte genotoxische Wirkung, die meist nahe bei oder mit zytotoxischen Konzentrationen auftritt, wird durch die Bindung des Aluminiumions an DNA, RNA und Proteine mit der daraus resultierenden Induktion von oxidativem Stress, DNA-Strukturveränderungen und Bindung an Mikrotubuli-assoziierten Proteinen hervorgerufen. Zytotoxizität kann zur Entgleisung zellulärer Mechanismen führen, wodurch wichtige Schutzwirkungen gegen Genotoxizität vermindert und dadurch genotoxische Effekte ermöglicht werden. Studien an Keimzellen liegen nicht vor. Anhand der vorliegenden Daten werden lösliche Aluminiumverbindungen nicht in eine Kategorie für Keimzellmutagene eingestuft.

**Hautresorption. **Die Aufnahme der löslichen Aluminiumverbindungen (Abschnitt 3.1.1.3) über die Haut liegt bezogen auf den BAR weit im Hintergrundbereich und damit auch sehr weit unterhalb des BAT-Werts von 50 µg/g Kreatinin (Klotz et al. 2018). Die sehr geringe Aufnahme löslicher Aluminiumverbindungen über die Haut wird auch durch die Probandenstudie mit 14-tägiger Applikation eines Aluminiumchlorhydrat-Antitranspirants (Letzel et al. 2020) bestätigt. Die löslichen Aluminiumverbindungen werden deshalb nicht mit „H“ markiert.

**Sensibilisierende Wirkung. **Es liegen keine Daten beruflich bedingter Kontaktdermatitis durch lösliche Aluminiumverbindungen vor. Obwohl außerberuflich umfangreiche Expositionsmöglichkeiten, beispielsweise durch Verwendung von Antitranspirantien, gegen lösliche Aluminiumverbindungen bestehen, wurde nach wie vor nur in wenigen Fällen über eine Sensibilisierung berichtet. Bei mehreren Fällen stand die Sensibilisierung im Zusammenhang mit einer s.c. Applikation von Aluminiumsalzen als Bestandteil von Vakzinen, die unter Arbeitsplatzbedingungen nicht relevant ist. Tierexperimentelle Untersuchungen zur sensibilisierenden Wirkung löslicher Aluminiumverbindungen waren negativ. Zur atemwegssensibilisierenden Wirkung löslicher Aluminiumverbindungen liegen keine neuen Daten vor. Es erfolgt daher weiterhin keine Markierung mit „Sh“ oder „Sa“.
